# Supplementary material for: Epigenetic lockdown of type I interferon sensing and signalling in human pluripotent cells
Source: Nat Commun. 2026 Jun 16;17:7660. doi: 10.1038/s41467-026-74147-0 (PMC13434735; doi:10.1038/s41467-026-74147-0)
Supplement: Supplementary file 1 [file 41467_2026_74147_MOESM1_ESM.pdf]

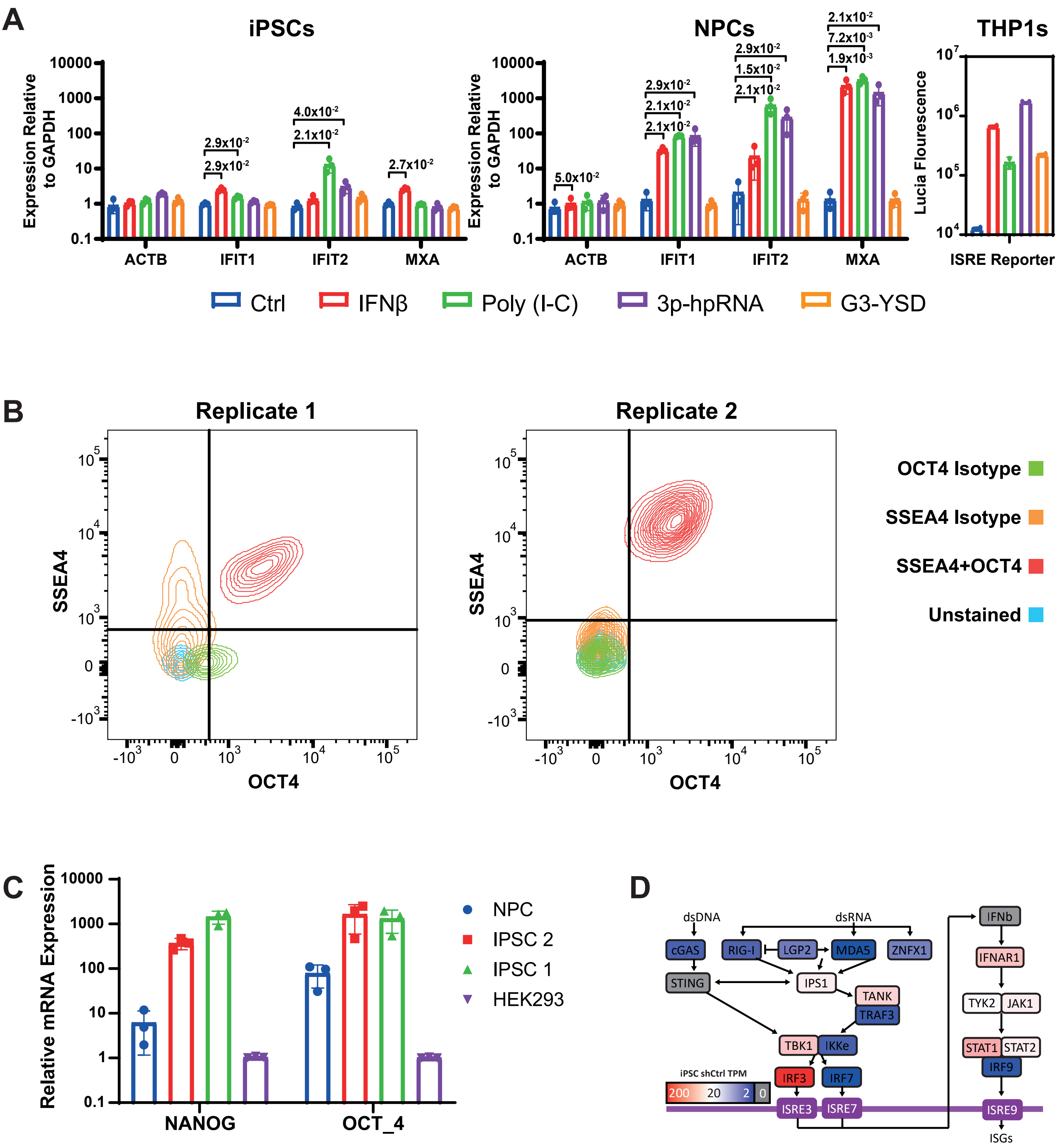
A
iPSCs
NPCs
THP1s
Expression Relative to GAPDH
Lucia Flourescence
ISRE Reporter
ACTB
IFIT1
IFIT2
MXA
Ctrl
IFNβ
Poly (I-C)
3p-hpRNA
G3-YSD
B
Replicate 1
Replicate 2
SSEA4
OCT4
OCT4 Isotype
SSEA4 Isotype
SSEA4+OCT4
Unstained
C
Relative mRNA Expression
NANOG
OCT_4
NPC
IPSC 2
IPSC 1
HEK293
D
dsDNA
dsRNA
cGAS
RIG-I
LGP2
MDA5
ZNFX1
STING
IPS1
TANK
TRAF3
TBK1
IKKe
IRF3
IRF7
ISRE3
ISRE7
iPSC shCtrl TPM
IFNb
IFNAR1
TYK2
JAK1
STAT1
STAT2
IRF9
ISRE9
ISGs

Supplementary Figure 1: IFN-I responses in iPSCs versus NPCs and impacts of MPP8 depletion on gene expression.

(A) RT-qPCR showing gene expression relative to GAPDH in iPSCs (left) and NPCs (right) after treatment with control (blue), IFNβ (red), poly(I:C) (green), 3p-hpRNA (purple), and G3-YSD (orange), N=3 independent experiments. Annotated with BH-adjusted p-values from two-tailed paired t tests. (B) Contour plots for isotype controls in WT iPSC 1 transduced with either shCtrl or shMPP8-expressing lentiviral vectors shown in Figure 3C alongside unstained cells and dual stained OCT4/SSEA4. N=2 independent experiments (C) Relative mRNA expression of OCT4, NANOG, and ACTB in iPSCs relative to GAPDH in HEK 293 cells. N=3 independent experiments. (D) Colour coded pathway diagram indicating average gene expression levels (transcripts per million (TPM)) of nucleic acid sensing components from N=3 independent replicates of RNA seq analysis of iPSCs treated with shCtrl.

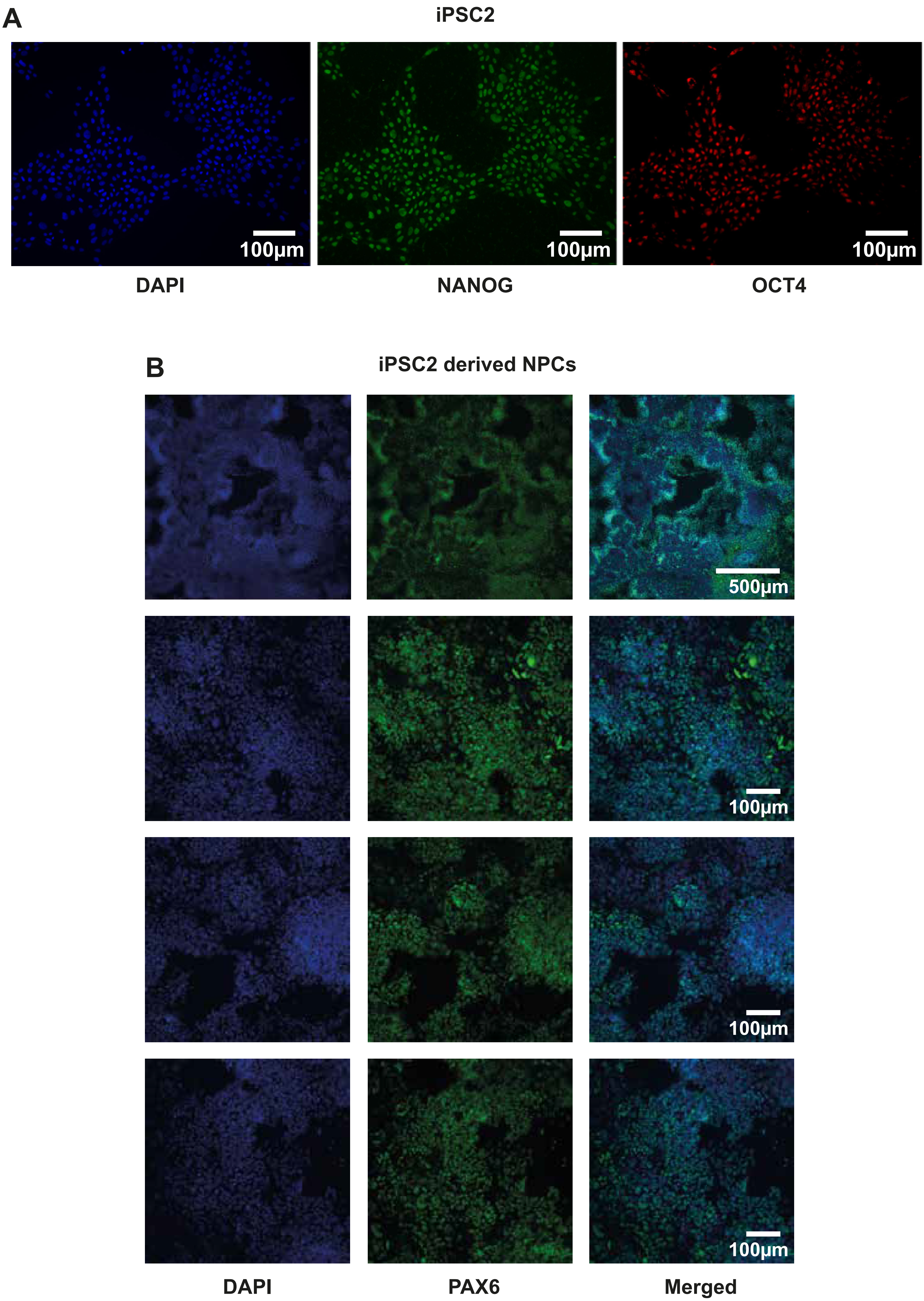
A
iPSC2
100µm
100µm
100µm
DAPI
NANOG
OCT4
B
iPSC2 derived NPCs
500µm
100µm
100µm
100µm
DAPI
PAX6
Merged

Supplementary Figure 2: Validation of pluripotency and differentiation markers expression in iPSCs and NPCs.

Intracellular staining (ICS) followed by microscopy of WTC11 iPSCs (A, n=1) and WTC11 iPSCs differentiated into NPCs (B, N=4) cells stained with DAPI (left), PAX6 (middle), and both DAPI and PAX6 (right).

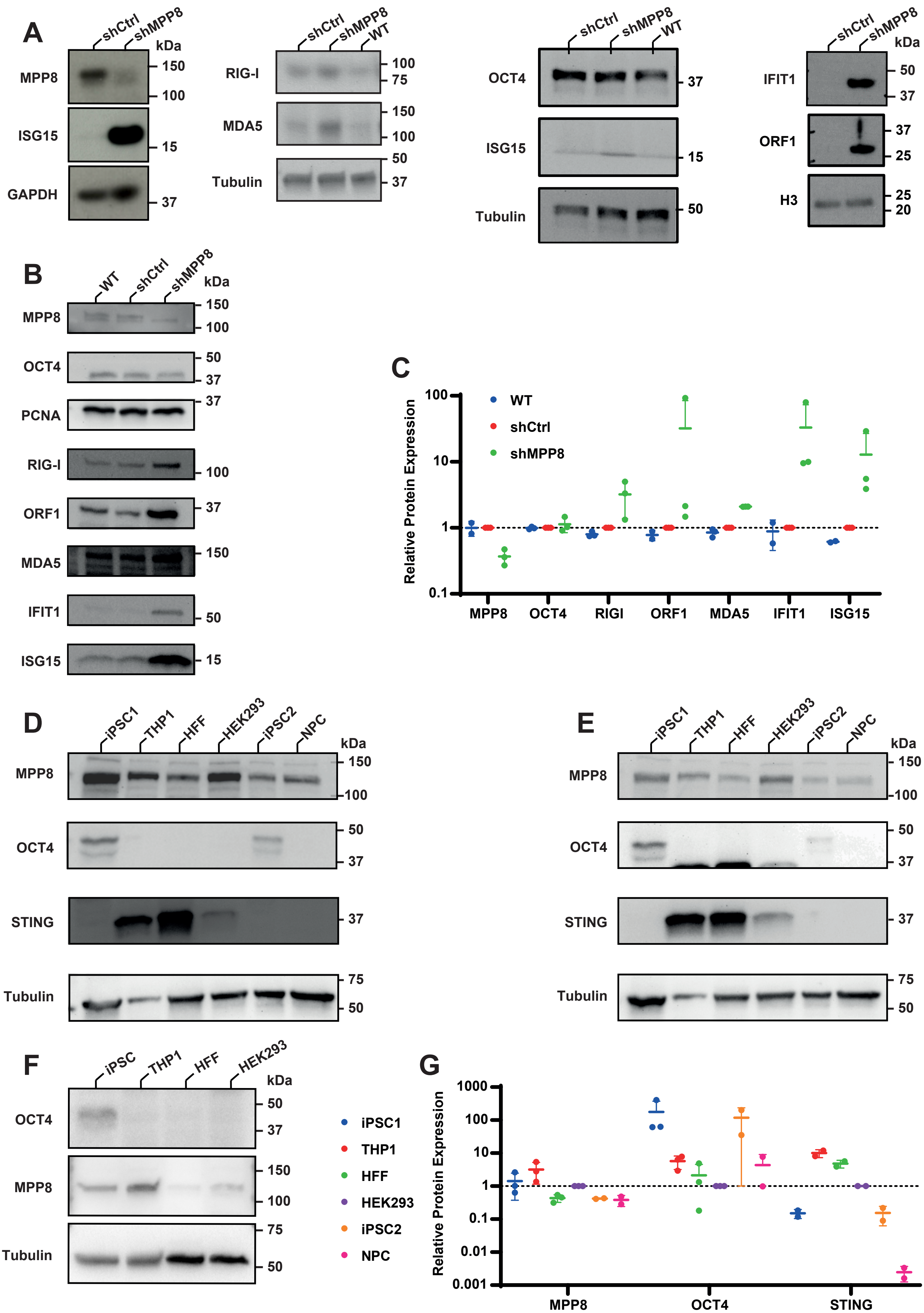

A
shCtrl
shMPP8
WT
kDa
MPP8
ISG15
GAPDH
RIG-I
MDA5
Tubulin
OCT4
IFIT1
ORF1
H3
B
C
Relative Protein Expression
WT
shCtrl
shMPP8
MPP8
OCT4
RIGI
ORF1
MDA5
IFIT1
ISG15
PCNA
RIG-I
D
E
iPSC1
THP1
HFF
HEK293
iPSC2
NPC
STING
F
iPSC
G

Supplementary Figure 3: Additional western blots.

Replicates 2 (A) and 3 (B) of western blot analysis of iPSCs following MPP8 depletion. In combination with main figures this totals N=3 for MPP8, OCT4, ORF1, IFIT1 and ISG15 and N=2 for RIG-I and MDA5. (C) Quantification of western blots for iPSCs following MPP8 depletion. Three replicates (D, E, & F) of western blot analysis of multiple cell types for expression of MPP8 (N=3), OCT4 (N=3), and STING (N=2). (G) Quantification of western blots for protein expression by cell type.

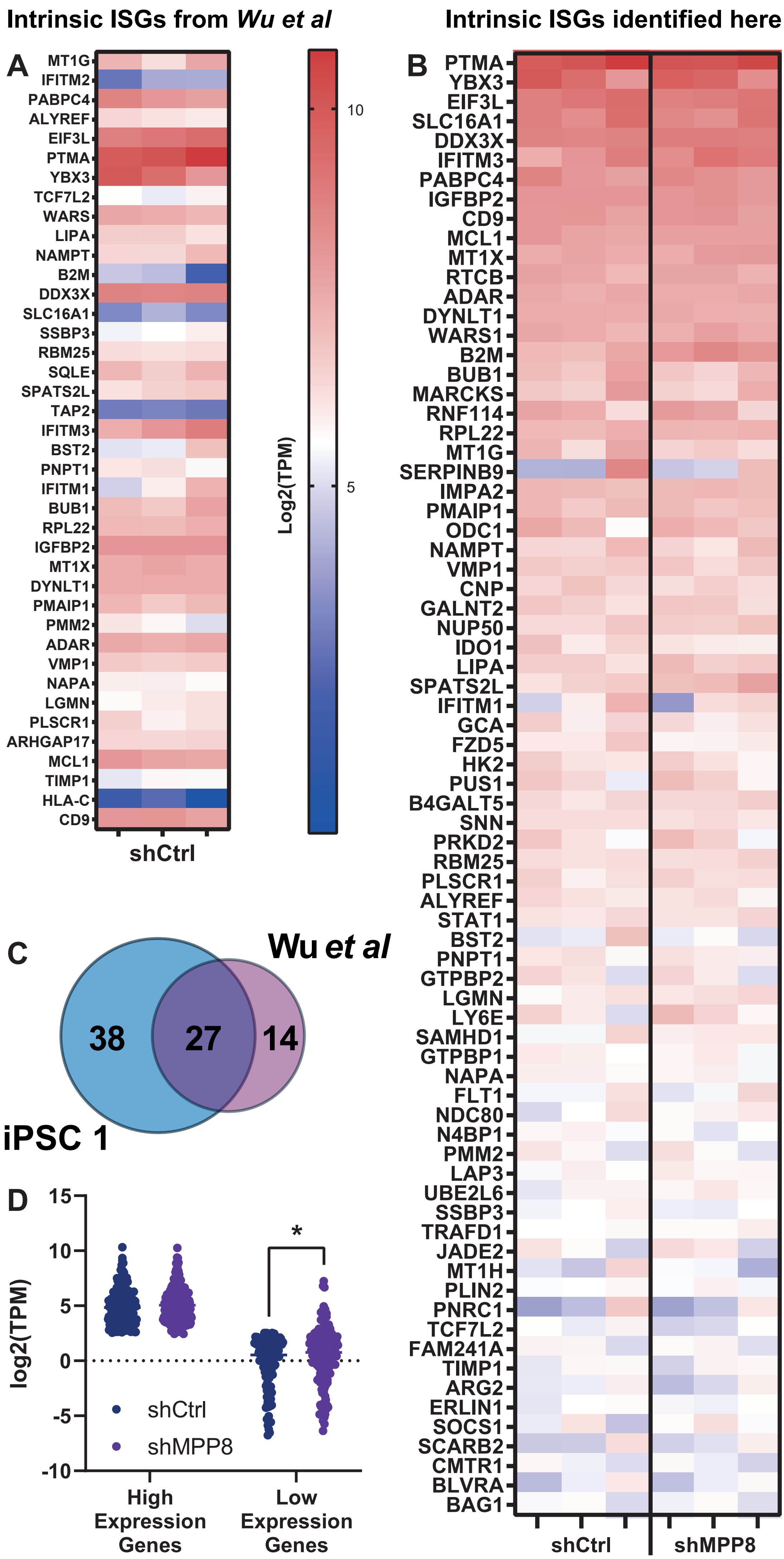

Intrinsic ISGs from *Wu et al*
Intrinsic ISGs identified here
A
MT1G
IFITM2
PABPC4
ALYREF
EIF3L
PTMA
YBX3
TCF7L2
WARS
LIPA
NAMPT
B2M
DDX3X
SLC16A1
SSBP3
RBM25
SQLE
SPATS2L
TAP2
IFITM3
BST2
PNPT1
IFITM1
BUB1
RPL22
IGFBP2
MT1X
DYNLT1
PMAIP1
PMM2
ADAR
VMP1
NAPA
LGMN
PLSCR1
ARHGAP17
MCL1
TIMP1
HLA-C
CD9
shCtrl
10
5
Log2(TPM)
B
PTMA
YBX3
EIF3L
SLC16A1
DDX3X
IFITM3
PABPC4
IGFBP2
CD9
MCL1
MT1X
RTCB
ADAR
DYNLT1
WARS1
B2M
BUB1
MARCKS
RNF114
RPL22
MT1G
SERPINB9
IMPA2
PMAIP1
ODC1
NAMPT
VMP1
CNP
GALNT2
NUP50
IDO1
LIPA
SPATS2L
IFITM1
GCA
FZD5
HK2
PUS1
B4GALT5
SNN
PRKD2
RBM25
PLSCR1
ALYREF
STAT1
BST2
PNPT1
GTPBP2
LGMN
LY6E
SAMHD1
GTPBP1
NAPA
FLT1
NDC80
N4BP1
PMM2
LAP3
UBE2L6
SSBP3
TRAFD1
JADE2
MT1H
PLIN2
PNRC1
TCF7L2
FAM241A
TIMP1
ARG2
ERLIN1
SOCS1
SCARB2
CMTR1
BLVRA
BAG1
shCtrl
shMPP8
C
Wu *et al*
38
27
14
iPSC 1
D
15
10
5
0
-5
-10
log2(TPM)
*
shCtrl
shMPP8
High Expression Genes
Low Expression Genes

Supplementary Figure 4: Intrinsic ISG expression is unaffected by MPP8 depletion.

(A) Heatmap showing log2(TPM) of a list of highly expressed ISGs in iPSCs from Figure 2B of Wu et al. 2018. Scale and colouring were kept consistent with this Figure while TPM values were used from N=3 shCtrl conditions of our RNA sequencing experiments Red signifies high log2TPM (10.779) while blue signifies low log2TPM (0.394). (B) Heatmap showing intrinsically expressed ISGs identified in our iPSCs following the steps from Wu et al. 2018. Heatmaps for N=3 replicates of iPSCs treated with shCtrl (left) and shMPP8 (right) are shown. (C) Venn diagram showing overlap between ISGs in (A) and (B). (D) Dot plot showing log2 TPM values for high (top 50%) and low (bottom 50%) expression genes under shCtrl (blue) and shMPP8 *(purple) conditions. Each dot represents an individual gene, with the y-axis indicating log2(TPM) values. Unpaired T tests were performed between shCtrl and shMPP8, * = P<0.05, P= 0.354267285 and 0.012756479.*

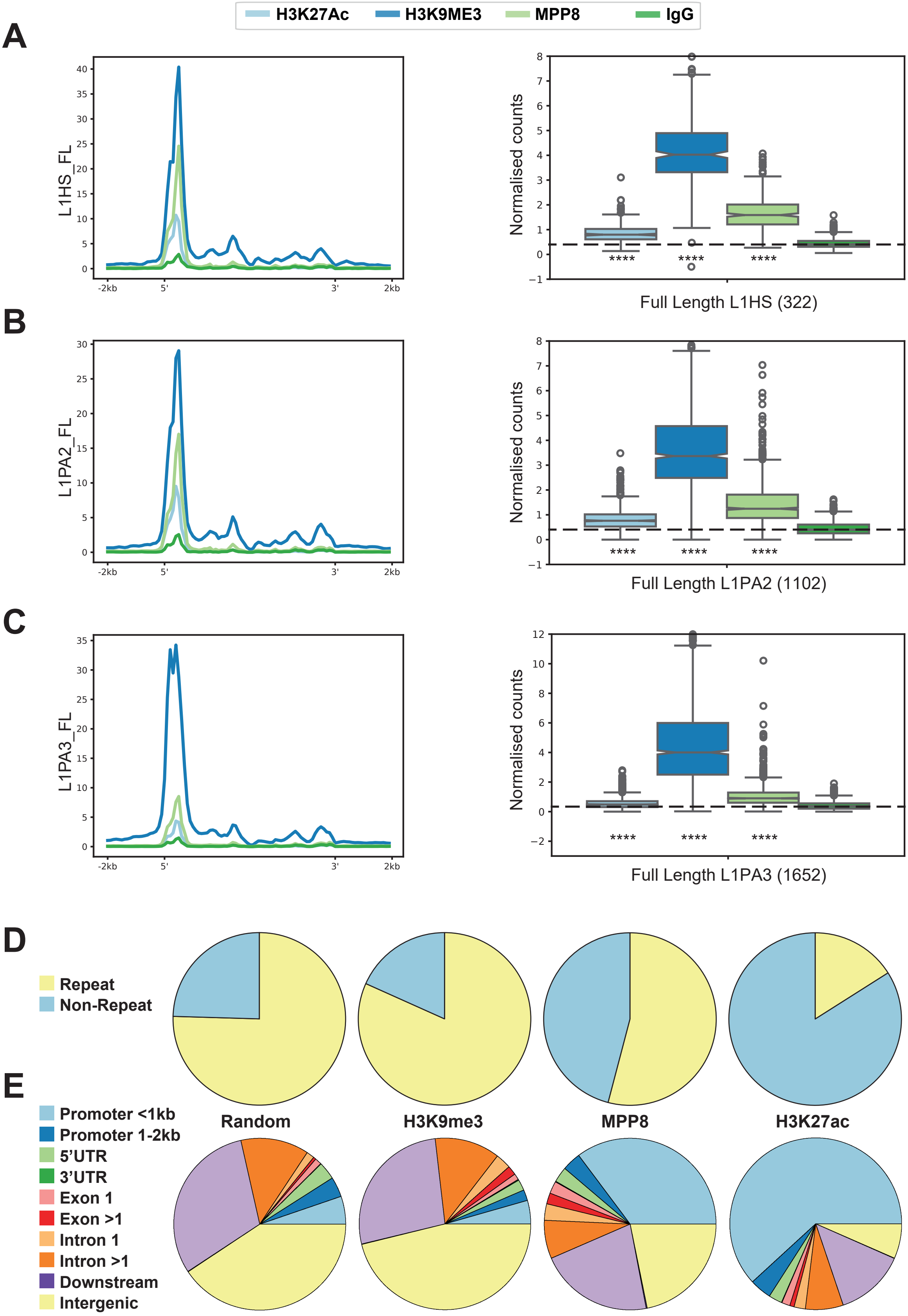

A
B
C
D
E
H3K27Ac
H3K9ME3
MPP8
IgG
L1HS_FL
L1PA2_FL
L1PA3_FL
-2kb
5'
3'
2kb
Normalised counts
****
Full Length L1HS (322)
Full Length L1PA2 (1102)
Full Length L1PA3 (1652)
Repeat
Non-Repeat
Promoter <1kb
Promoter 1-2kb
5'UTR
3'UTR
Exon 1
Exon >1
Intron 1
Intron >1
Downstream
Intergenic
Random
H3K9me3
MPP8
H3K27ac

Supplementary Figure 5: CUT & Tag binding at young LINE-1s and peak distribution over genes and repeats.

(A-C) Profile plots (left) and box and whisker plots (right) derived from CUT & Tag data showing normalised read density over full length copies of L1HS (332 loci, A), L1PA2 (1102 loci, B), and L1PA3 (1652 loci, C) and a 2kb flanking region. Plots produced for IgG (control), H3K9me3, H3K27ac, and MPP8 using multi-mapping reads. Enrichment was normalised to IgG for box plots and annotated with p-values from two-tailed unpaired t tests. P = 1.04E-60, 3.27E-214, 1.38E-130, 3.11E-100, 0, 3.14E-282, 2.50E-26, 0, and 4.45E-215. Centre line indicates median with boxes for IQR, error bars indicate the 1.5x IQR, and circles indicate outliers. (D) Pie charts showing the proportion of our CUT & Tag peaks intersecting with various annotations from repeats (D) or genes (E) for peaks from H3K9me3, MPP8, and H3K27ac alongside 10,000 randomly generated regions.

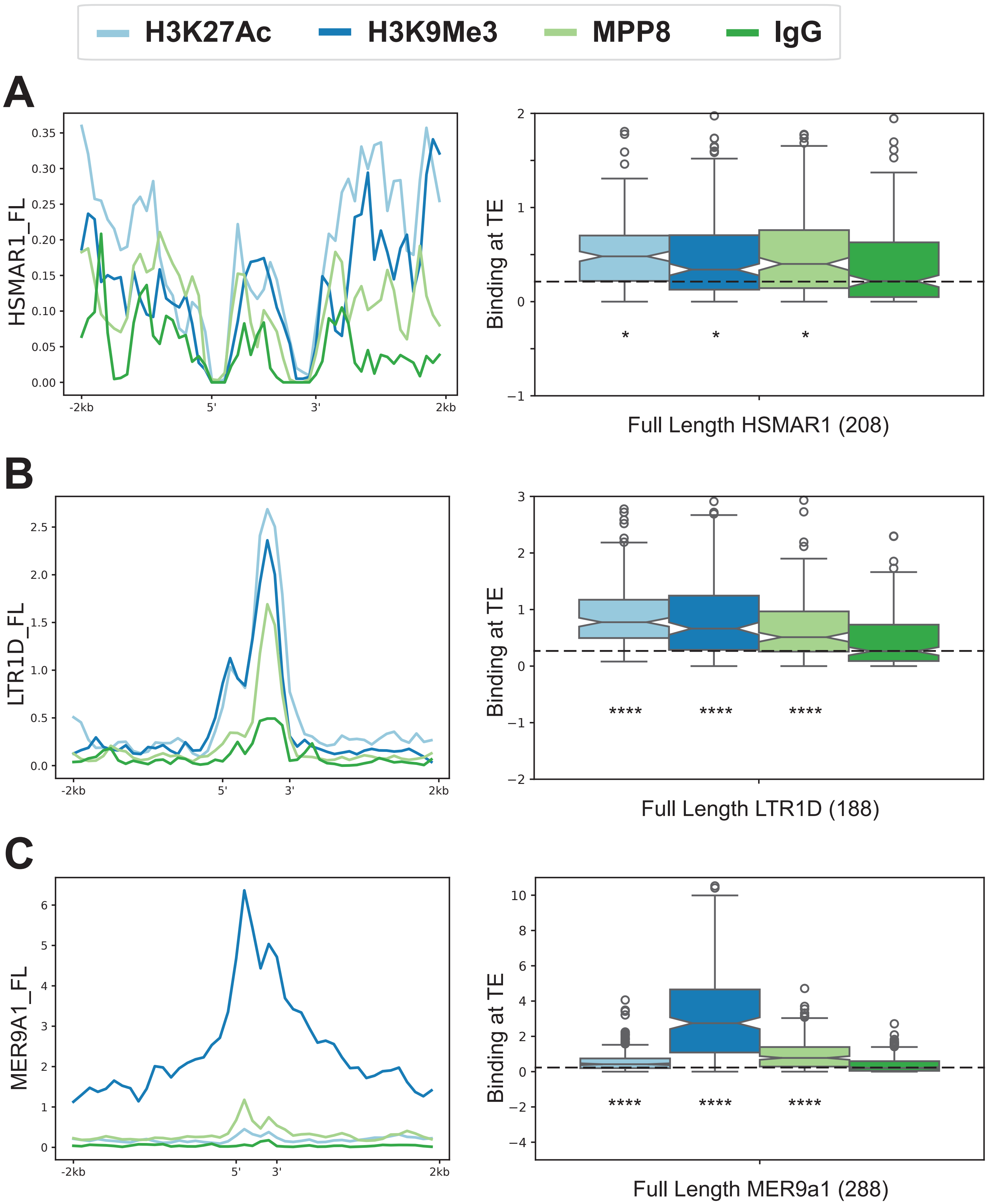

H3K27Ac
H3K9Me3
MPP8
IgG
A
HSMAR1_FL
-2kb
5'
3'
2kb
Binding at TE
*
*
*
Full Length HSMAR1 (208)
B
LTR1D_FL
****
****
****
Full Length LTR1D (188)
C
MER9A1_FL
****
****
****
Full Length MER9a1 (288)

Supplementary Figure 6: CUT & Tag binding at young LINE-1s and peak distribution over genes and repeats.

(A-C) Profile plots (left) and box and whisker plots (right) derived from CUT & Tag data showing normalised read density over HSMAR1 (208 loci, A), LTR1D (188 loci, B), and MER9A1 (288 loci, C) and a 2kb flanking region. Plots produced for IgG (control), H3K9me3, H3K27ac, and MPP8 using multi-mapping reads. Enrichment was normalised to IgG for box plots and annotated with p-values from two-tailed unpaired t tests. P = 8.22E-12, 0.396829063, 0.025211635, 4.34E-06, 0.147611666, 0.147611666, 6.15E-08, 1.29E-32, and 1.86E-12. Centre line indicates median with boxes for IQR, error bars indicate the 1.5x IQR, and circles indicate outliers.

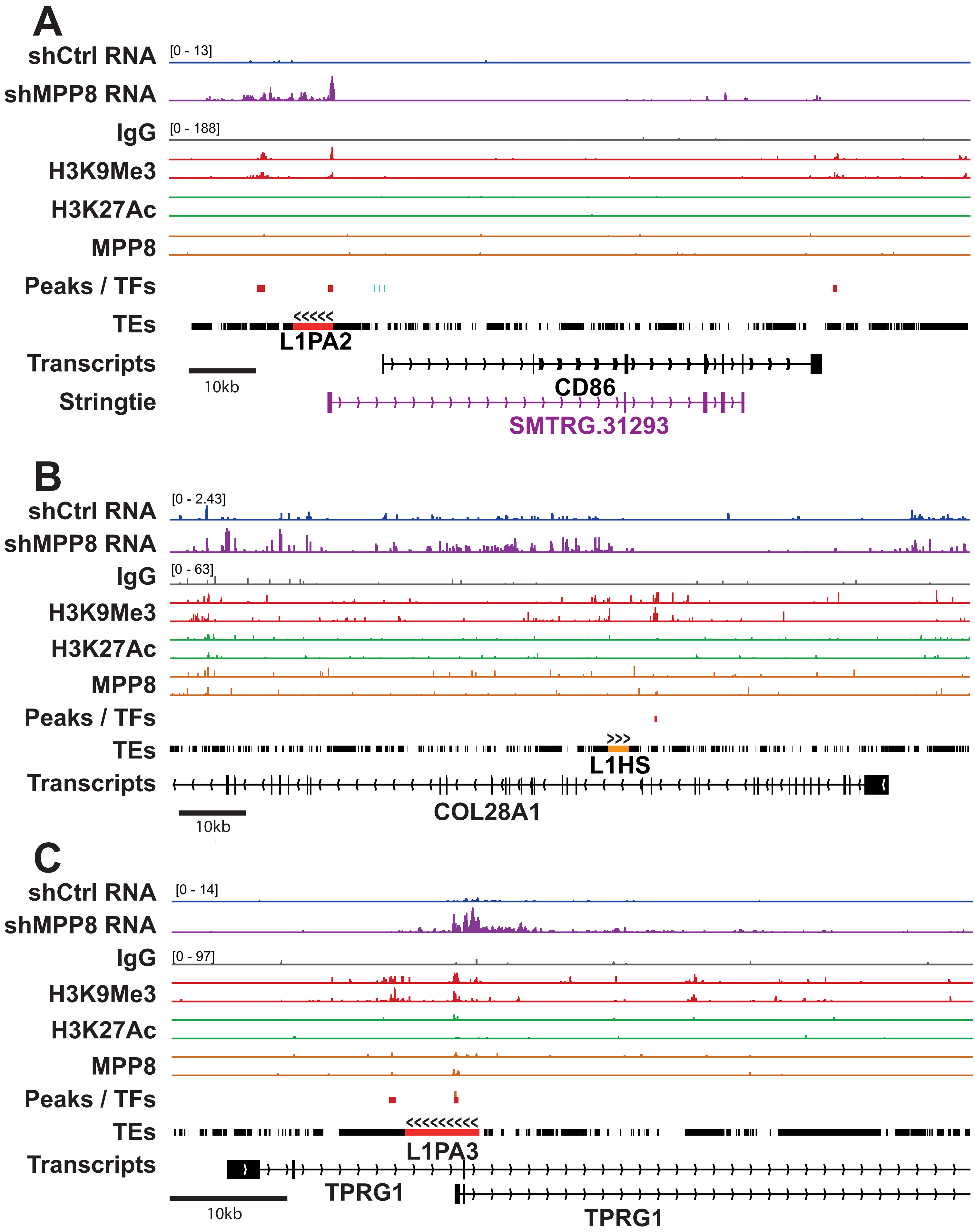
A
shCtrl RNA
[0 - 13]
shMPP8 RNA
IgG
[0 - 188]
H3K9Me3
H3K27Ac
MPP8
Peaks / TFs
TEs
<<<<<
L1PA2
Transcripts
10kb
CD86
Stringtie
SMTRG.31293
B
shCtrl RNA
[0 - 2.43]
shMPP8 RNA
IgG
[0 - 63]
H3K9Me3
H3K27Ac
MPP8
Peaks / TFs
TEs
>>>
L1HS
Transcripts
COL28A1
10kb
C
shCtrl RNA
[0 - 14]
shMPP8 RNA
IgG
[0 - 97]
H3K9Me3
H3K27Ac
MPP8
Peaks / TFs
TEs
<<<<<<<<<<
L1PA3
Transcripts
TPRG1
10kb
TPRG1

Supplementary Figure 7: Integrative Genomics Viewer (IGV) tracks over MPP8 regulated non-ISGs.

IGV tracks demonstrating RNA-seq and CUT & Tag coverage across gene loci accompanied gene and TE annotations. Coverage plots are shown for shCtrl-RNA (blue), shMPP8-RNA (purple), IgG (grey), H3K9me3 (red), H3K27ac (green), and MPP8 (orange). Scaling for these tracks is indicated on the plot in square brackets with RNA and CUT & Tag data scaled separately. Scale bars indicate the size of the indicated region for each track in the bottom left of each plot. Differentially expressed TEs as per SQuIRE are shown in red. Previously unidentified transcripts and differentially expressed transcripts form Stringtie are shown in purple. Genes with multiple transcript isoforms have been compressed. IGV plots are shown for (A) CD86, (B) COL28A1, (C) TPRG1.

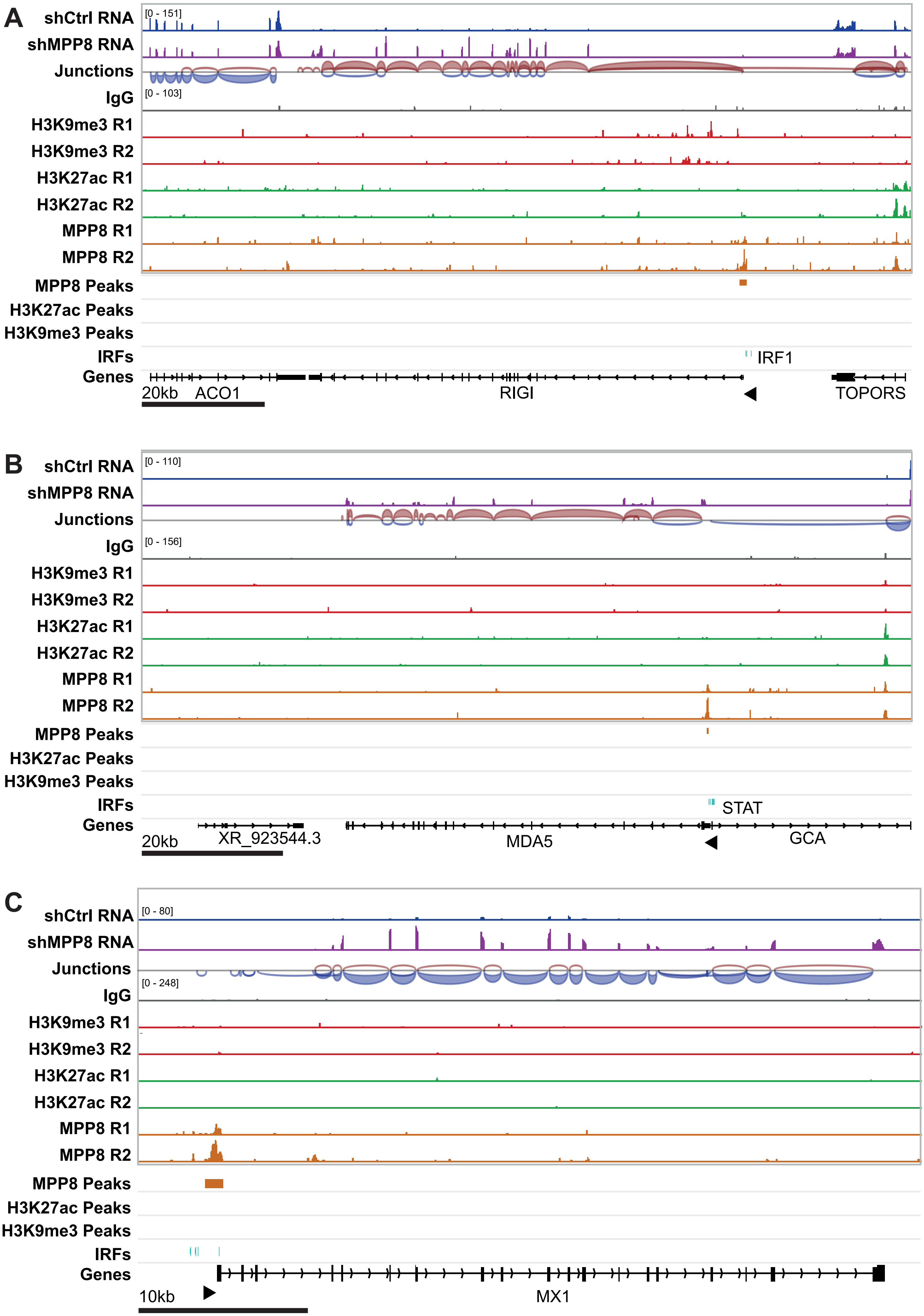
A
shCtrl RNA
shMPP8 RNA
Junctions
IgG
H3K9me3 R1
H3K9me3 R2
H3K27ac R1
H3K27ac R2
MPP8 R1
MPP8 R2
MPP8 Peaks
H3K27ac Peaks
H3K9me3 Peaks
IRFs
Genes
[0 - 151]
[0 - 103]
IRF1
20kb
ACO1
RIGI
TOPORS
B
shCtrl RNA
shMPP8 RNA
Junctions
IgG
H3K9me3 R1
H3K9me3 R2
H3K27ac R1
H3K27ac R2
MPP8 R1
MPP8 R2
MPP8 Peaks
H3K27ac Peaks
H3K9me3 Peaks
IRFs
Genes
[0 - 110]
[0 - 156]
STAT
20kb
XR_923544.3
MDA5
GCA
C
shCtrl RNA
shMPP8 RNA
Junctions
IgG
H3K9me3 R1
H3K9me3 R2
H3K27ac R1
H3K27ac R2
MPP8 R1
MPP8 R2
MPP8 Peaks
H3K27ac Peaks
H3K9me3 Peaks
IRFs
Genes
[0 - 80]
[0 - 248]
10kb
MX1

Supplementary Figure 8: Integrative Genomics Viewer (IGV) tracks over MPP8 regulated ISGs.

IGV tracks demonstrating RNA-seq and CUT & Tag coverage across gene loci accompanied gene and TE annotations. Coverage plots are shown for shCtrl-RNA (blue), shMPP8-RNA (purple), Junctions (red/blue), IgG (grey), H3K9me3 (red), H3K27ac (green), and MPP8 (orange) alongside peaks. Scaling for these tracks is indicated on the plot in square brackets with RNA and CUT & Tag data scaled separately. Scale bars indicate the size of the indicated region for each track in the bottom left of each plot. Genes with multiple transcript isoforms have been compressed. IGV plots are shown for (A) RIG-I, (B) MDA5, and (C) MX1.

Supplementary Table 1: qPCR Primers

Target	Orientation	Sequence
L1HS	Forwards	AACCTCTGCAGACTTAAGTGTC
	Reverse	AGGCAAGTCTGCCCGTTCTCAGA
L1PA2	Forwards	CACCTGGAAAGTCGAGTCAC
	Reverse	GGTGTGGGATATAATCTCGTGG
L1PA4	Forwards	TCACCAATATCCGCTGTTCTG
	Reverse	GTCTGTTGGAGTTTACTGGAGG
GAPDH	Forwards	GGGAAACTGTGGCGTGAT
	Reverse	GGAGGAGTGGGTGTCGCTGTT
ACTB	Forwards	CACCATTGGCAATGAGCGGTTC
	Reverse	AGGTCTTTGCGGATGTCCACGT
MPP8	Forwards	TGCCTGTATCTGCCCAAAC
	Reverse	CCTTGTCAGAATCATGCCTTTTC
IFIT1	Forwards	CCTGAAAGGCCAGAATGAGG
	Reverse	TCCACCTTGTCCAGGTAAGT
IFIT2	Forwards	ACTATGCCTGGGTCTACTATCA
	Reverse	ACTATGCCTGGGTCTACTATCA
MXA	Forwards	ATCCTGGGATTTTGGGGCTT
	Reverse	ATCCTGGGATTTTGGGGCTT
OCT_4	Forwards	TTCAGCCAAACGACCATC
	Reverse	GGAAAGGGACCGAGGAGTA
NANOG	Forwards	TTTGTGGGCCTGAAGAAAACT
	Reverse	AGGGCTGTCCTGAATAAGCAG

Supplementary Table 2: Western blotting antibodies

Target	Species	Supplier	ID
ACTIN-HRP	rabbit	Cell Signalling Technology	5125
DICER (D38E7)	rabbit	Cell Signalling Technology	5362S
DNMT1	rabbit	Cell Signalling Technology	5032S
J2 dsRNA	rabbit	Absolute antibodies	Ab01299-23.0
GAPDH-HRP	rabbit	Cell Signalling Technology	8884T
IFIT1	rabbit	Cell Signalling Technology	14769S
IFITM3	rabbit	Cell Signalling Technology	59212
IRF3	rabbit	Cell Signalling Technology	4302
KLF4	rabbit	Cell Signalling Technology	4038
LGP2	rabbit	Cell Signalling Technology	12869S
MAVS	rabbit	Cell Signalling Technology	3993S
MDA5	rabbit	Cell Signalling Technology	5321S
Mouse HRP	horse	Cell Signalling Technology	7076S
MPP8	rabbit	Proteintech	16796-1-AP
MX1	rabbit	Cell Signalling Technology	37849S
OAS1	rabbit	Cell Signalling Technology	14498
Oct-04	rabbit	Cell Signalling Technology	2840
PCNA	rabbit	Cell Signalling Technology	13110S
Phospho-IRF3	rabbit	Cell Signalling Technology	29047
Rabbit HRP	goat	Cell Signalling Technology	7074S
RIG-I	rabbit	Cell Signalling Technology	3743S
Tubulin HRP	rabbit	Abcam	ab21058

Supplementary Table 3: antibodies for microscopy / ICS

Target	Species	ID	Supplier
OCT4	Mouse	653703	Biolegend
SSEA4	Mouse	46-8843-42	Thermo
ISG15	Rat	IC8044G	Bio Techn
PAX6	Rabbit	PSI-5581	Biolegend
NANOG	Mouse	ab80892	Abcam
J2 dsRNA	rabbit	Ab01299-23.0	Absolute antibodies

Supplementary Table 4: antibodies for CUT & Tag

Target	Species	ID	Supplier
MPP8	Rabbit	16796-1-AP	Proteintech
TASOR	Rabbit	HPA017142	Atlas Antibodies
PPHLN1	Rabbit	HPA038902	Atlas Antibodies
H3K9me3	Rabbit	ab8898	Abcam
H3K27ac	Rabbit	ab4729	Abcam
IgG	Rabbit	12-370	Millipore

Supplementary Table 5: Software used

Software	Version
CellQuest	unknown
FACSDiva	unknown
cbcl2fastq	2.19
Adobe Suite	6.4.0.361
ChatGPT	GPT-4
FlowJo	10.10.0
GraphPad Prism	10.4.0
ImageJ	1.53
Mendeley Reference Manager	1.19.8
Microsoft Office	365
Microsoft	10.0.19045
LightCycler Software	Unknown
anaconda3	5.2.0
bedops	2.4.35
Bedtools	2.28.0
blast+	2.6.0
bowtie2	2.3.1
git	2.39.2
miniconda	4.12.0
perl	5.26.0
python	2.7.15
R	3.4.3
salmon	0.13.1
samtools	0.1.19
star	2.6.1a
trimgalore	0.4.5-1
