# Supplementary material for: Epigenetic lockdown of type I interferon sensing and signalling in human pluripotent cells
Source: Nat Commun. 2026 Jun 16;17:7660. doi: 10.1038/s41467-026-74147-0 (PMC13434735; doi:10.1038/s41467-026-74147-0)
Supplement: Supplementary file 2 [file 41467_2026_74147_MOESM2_ESM.pdf]

nature portfolio

Corresponding author(s): Helen M Rowe

Last updated by author(s): 21/04/2026

Reporting Summary

Nature Portfolio wishes to improve the reproducibility of the work that we publish. This form provides structure for consistency and transparency in reporting. For further information on Nature Portfolio policies, see our Editorial Policies and the Editorial Policy Checklist.

Statistics

For all statistical analyses, confirm that the following items are present in the figure legend, table legend, main text, or Methods section.

n/a	Confirmed	
☐	☒	The exact sample size (*n*) for each experimental group/condition, given as a discrete number and unit of measurement
☐	☒	A statement on whether measurements were taken from distinct samples or whether the same sample was measured repeatedly
☐	☒	The statistical test(s) used AND whether they are one- or two-sided<br>*Only common tests should be described solely by name; describe more complex techniques in the Methods section.*
☒	☐	A description of all covariates tested
☐	☒	A description of any assumptions or corrections, such as tests of normality and adjustment for multiple comparisons
☐	☒	A full description of the statistical parameters including central tendency (e.g. means) or other basic estimates (e.g. regression coefficient) AND variation (e.g. standard deviation) or associated estimates of uncertainty (e.g. confidence intervals)
☐	☒	For null hypothesis testing, the test statistic (e.g. *F*, *t*, *r*) with confidence intervals, effect sizes, degrees of freedom and *P* value noted<br>*Give P values as exact values whenever suitable.*
☒	☐	For Bayesian analysis, information on the choice of priors and Markov chain Monte Carlo settings
☒	☐	For hierarchical and complex designs, identification of the appropriate level for tests and full reporting of outcomes
☒	☐	Estimates of effect sizes (e.g. Cohen's *d*, Pearson's *r*), indicating how they were calculated

Our web collection on statistics for biologists contains articles on many of the points above.

Software and code

Policy information about availability of computer code

Data collection	BD CellQuest or BD FACSDiva for flow cytometry data acquisition.<br>Samples were sequenced on a NextSeq 3000 instrument (Illumina Cambridge, Chesterford, UK).<br>Illumina' s bcl2fastq Conversion Software v2.19 was used to demultiplex data.
Data analysis	Name Supplier Version<br>Adobe suite Adobe 6.4.0.361<br>ChatGPT OpenAI GPT-4<br>FlowJo Software BD Biosciences 10.10.0<br>GraphPad Prism Software GraphPad Software 10.4.0<br>ImageJ Software NIH 1.53<br>Mendeley Reference Manager Elsevier 1.19.8<br>Microsoft Office Microsoft 365<br>Microsoft Windows Microsoft 10.0.19045<br>Roche LIGHTCYCLER® Software Roche Unknown<br>anaconda3 5.2.0<br>bedops 2.4.35<br>Bedtools 2.28.0<br>blast+ 2.6.0<br>bowtie2 2.3.1<br>git 2.39.2<br>miniconda 4.12.0

perl 5.26.0
python 2.7.15
R 3.4.3
salmon 0.13.1
samtools 0.1.19
star 2.6.1a
trimgalore 0.4.5-1
Custom enrichment scripts are available on github under "RoweLab".
https://github.com/RoweLab/TE-Enrichment/tree/main

For manuscripts utilizing custom algorithms or software that are central to the research but not yet described in published literature, software must be made available to editors and reviewers. We strongly encourage code deposition in a community repository (e.g. GitHub). See the Nature Portfolio guidelines for submitting code & software for further information.

Data

Policy information about availability of data

All manuscripts must include a data availability statement. This statement should provide the following information, where applicable:

- Accession codes, unique identifiers, or web links for publicly available datasets
- A description of any restrictions on data availability
- For clinical datasets or third party data, please ensure that the statement adheres to our policy

GSE318471 and GSE318510

Research involving human participants, their data, or biological material

Policy information about studies with human participants or human data. See also policy information about sex, gender (identity/presentation), and sexual orientation and race, ethnicity and racism.

Reporting on sex and gender	Experiments were performed on cultured cells from human donors from biobanks, with 1 male and 1 female donor based on self reporting.
Reporting on race, ethnicity, or other socially relevant groupings	N/A
Population characteristics	N/A
Recruitment	N/A
Ethics oversight	N/A

Note that full information on the approval of the study protocol must also be provided in the manuscript.

Field-specific reporting

Please select the one below that is the best fit for your research. If you are not sure, read the appropriate sections before making your selection.

☒ Life sciences ☐ Behavioural & social sciences ☐ Ecological, evolutionary & environmental sciences

For a reference copy of the document with all sections, see nature.com/documents/nr-reporting-summary-flat.pdf

Life sciences study design

All studies must disclose on these points even when the disclosure is negative.

Sample size	Sample sizes were chosen to reach 3 independent biological replicates to reach biological robustness and are stated in figure legends.
Data exclusions	Data were not excluded from the analyses.
Replication	All experiments were repeated at least three times to ensure reproducibility, with the exception of CUT & Tag where 2 replicates were used.
Randomization	Allocation of all samples into experimental groups was random and groups were treated the same.
Blinding	The investigators who carried out the experiments were not blinded to the groups and we did not consider blinding necessary because all groups were treated the same way.

Reporting for specific materials, systems and methods

We require information from authors about some types of materials, experimental systems and methods used in many studies. Here, indicate whether each material, system or method listed is relevant to your study. If you are not sure if a list item applies to your research, read the appropriate section before selecting a response.

Materials & experimental systems

n/a	Involved in the study
☐	☒ Antibodies
☐	☒ Eukaryotic cell lines
☒	☐ Palaeontology and archaeology
☒	☐ Animals and other organisms
☒	☐ Clinical data
☒	☐ Dual use research of concern
☒	☐ Plants

Methods

n/a	Involved in the study
☒	☐ ChIP-seq
☐	☒ Flow cytometry
☒	☐ MRI-based neuroimaging

Antibodies

Antibodies used

Western blotting antibodies
Target Species Dilution Supplier ID
ACTIN-HRP rabbit 1/10000 Cell Signalling Technology 5125
GAPDH-HRP rabbit 1/10000 Cell Signalling Technology 8884T
IFIT1 rabbit 1/1000 Cell Signalling Technology 14769S
IFIT1 rabbit 1/1000 Cell Signalling Technology 92633
IFITM3 rabbit 1/1000 Cell Signalling Technology 59212
IRF3 rabbit 1/1000 Cell Signalling Technology 4302
MDA5 rabbit Jan-00 Cell Signalling Technology 5321S
Mouse HRP horse 1/10000 Cell Signalling Technology 7076S
MPP8 rabbit 1/1000 Proteintech 16796-1-AP
MX1 rabbit 1/1000 Cell Signalling Technology 37849S
Oct4 rabbit Jan-00 Cell Signalling Technology 2840
PCNA rabbit 1/1000 Cell Signalling Technology 13110S
Rabbit HRP goat 1/10000 Cell Signalling Technology 7074S
RIG-I rabbit Jan-00 Cell Signalling Technology 3743S
Tubulin HRP rabbit 1/10000 Abcam ab21058

Antibodies for microscopy / ICS
Target Species ID Supplier
Oct4 Mouse 653703 Biolegend
SSEA4 Mouse 46-8843-42 Thermo
PAX6 Rabbit PSI-5581 Biolegend
NANOG Mouse ab80892 Abcam

Antibodies for CUT & RUN / CUT & Tag
Target Species ID Supplier
MPP8 Rabbit 16796-1-AP Proteintech
H3K9me3 Rabbit ab8898 Abcam
H3K27ac Rabbit ab4729 Abcam
IgG Rabbit 12-370 Millipore

Validation

Antibodies were validated to react with the human versions of the proteins by the manufacturer. Antibodies were further validated to detect proteins of the expected band sizes by Western blot.

Eukaryotic cell lines

Policy information about cell lines and Sex and Gender in Research

Cell line source(s)

HEK293 cells were from ATCC. HeLa cells
were obtained from Caetano Reis e Sousa (originally from ATCC). Commercial THP-1 cell lines containing an integrated Lucia luciferase IFIT-2 reporter was purchased from Invivogen.
Female wibj2 cells were purchased from the HIPSCI consortium
Male WTC11 cells were a kind gift from Elena Bochukova.

Authentication

Morphology test on microscope and growth monitoring from prior experience with working with these cell types. Knockout cell lines were further verified by Western blot and functional assays as detailed in the methods and figures.

Mycoplasma contamination

Regular mycoplasma testing was performed.

Commonly misidentified lines
(See ICLAC register)

N/A

Plants

Seed stocks: N/A

Novel plant genotypes: N/A

Authentication: N/A

Flow Cytometry

Plots

Confirm that:

☐ The axis labels state the marker and fluorochrome used (e.g. CD4-FITC).

☒ The axis scales are clearly visible. Include numbers along axes only for bottom left plot of group (a 'group' is an analysis of identical markers).

☒ All plots are contour plots with outliers or pseudocolor plots.

☒ A numerical value for number of cells or percentage (with statistics) is provided.

Methodology

Sample preparation: Cells were harvested with trypsin or accutase in media followed by pelleting at 400 g, washing with PBS, re-pelleting, and resuspension in FACS buffer (PBS, 1% FCS, 5mM EDTA). For intracellular and extracellular immunostaining cells were aliquoted into 5 x 10^5 cells per condition. Surface antigens were stained in 100µl FACS Buffer with SSEA4-PerCP710 antibody on ice for 20 minutes before washing twice in FACS buffer. Cells were then resuspended in 100 µl of 1x Fix buffer (made by diluting Fix 1:4 in Fix diluent) before incubation in the dark at RT for 20-60 mins. Antibodies used for ICS are shown earlier in this document.
Cells were washed in 1ml Perm buffer before being resuspended in 100µl antibody-perm mix and incubated in the dark at RT for 20-60 mins. Excess antibody was washed off with two washes in 1ml Perm and 2 washes in 1ml FACS buffer. Cells were resuspended in 0.2-1ml FACS buffer before being run on a BD LSRII. 30,000 samples were acquired per sample and data were analysed using FlowJo (Tree Star). For compensation, single-stained cell populations were used.

Instrument: Becton Dickinson LSRII

Software: FlowJo (Tree Star).

Cell population abundance: Cell populations were not sorted on a cell sorter

Gating strategy: Flow cytometry data were analysed using FlowJo software. Forward Scatter (FSC-A) vs. Side Scatter (SSC-A) was used to gate based on size and granularity to exclude debris and cell aggregates. To exclude doublets and clumps, cells were plotted on FSC-A vs. FSC-H, single cells were gated by selecting events where FSC-A and FSC-H values were linear and proportional. Gates were set on the using untreated/unstained cells to establish the negative population.

☒ Tick this box to confirm that a figure exemplifying the gating strategy is provided in the Supplementary Information.
