# Supplementary material for: Epigenetic lockdown of type I interferon sensing and signalling in human pluripotent cells
Source: Nat Commun. 2026 Jun 16;17:7660. doi: 10.1038/s41467-026-74147-0 (PMC13434735; doi:10.1038/s41467-026-74147-0)
Supplement: Supplementary file 3 [file 41467_2026_74147_MOESM3_ESM.pdf]

nature portfolio

Peer Review File

Epigenetic lockdown of type I interferon sensing and signalling in human pluripotent cells.

Corresponding Author: Professor Helen Rowe

This file contains all reviewer reports in order by version, followed by all author rebuttals in order by version.

Version 0:

Reviewer comments:

Reviewer #1

(Remarks to the Author)
In Epigenetic lockdown of type I interferon sensing and signaling in human pluripotent cells prepared by Holt et al. propose MPP8/HUSH as a key regulator of ISG and LINE1 repression during early development. Without enhanced repression of ISG and L1s, iPSCs fail to retain their pluripotent profile and are incapable of differentiation. Intriguingly, pluripotent cells resist ISG expression even upon viral infection or intracellular ISG signaling. In addition to repressing ISG transcription, young L1 subfamilies that most recently emerged in the human genome are those specifically repressed by MPP8 and HUSH. The authors employ the iPSC line WIBJ2 and MPP8 shRNA to explore their hypothesis that MPP8 represses ISGs during early development. The results presented by the authors indeed suggest this hypothesis to be true. However, these results are preliminary and the conclusions reached by the authors are wide-ranging relative to the data presented and therefore require significant additions to justify the claims made. Additionally, numerous aspects of the manuscript are confusing. Specific critiques are listed below.

1. In the abstract, the last sentence states pluripotent cells ‘represent a ground state of immune evasion that cancer cells may evolve towards through increasing expression of MPP8.’ The authors do not explore anything related to cancer within their results section making the inclusion of this sentence confusing.
2. The results rely heavily on dsRNA sensing from RIG-I cytosolic sensing. In theory, these dsRNAs arise from L1 elements. However, it has been shown in multiple publications that L1s readily form cDNAs or DNA/RNA hybrids that are recognized by cytosolic sensors such as cGAS. How do the authors know cGAS is not involved in the upregulation of IFN signaling upon MPP8 shRNA treatment? They show in figure 1l that cGAS is upregulated and cGAS activity is known to be linked to IRF transcription factor activation. cGAS is also known to bind dsRNA. Given the central role of cytosolic sensing in the results shown, cGAMP activity and potential STING activation (indicated by phosphorylated STING) needs to be evaluated as a potential source of inflammation. If cGAS is not important in this process, they need to show this using a cGAS inhibitor or shRNA.
3. The results rely heavily upon RT-qPCR. The authors employ GAPDH as their reference gene in this analysis. However, as the authors note in line 220, there are many copies of each L1 subfamily within the human genome. Therefore, it is important to use a reference gene that also has multiple copies within the human genome, such as a ribosomal subunit gene. This is significantly more reflective than GAPDH.
4. The RT-qPCR results presented by the authors have multiple inconsistencies. They do not use a consistent scale for the y-axis (some are in scientific notation others are presented with the control normalized to 1). Additionally, some bar graphs show each individual point (figure 3d and e) but most do not show any of the points even as they have extreme error bars (figure 2c, 2g, 3b). This is extremely confusing and makes the reader suspicious of the results presented.
5. The schematic shown in figure 1d shows western blotting and IC staining employed a n of only 1. This needs to be increased to at least 3. Additionally, the ICS image shown is way too low of a magnification to assess the fluorescent markers the authors stained for.
6. The authors do not list what antibodies they use in their methods.
7. More evidence of L1 activity in these cells need to be included. Cell staining for ORF1p, dsRNA, and DNA/RNA hybrids should be included. This will also reinforce if dsRNA is at the center of the cytosolic sensing.
8. To prove L1 dsRNA is indeed driving the outcome observed, cells chould be treated with RNase to rescue the inflammatory signaling.
9. L1 expression is crucial to the results reported by the authors. Therefore, NRTI treatment of cells to prevent L1 activity and rescue inflammatory signaling would strengthen the results.

Reviewer #2

(Remarks to the Author)
This work by Holt et al. investigates the role of the HUSH complex in regulating the expression of genes involved in the IFN response in pluripotent stem cells. This is a timely study, as over the past 15–20 years, the field has shown that stem cells exhibit a distinct immune organization compared to differentiated cells. This includes a limited responsiveness of the IFN pathway combined with the constitutive expression of a subset of ISGs at steady state. The mechanisms governing gene regulation behind stem cell-specific immunity remain largely uncharacterized. I therefore believe this work provides highly valuable insights. The manuscript is clear, well-written, and the interpretation of the data is sound. I have a few points, listed below, that should be addressed by the authors. Provided these are satisfactorily tackled, I recommend this work for publication.

1. The Rice lab demonstrated that one stem cell-specific defense mechanism is the constitutive expression of certain ISGs. The ISGs monitored by the authors, such as IFIT and Mx family members, do not belong to this group. Could the authors comment on this? Do they observe any ISGs expressed at steady state in their dataset? If so, are these not targeted by the HUSH complex?

2. As shown by the authors, NPCs respond to IFN, unlike iPS cells. What is the role of MPP8 in this context? It would strongly support the authors' claims if depletion of MPP8 in NPCs does not affect ISG levels.

3. The interpretation of Fig. 1 (line 172) anticipates subsequent data. At this stage, it is unclear whether HUSH directly regulates the expression of these genes or influences the IFN pathway, which in turn controls PRR expression. I suggest using more cautious language (e.g., "suggests" rather than definitive statements).

4. Western blots should be quantified throughout the manuscript (e.g., Fig. 1F, 1H).

5. In Fig. 2A, please indicate standard deviations or display biological replicates.

Reviewer #3

(Remarks to the Author)
This is an impressive paper documenting the involvement of HUSH (1 and 2) in inactivating the IFN pathway in iPSC cells. The data lead to the suggestion of a direct action of HUSH2 at IRF1, IRF2, and ISRE motifs to repress ISG expression. This is just one part of the redundant machinery to switch off ligands, sensors, and effectors of the IFN pathway. The findings are important in defining the mechanisms of suppressing ISGs in stem cells.

The introduction provides a detailed and clear summary of the present understanding of the iPSC state with respect to IFN responses, and of the complex literature on the topic.

KD of MPP8 by shRNA (even a modest KD or 84%) resulted in increased expression of the ISGs IFIT1 and 2, and of 143 more genes that included ISGs. RIG-I and MDA5 were also increased by the KD. A subset of LINE-1 elements, mainly young ones, were also upregulated by KD. (The PCR readouts do not report individual elements but instead pool together very close family members.) The selectivity for LINE induction over random genes was not overwhelming, a weakness. The upregulated TEs were not located at ISGs. (What is meant by TEs "in" particular genes (ll. 240-244) needs clarification. Calling them "intragenic" would be helpful.) Importantly, the increased ISG expression upon KD in iPSC cells did not involve the classical IFN pathway (unlike in HFFs or HEK293 cells). The suggestion is that this is mediated by TASOR2. The KD did not induce differentiation.

The binding profile for MPP8 was determined by CUT&Tag, and did not well correlate with H3K9me3, but did show peaks at TSS of MPP8-regulated ISGs. I find the data on H3K4me3 to be confusing -- not sure how it should be interpreted. The profiles on ISGs and non-ISGs were distinguishable. The non-ISG genes that were upregulated contained TEs that could have been the drivers of expression upon the KD. Motifs potentially responsible were identified.

The proposal is that "the ISGs … identified as bound by MPP8 are likely TASOR2 targets while non-ISGs are likely TASOR targets." This is plausible and attractive but should probably be presented as a proposal.

The paper is very well-written and the data are compelling. A limitation is that there does not seem to be a direct demonstration of TASOR2 binding at ISGs. Perhaps reagents for this are not available.

For clarity, line 452, "including the RNA sensors RIG-I and MDA5", should probably read "including the genes for the RNA sensors RIG-I and MDA5".

Version 1:

Reviewer comments:

Reviewer #1

(Remarks to the Author)
The authors have addressed most but not all the concerns. An important conclusion is that ISG signaling is suppressed in IPSCs by HUSH complex. What happens if that inhibition is perturbed is somewhat secondary, but here the authors failed to fully address the concern. It is an easy experiment to treat the cells with NRTIs to test whether ISG expression would be ameliorated. Without this test there is not enough evidence to rule out a DNA sensing pathway.

Reviewer #2

(Remarks to the Author)
The authors have satisfactorily addressed all my questions. I recommend this work for publication.

Enzo Poirier

Reviewer #3

(Remarks to the Author)
I feel that the authors have done a commendable job responding to my comments, and indeed those of the other reviewers. The data support the role of HUSH in maintaining pluripotency of their cells.

Open Access This Peer Review File is licensed under a Creative Commons Attribution 4.0 International License, which permits use, sharing, adaptation, distribution and reproduction in any medium or format, as long as you give appropriate credit to the original author(s) and the source, provide a link to the Creative Commons license, and indicate if changes were made.
In cases where reviewers are anonymous, credit should be given to 'Anonymous Referee' and the source.
The images or other third party material in this Peer Review File are included in the article's Creative Commons license, unless indicated otherwise in a credit line to the material. If material is not included in the article's Creative Commons license and your intended use is not permitted by statutory regulation or exceeds the permitted use, you will need to obtain permission directly from the copyright holder.
To view a copy of this license, visit https://creativecommons.org/licenses/by/4.0/

Response to Reviewers

Reviewer #1 (Remarks to the Author):

In Epigenetic lockdown of type I interferon sensing and signaling in human pluripotent cells prepared by Holt et al. propose MPP8/HUSH as a key regulator of ISG and LINE1 repression during early development. Without enhanced repression of ISG and L1s, iPSCs fail to retain their pluripotent profile and are incapable of differentiation. Intriguingly, pluripotent cells resist ISG expression even upon viral infection or intracellular ISG signaling. In addition to repressing ISG transcription, young L1 subfamilies that most recently emerged in the human genome are those specifically repressed by MPP8 and HUSH. The authors employ the iPSC line WIBJ2 and MPP8 shRNA to explore their hypothesis that MPP8 represses ISGs during early development. The results presented by the authors indeed suggest this hypothesis to be true. However, these results are preliminary and the conclusions reached by the authors are wide-ranging relative to the data presented and therefore require significant additions to justify the claims made. Additionally, numerous aspects of the manuscript are confusing. Specific critiques are listed below.

1. In the abstract, the last sentence states pluripotent cells 'represent a ground state of immune evasion that cancer cells may evolve towards through increasing expression of MPP8.' The authors do not explore anything related to cancer within their results section making the inclusion of this sentence confusing.

We have removed this reference to cancer and agree that we do not explore anything related to cancer in this manuscript.

2. The results rely heavily on dsRNA sensing from RIG-I cytosolic sensing. In theory, these dsRNAs arise from L1 elements. However, it has been shown in multiple publications that L1s readily form cDNAs or DNA/RNA hybrids that are recognized by cytosolic sensors such as cGAS. How do the authors know cGAS is not involved in the upregulation of IFN signaling upon MPP8 shRNA treatment? They show in figure 1I that cGAS is upregulated and cGAS activity is known to be linked to IRF transcription factor activation. cGAS is also known to bind dsRNA. Given the central role of cytosolic sensing in the results shown, cGAMP activity and potential STING activation (indicated by phosphorylated STING) needs to be evaluated as a potential source of inflammation. If cGAS is not important in this process, they need to show this using a cGAS inhibitor or shRNA.

While we agree that L1 nucleic acids are implicated in cytosolic sensing in differentiated cells [1,2], our results show that in pluripotent stem cells, interferon-stimulated genes (ISGs) are regulated by direct binding of the HUSH complex to these genes leading to their transcriptional silencing (Figure 4). We show that:

i) cytosolic sensing is impaired in pluripotent cells because immunostimulatory ligands (i.e. poly (I:C), 3p-hpRNA) do not induce an interferon response (Figure 1).

ii). The upregulation of interferon-stimulated genes observed upon MPP8-depletion is independent of both the type I interferon receptor and JAK-STAT signaling in pluripotent

cells but not in HEK293 cells, as demonstrated using a type I interferon neutralising antibody or using the JAK inhibitor Ruxolitinib, respectively (Figure 3D, E). Consistent with these results, direct addition of IFN-beta to iPSCs does not trigger an IFN response, whereas IFN-beta treatment in NPCs results in the induction of ISGs (Figure 1B).

We now further support these findings to answer the reviewer's comment about cGAS by providing new data to show that treatment of iPSCs with G3-YSD, a cGAS agonist, doesn't lead to the induction of ISGs in contrast to THP-1 cells (used in parallel as a positive control) in which G3-YSD resulted in the upregulation of an ISRE reporter (see new Supplementary Figure 1). We also show that STING mRNA is not expressed in iPSCs using our RNA-sequencing data (Figure 1L, Supplementary Figure 1D) and we have now done Western blots to confirm that STING is not expressed at the protein level in iPSCs either, although it is readily expressed in THP-1s and primary fibroblasts (New Supplementary Figure 3). These results show that DNA sensing, like RNA sensing, is impaired in iPSCs and that the upregulation of ISGs that is observed upon MPP8 depletion is caused by the direct de-repression of these genes by the HUSH complex.

These new results are shown below and in the manuscript.

Supplementary Figure 1

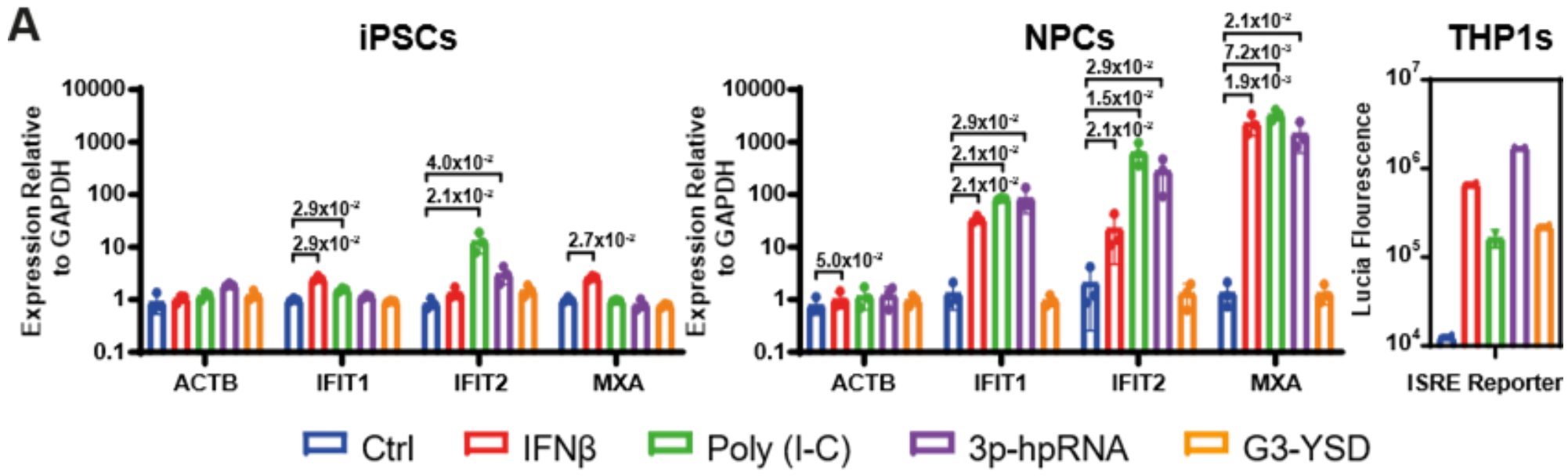

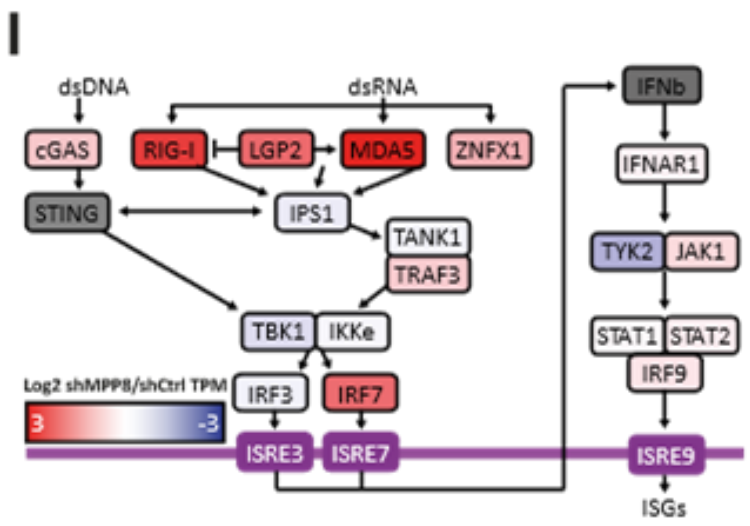

Supplementary Figure 3

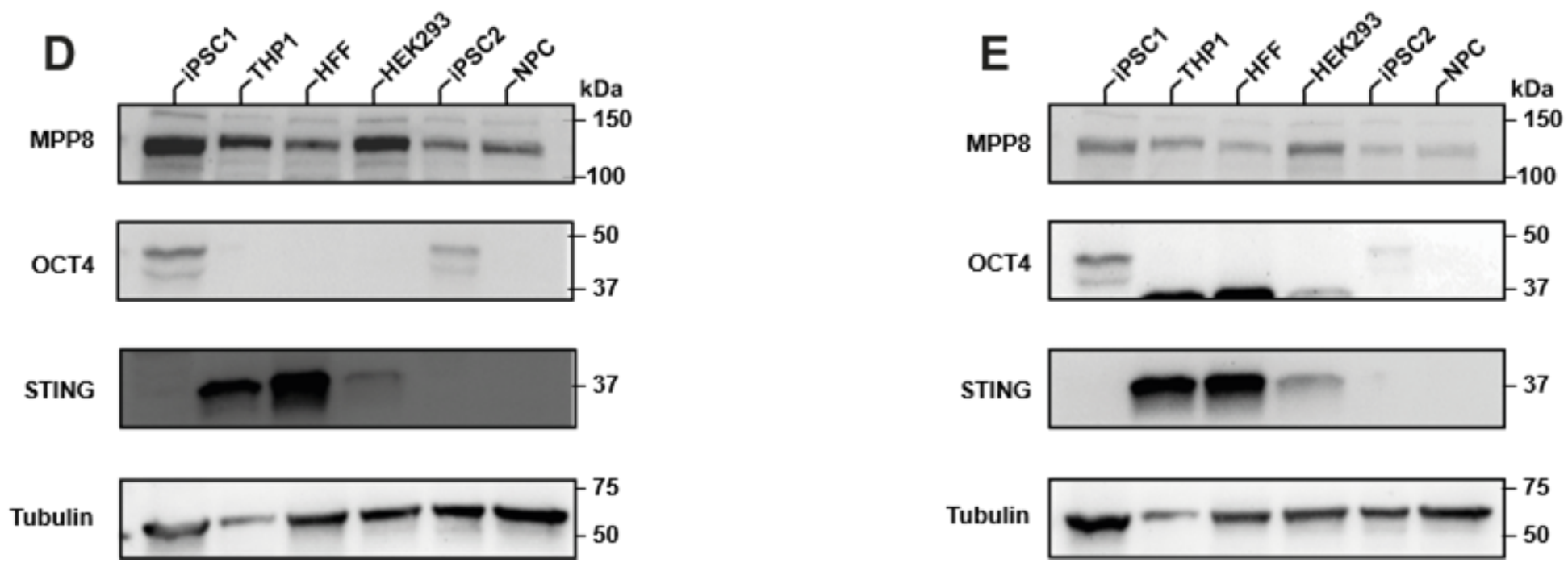

3. The results rely heavily upon RT-qPCR. The authors employ GAPDH as their reference gene in this analysis. However, as the authors note in line 220, there are many copies of each L1 subfamily within the human genome. Therefore, it is important to use a reference gene that also has multiple copies within the human genome, such as a ribosomal subunit gene. This is significantly more reflective than GAPDH.

All RT-qPCRs were performed with two different reference genes (GAPDH and beta ACTIN) throughout the manuscript. As suggested by the reviewer, the 16s rRNA housekeeping gene was now used as an additional housekeeping gene to normalise the relative expression of LINE-1 subfamilies in Figure 2C (see below):

Figure 2

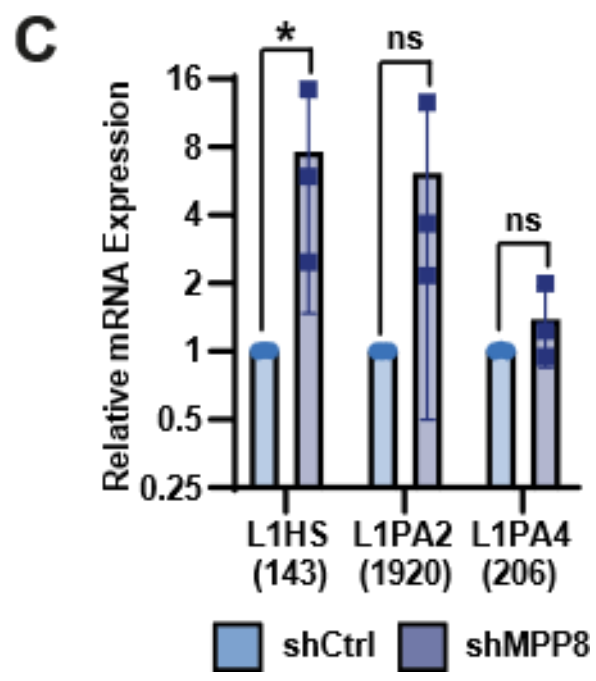

4. The RT-qPCR results presented by the authors have multiple inconsistencies. They do not use a consistent scale for the y-axis (some are in scientific notation others are presented with the control normalized to 1). Additionally, some bar graphs show each individual point (figure 3d and e) but most do not show any of the points even as they have extreme error bars (figure 2c, 2g, 3b). This is extremely confusing and makes the reader suspicious of the results presented.

RT-qPCR plots have been amended to always use log scales for consistency and to include individual data points.

5. The schematic shown in figure 1d shows western blotting and IC staining employed a n of only 1. This needs to be increased to at least 3. Additionally, the ICS image shown is way too low of a magnification to assess the fluorescent markers the authors stained for.

Verification of pluripotency markers: We have now performed Western blots on triplicate shMPP8 experiments with one replicate displayed in the main figures (R1) and the remaining blots are shown in new Supplementary Figure 3 and below. We have also repeated the western blots used to examine protein expression in each cell type. This now represents n=3 independent experiments for all western blots except: ISG15 following shMPP8 (n=4) and STING by cell type (n=2).

Protein expression following shMPP8

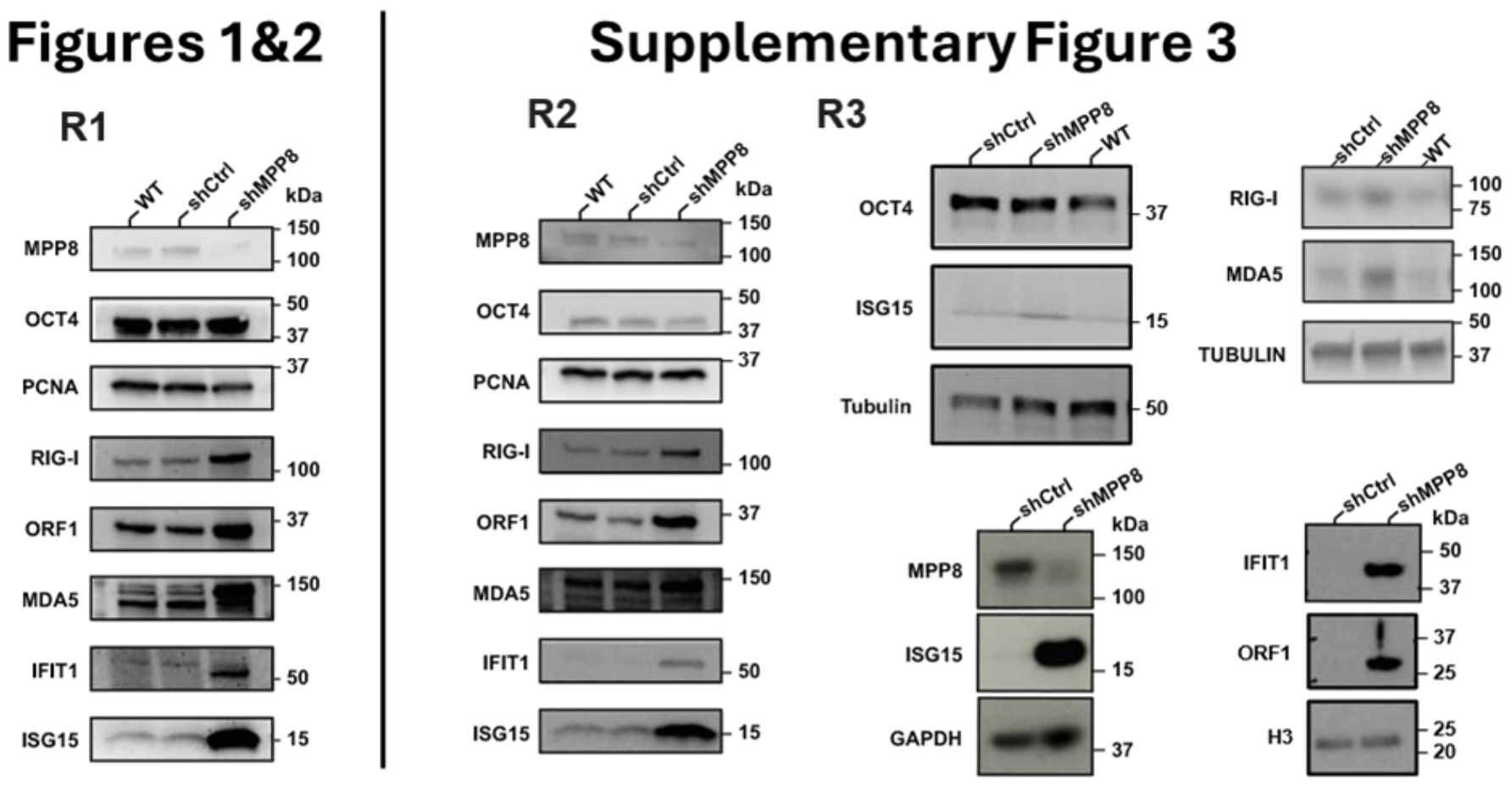

Protein expression by cell type

Supplementary Figure 3

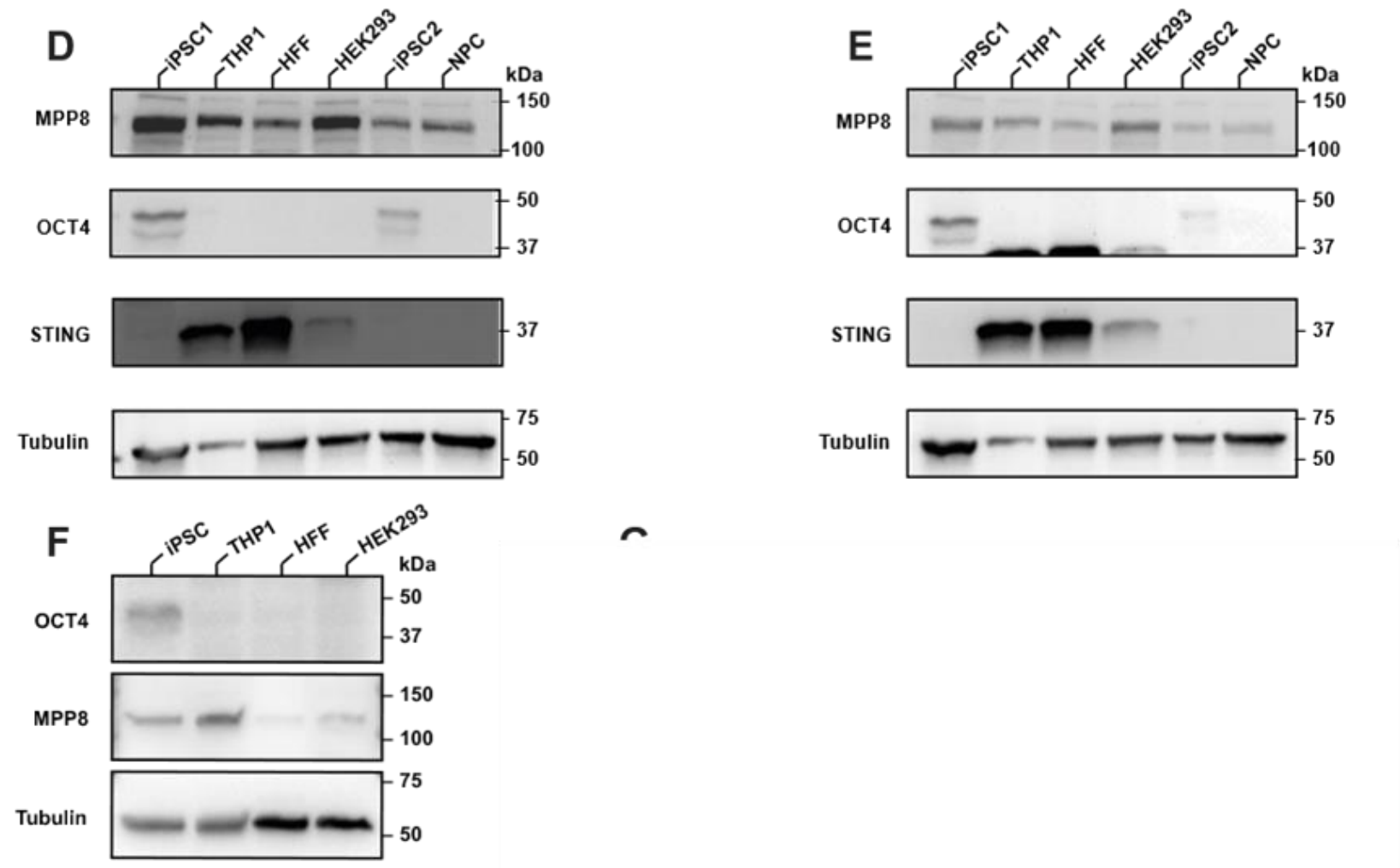

The ICS-microscopy (see below) has now been moved into Supplementary Figure 2, and we have now re-done further microscopy replicates. The summary data is shown for PAX6 expression in NPCs at an increased magnification (now N=4). The ICS-flow cytometry data is now moved into Supplementary Figure 1B, showing two replicates of iPSC 1 stained for OCT4/SSEA4.
In addition, RT-qPCR validation of OCT4 and NANOG expression is now shown for triplicate experiments in both our iPSC lines alongside NPCs and HEK293s as controls (Supplementary Figure 1C).

In sum, to validate the identity of our cells, we performed multiple independent assays across biological replicates as follows:

For NPCs, we conducted PAX6 immunofluorescence microscopy (n=4), OCT4/NANOG RT-qPCR (n=3), and OCT4 western blots (n=2).

For iPSC 1, we performed intracellular flow cytometry for OCT4 and NANOG (n=2), OCT4/NANOG qRT-qPCR (n=3), and OCT4 western blot analysis (n=3).

For iPSC 2, we carried out intracellular staining and microscopy for OCT4 and NANOG (n=1), OCT4/NANOG qRT-qPCR (n=3), and OCT4 western blot analysis (n=2).

Supplementary Figure 2

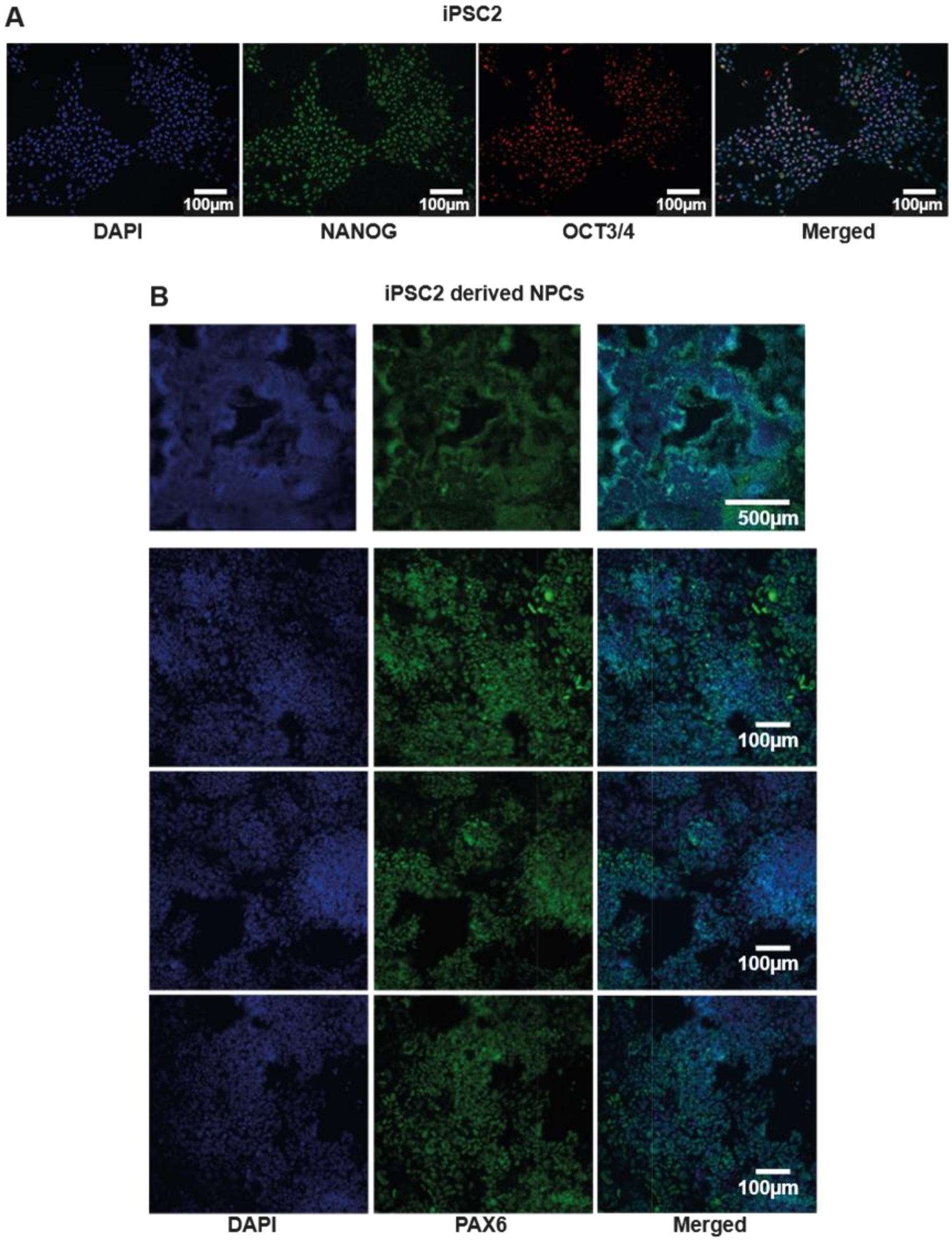

Supplementary Figure 1

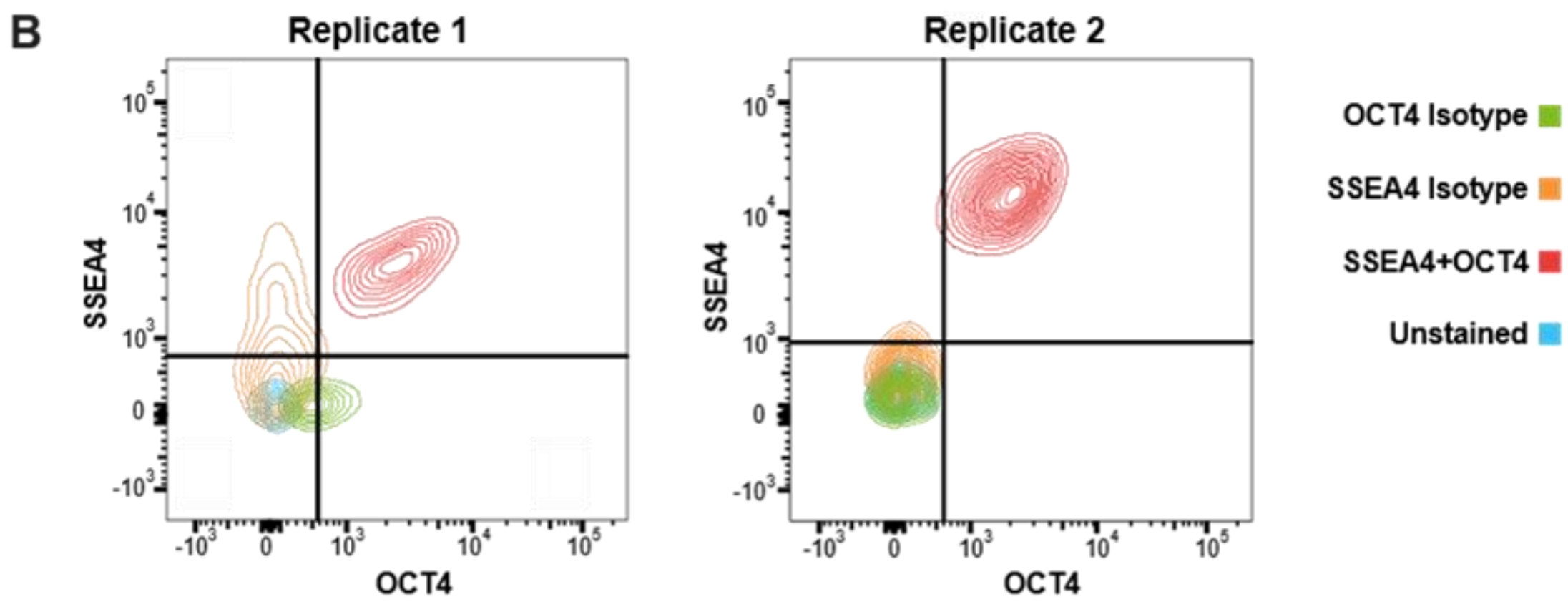

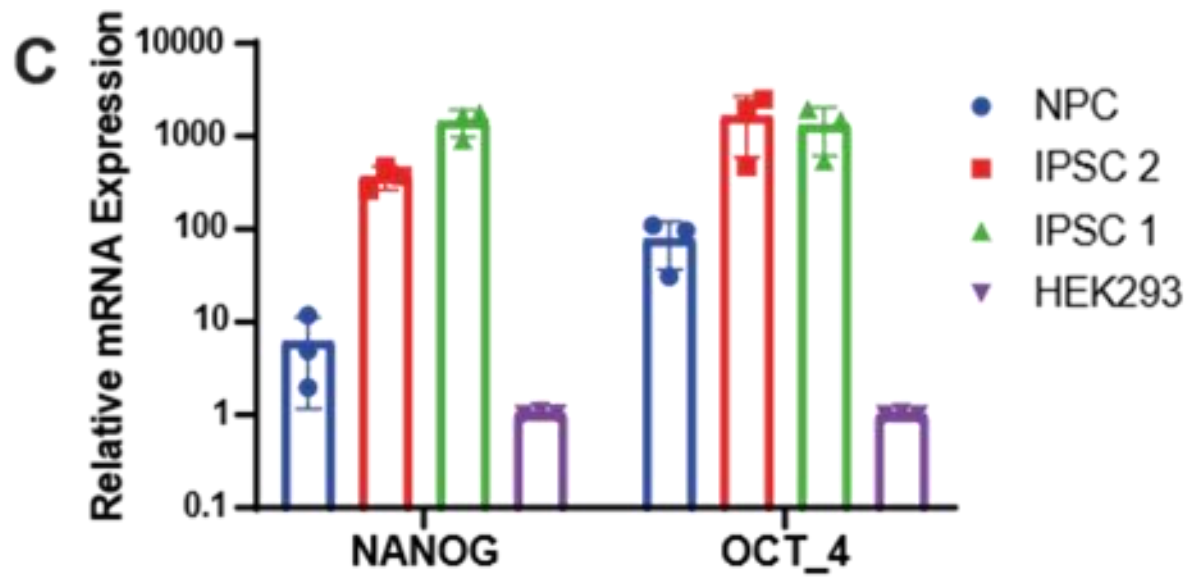

6. The authors do not list what antibodies they use in their methods.

A list of antibodies has been added to the source data file, and the Methods section has been updated to indicate this.

7. More evidence of L1 activity in these cells need to be included. Cell staining for ORF1p, dsRNA, and DNA/RNA hybrids should be included. This will also reinforce if dsRNA is at the center of the cytosolic sensing.

We have now verified that cytosolic sensing (RNA sensing and DNA sensing) is impaired in iPSCs (see answer to the reviewer's question 2 above). We have also now verified that LINE-1 RNA and L1 ORF1 protein are both increased in expression in iPSCs following MPP8-depletion (Figure 2 and Supplementary Figure 3) and young LINE-1 elements are direct binding targets of MPP8 in iPSCs (Figure 4). However, dsRNA sensing is not occurring in iPSCs, and interferon response genes are repressed directly by HUSH complex binding and silencing (Figure 4).

8. To prove L1 dsRNA is indeed driving the outcome observed, cells chould be treated with RNase to rescue the inflammatory signaling.

See answer to reviewer question 7, above.

9. L1 expression is crucial to the results reported by the authors. Therefore, NRTI treatment of cells to prevent L1 activity and rescue inflammatory signaling would strengthen the results.

Our data shows that ISG expression in MPP8-depleted iPSCs is independent of cytosolic sensing (see answer to reviewer question 2, above).

Reviewer #2 (Remarks to the Author):

This work by Holt et al. investigates the role of the HUSH complex in regulating the expression of genes involved in the IFN response in pluripotent stem cells. This is a timely study, as over the past 15–20 years, the field has shown that stem cells exhibit a distinct immune organization compared to differentiated cells. This includes a limited responsiveness of the IFN pathway combined with the constitutive expression of a subset of ISGs at steady state. The mechanisms governing gene regulation behind stem cell-specific immunity remain largely uncharacterized. I therefore believe this work provides highly valuable insights. The manuscript is clear, well-written, and the interpretation of the data is sound. I have a few points, listed below, that should be addressed by the authors. Provided these are satisfactorily tackled, I recommend this work for publication.

1. The Rice lab demonstrated that one stem cell-specific defense mechanism is the constitutive expression of certain ISGs. The ISGs monitored by the authors, such as IFIT and Mx family members, do not belong to this group. Could the authors comment on this? Do they observe any ISGs expressed at steady state in their dataset? If so, are these not targeted by the HUSH complex?

We have now addressed this question by analysing intrinsically expressed ISGs (those identified by the Rice lab and a custom list of intrinsically expressed ISGs, which we have curated from our own data using the same approach as the Rice lab [(3)]). We cross-referenced Transcripts Per Million (TPM) values derived from our RNA-seq data to determine whether the ISGs identified by the Rice lab are expressed in our cells (Supplementary Figure 4A) and found that the majority are expressed in our iPSCs. We then applied the same methodology described by Wu et al. to identify intrinsic ISGs and generated a personalised list of intrinsically expressed ISGs in our iPSCs, which shows a substantial overlap with their list (Supplementary Figure 4 B-C). Notably, intrinsically expressed ISGs showed minimal changes in expression following MPP8 depletion, while MPP8 loss caused an upregulation of genes with low baseline expression (Supplementary Figure 4 B, D) This is now presented as new Supplementary Figure 4 and below.

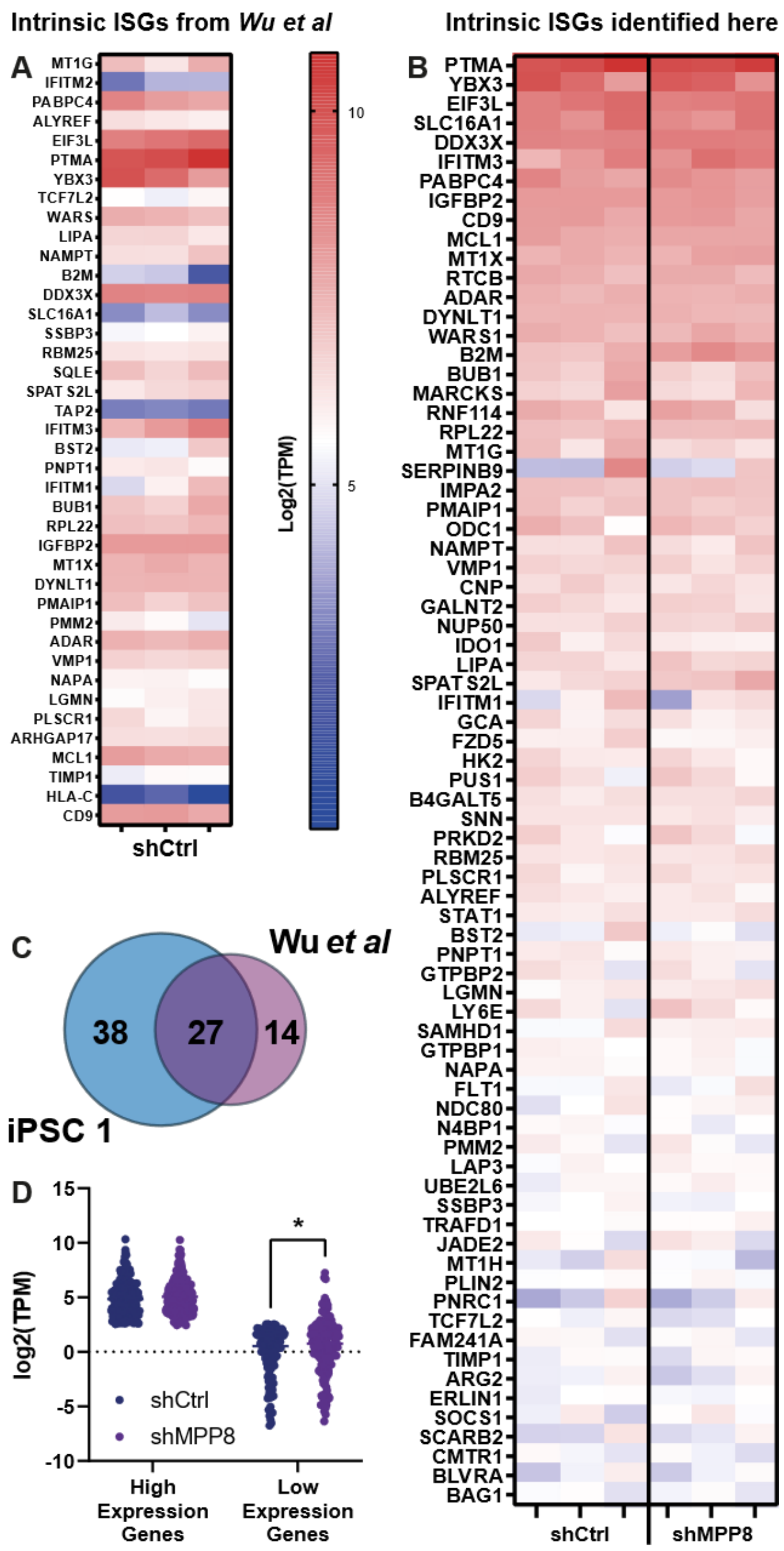

2. As shown by the authors, NPCs respond to IFN, unlike iPS cells. What is the role of MPP8 in this context? It would strongly support the authors' claims if depletion of MPP8 in NPCs does not affect ISG levels.

We addressed this question and the question of whether ISG upregulation in NPCs relies on IFNAR signalling by performing MPP8-depletion in NPCs with and without Ruxolitinib (an inhibitor of JAK-STAT signalling). However, although we were able to differentiate iPSCs to NPCs, we were unable to transduce NPCs with lentiviral vectors to deplete MPP8 as effectively as we could in iPSCs, despite multiple attempts. Therefore, while we did not observe ISG induction in this NPC setting, this might be due to the MPP8-depletion not being as efficient in NPCs as iPSCs. We are including this Figure as a

reviewer figure only (below) and follow-up questions about the regulation of the type I interferon system in NPCs or neurons is beyond the scope of this study.

However, based on our previous data obtained in HEK 293 cells (Figure 3D, E) and THP-1 cells [(2)], we anticipate that depletion of MPP8 in NPCs, which have a robust type I IFN response to Poly (I-C), will induce ISG expression in a manner dependent on nucleic acid sensing and IFNAR.

A

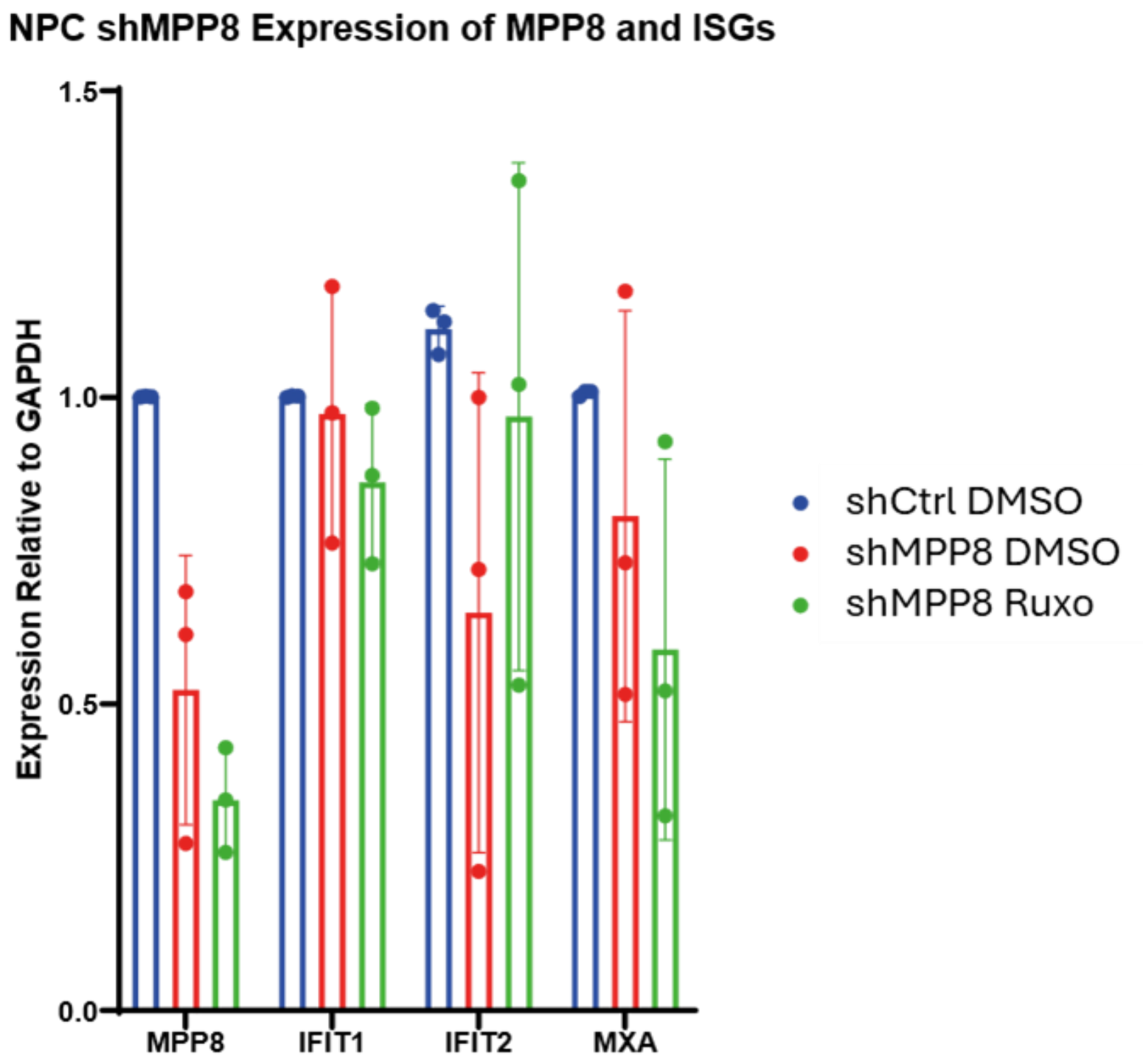

B

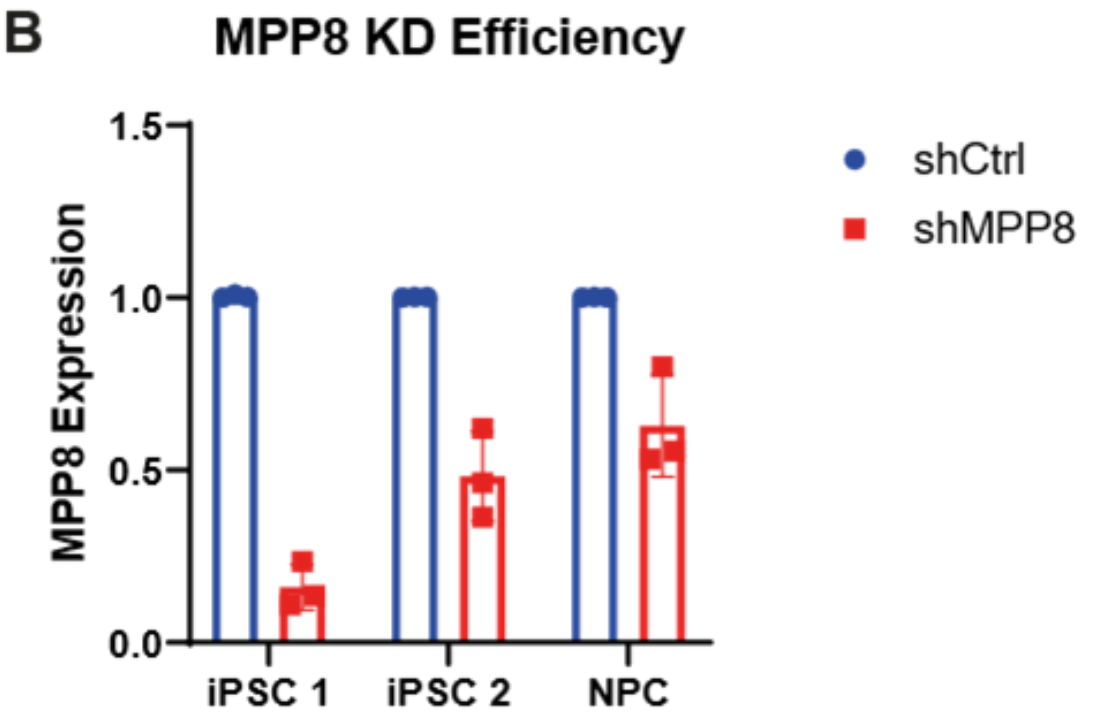

3. The interpretation of Fig. 1 (line 172) anticipates subsequent data. At this stage, it is unclear whether HUSH directly regulates the expression of these genes or influences the IFN pathway, which in turn controls PRR expression. I suggest using more cautious language (e.g., "suggests" rather than definitive statements).

We have amended interpretations of Figure 1 accordingly.

4. Western blots should be quantified throughout the manuscript (e.g., Fig. 1F, 1H).

Western blots have now been quantified, and this information is contained in new Supplementary Figure 3 and below.

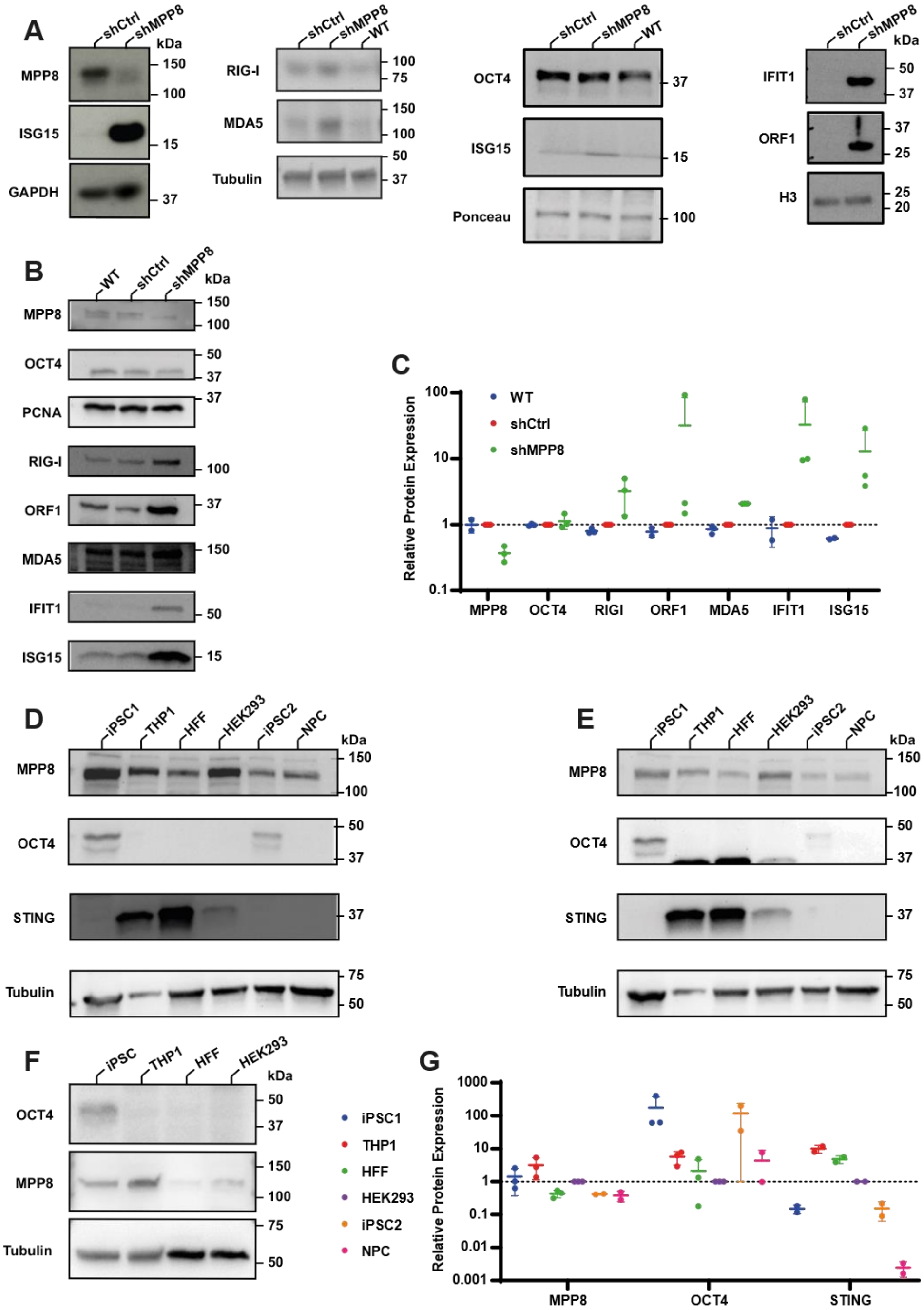

5. In Fig. 2A, please indicate standard deviations or display biological replicates.

This has now been done.

Reviewer #3 (Remarks to the Author):

This is an impressive paper documenting the involvement of HUSH (1 and 2) in inactivating the IFN pathway in iPSC cells. The data lead to the suggestion of a direct action of HUSH2 at IRF1, IRF2, and ISRE motifs to repress ISG expression. This is just one part of the redundant machinery to switch off ligands, sensors, and effectors of the IFN pathway. The findings are important in defining the mechanisms of suppressing ISGs in stem cells.
The introduction provides a detailed and clear summary of the present understanding of the iPSC state with respect to IFN responses, and of the complex literature on the topic.

1.KD of MPP8 by shRNA (even a modest KD or 84%) resulted in increased expression of the ISGs IFIT1 and 2, and of 143 more genes that included ISGs. RIG-I and MDA5 were also increased by the KD. A subset of LINE-1 elements, mainly young ones, were also upregulated by KD. (The PCR readouts do not report individual elements but instead pool together very close family members.)

We have now added clarification of this point into the text in lines 224-227.

2.The selectivity for LINE induction over random genes was not overwhelming, a weakness.

We agree that the level of L1 de-repression is modest here, but it is notable that there is an increase in L1 RNA and L1 ORF1 protein in MPP8-depleted iPSCs and young L1 elements are direct binding targets of MPP8. It is also important to note that cytosolic sensing is impaired in these cells (see reviewer 1, answer to comment 2) and interferon response genes are directly regulated by HUSH complex binding to them and silencing them. We have now used coverage plots derived from CUT & Tag data to overlap repetitive regions of interest and determine enrichment of MPP8, H3K9me3 and H3K27ac over these regions and the surrounding 2kb of genome. This showed that MPP8 binding to L1HS-L1PA3 elements has a peak of 10-25 reads per 10 million mapped reads, while for non-LINE TEs, the peak ranges from 0.2-1.5 reads, indicating that MPP8 binding is enriched at young LINE1s over other HUSH regulated TEs in iPSCs (see below):

Supplementary Figure 5

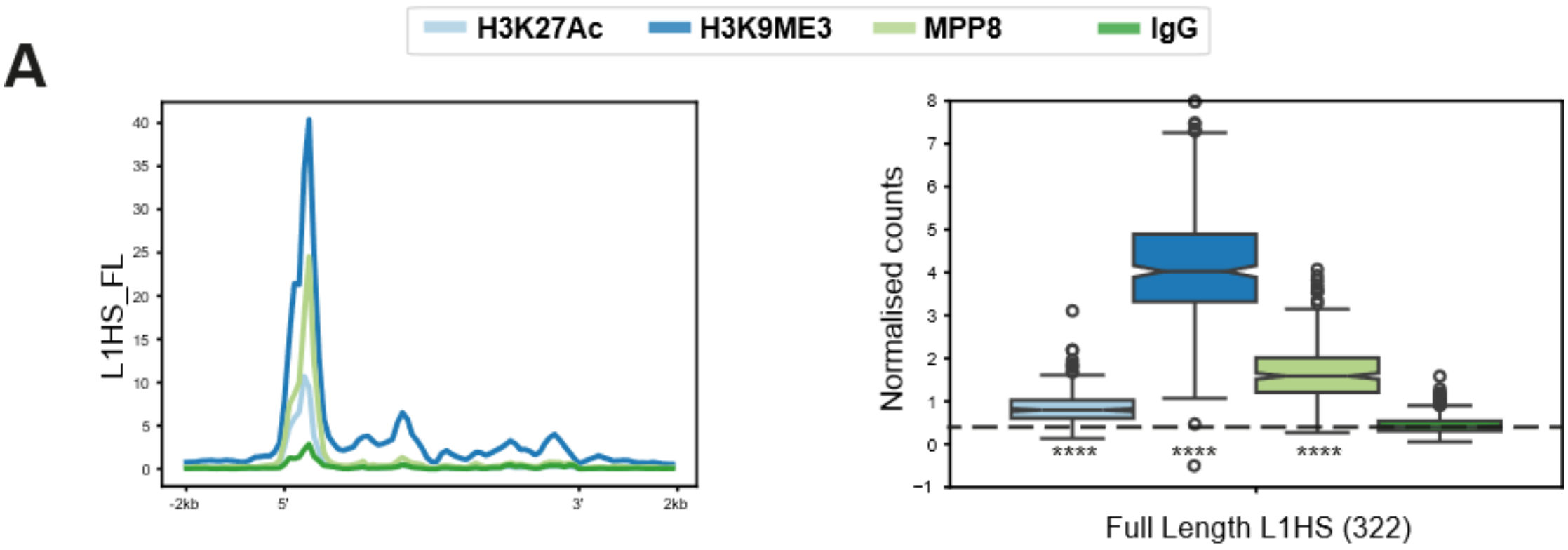

Supplementary Figure 6

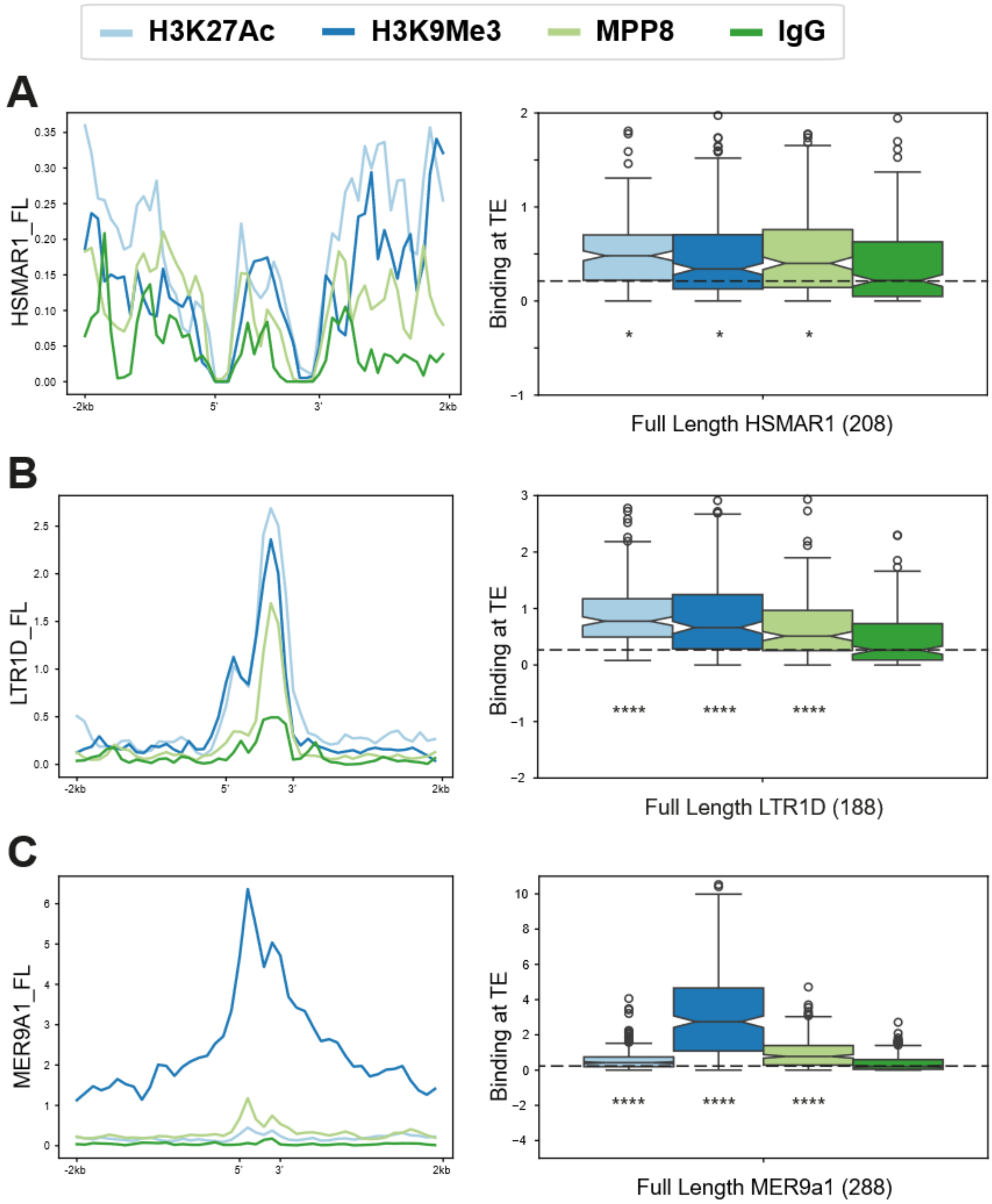

3.The upregulated TEs were not located at ISGs. (What is meant by TEs “in” particular genes (ll. 240-244) needs clarification. Calling them “intragenic” would be helpful.) Importantly, the increased ISG expression upon KD in iPSC cells did not involve the classical IFN pathway (unlike in HFFs or HEK293 cells). The suggestion is that this is mediated by TASOR2. The KD did not induce differentiation.

These TEs have been renamed to “intragenic” throughout the text.

4. The binding profile for MPP8 was determined by CUT&Tag, and did not well correlate with H3K9me3, but did show peaks at TSS of MPP8-regulated ISGs. I find the data on H3K4me3 to be confusing -- not sure how it should be interpreted. The profiles on ISGs and non-ISGs were distinguishable. The non-ISG genes that were upregulated contained TEs that could have been the drivers of expression upon the KD. Motifs potentially responsible were identified.

We have added in further explanation of the implications of the H3K27ac binding data in lines 349-350.

5. The proposal is that “the ISGs … identified as bound by MPP8 are likely TASOR2 targets while non-ISGs are likely TASOR targets.” This is plausible and attractive but should probably be presented as a proposal.

We have altered the text to reflect this.

6. The paper is very well-written and the data are compelling. A limitation is that there does not seem to be a direct demonstration of TASOR2 binding at ISGs. Perhaps reagents for this are not available.

The antibodies required to perform TASOR2 investigations were not available at the time that this study was conducted. However, we now discuss TASOR2 and HUSH2 in the discussion and suggest that following up a detailed dissection of the role of HUSH2 in iPSCs and early development could be a follow-up study.

7. For clarity, line 452, “including the RNA sensors RIG-I and MDA5”, should probably read “including the genes for the RNA sensors RIG-I and MDA5”.

Now clarified (line 455).

References

1. De Cecco M, Ito T, Petrashen AP, Elias AE, Skvir NJ, Criscione SW, Caligiana A, Brocculi G, Adney EM, Boeke JD, Le O, Beausejour C, Ambati J, Ambati K, Simon M, Seluanov A, Gorbunova V, Slagboom PE, Helfand SL, Neretti N, Sedivy JM. 2019. L1 drives IFN in senescent cells and promotes age-associated inflammation. Nature 566:73-78.

2. Tunbak H, Enriquez-Gasca R, Tie CHC, Gould PA, Mlcochova P, Gupta RK, Fernandes L, Holt J, van der Veen AG, Giampazolias E, Burns KH, Maillard PV, Rowe HM. 2020. The HUSH complex is a gatekeeper of type I interferon through epigenetic regulation of LINE-1s. Nat Commun 11:5387.

3. Wu X, Thi VLD, Huang Y, Billerbeck E, Saha D, Hoffmann H-H, Wang Y, Silva LAV, Sarbanes S, Sun T, Andrus L, Yu Y, Quirk C, Li M, MacDonald MR, Schneider WM, An X, Rosenberg BR, M Rice CM. 2018. Intrinsic Immunity Shapes Viral Resistance of Stem Cells. Cell 172, 423-438.e25.

Response to the reviewers' comments.

Reviewer #1 (Remarks to the Author):

The authors have addressed most but not all the concerns. An important conclusion is that ISG signaling is suppressed in IPSCs by HUSH complex. What happens if that inhibition is perturbed is somewhat secondary, but here the authors failed to fully address the concern. It is an easy experiment to treat the cells with NRTIs to test whether ISG expression would be ameliorated. Without this test there is not enough evidence to rule out a DNA sensing pathway.

We thank the reviewer for taking the time to review our work. We have previously shown that LINE-1 nucleic acid sensing occurs in differentiated cells and our study here highlights that pluripotent cells are different, representing an immune privileged state in early human development. Specifically, we show that DNA sensing is prohibited in pluripotent cells because (1) there is a lack of an ISG response to the cGAS agonist G3YSD (Supplementary Figure 1A) and (2) there is an absence of STING expression in these cells as measured by RNA-sequencing (Supplementary Figure 1D) and Western blot (Supplementary Figures 3D and 3E). Furthermore, in pluripotent cells, interferon response genes are directly suppressed through MPP8. Altogether, the triple epigenetic lockdown of ligands, sensors and effectors of the interferon pathway prevents unwanted type I interferon responses to retroelement expression in early development.

Reviewer #2 (Remarks to the Author):

The authors have satisfactorily addressed all my questions. I recommend this work for publication.

Enzo Poirier

Thank you for taking the time to review our work, we are glad to have satisfactorily addressed your concerns.

Reviewer #3 (Remarks to the Author):

I feel that the authors have done a commendable job responding to my comments, and indeed those of the other reviewers. The data support the role of HUSH in maintaining pluripotency of their cells.

Thank you for taking the time to review our work, we are glad to have satisfactorily addressed your concerns.
