# Supplementary material for: Epigenetic lockdown of type I interferon sensing and signalling in human pluripotent cells
Source: Nat Commun. 2026 Jun 16;17:7660. doi: 10.1038/s41467-026-74147-0 (PMC13434735; doi:10.1038/s41467-026-74147-0)

Replicate 1

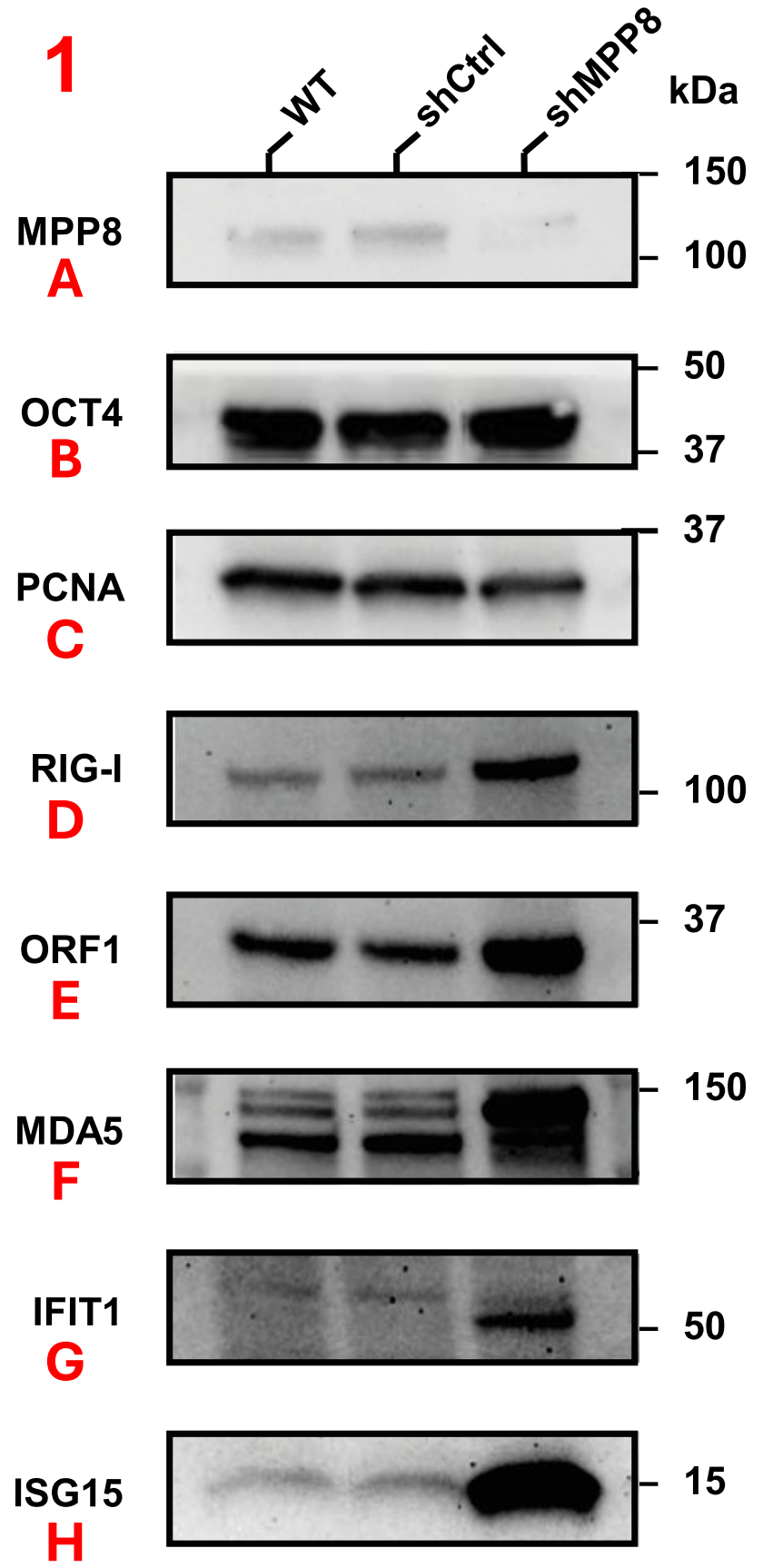

Replicate 2

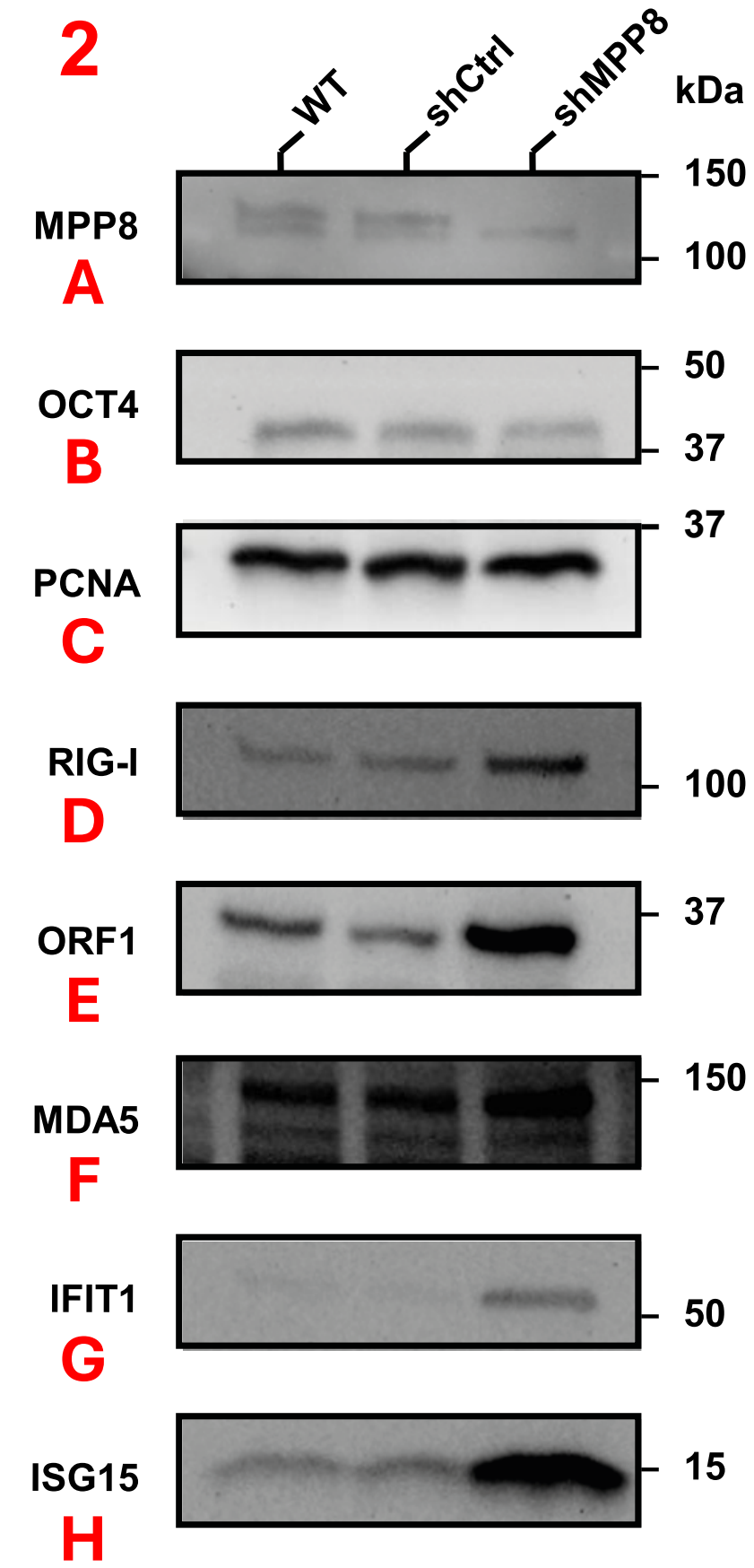

Replicate 3

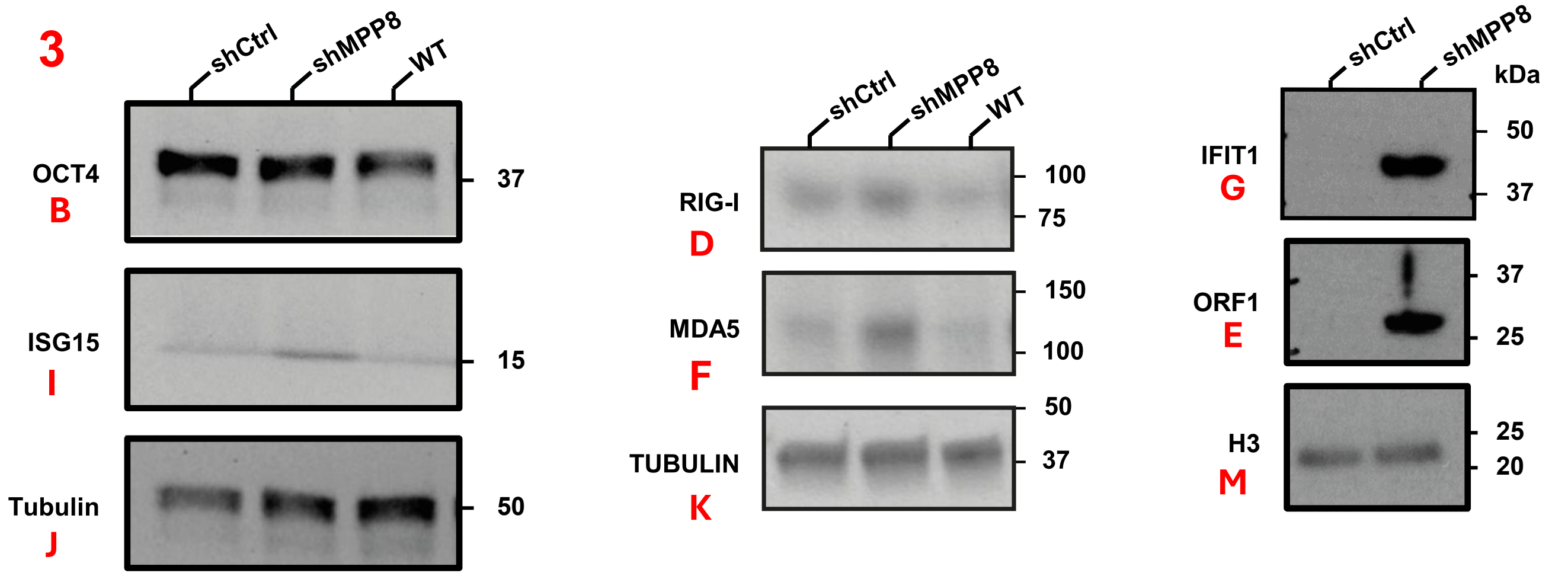

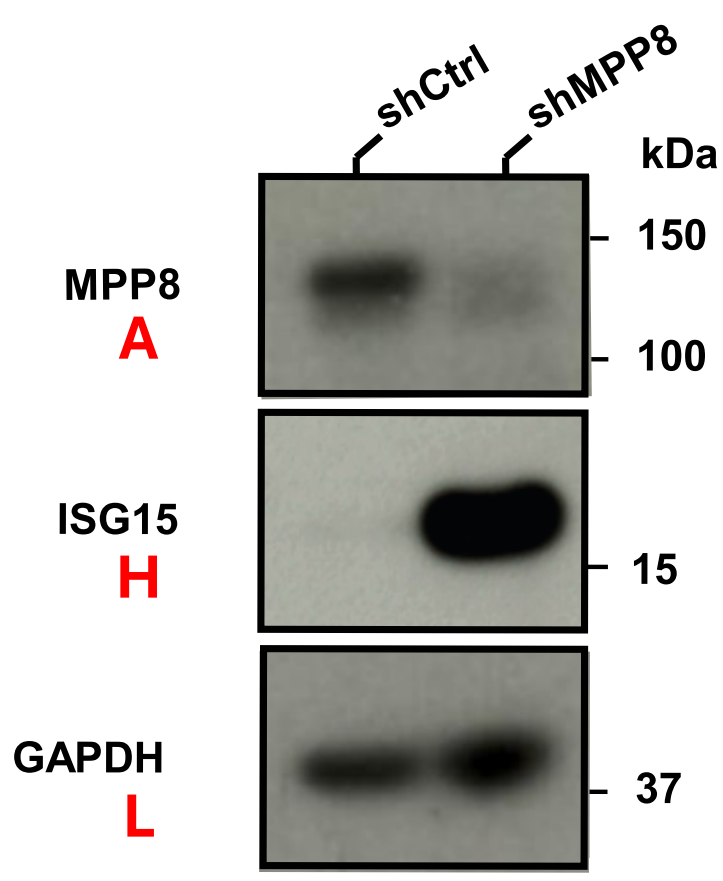

N.B. The numbers in the top right of the membranes indicate the blot number in each batch of western blots and are not consistent with replicate numbers for experiments

* These blots were performed twice per replicate but only one is shown in the manuscript, both are shown here. Red = included, Orange = not included

NI = Blots not included in the main or supplementary figures of this paper

Top = RIGI (2D)
Bottom = ORF1 (NI)

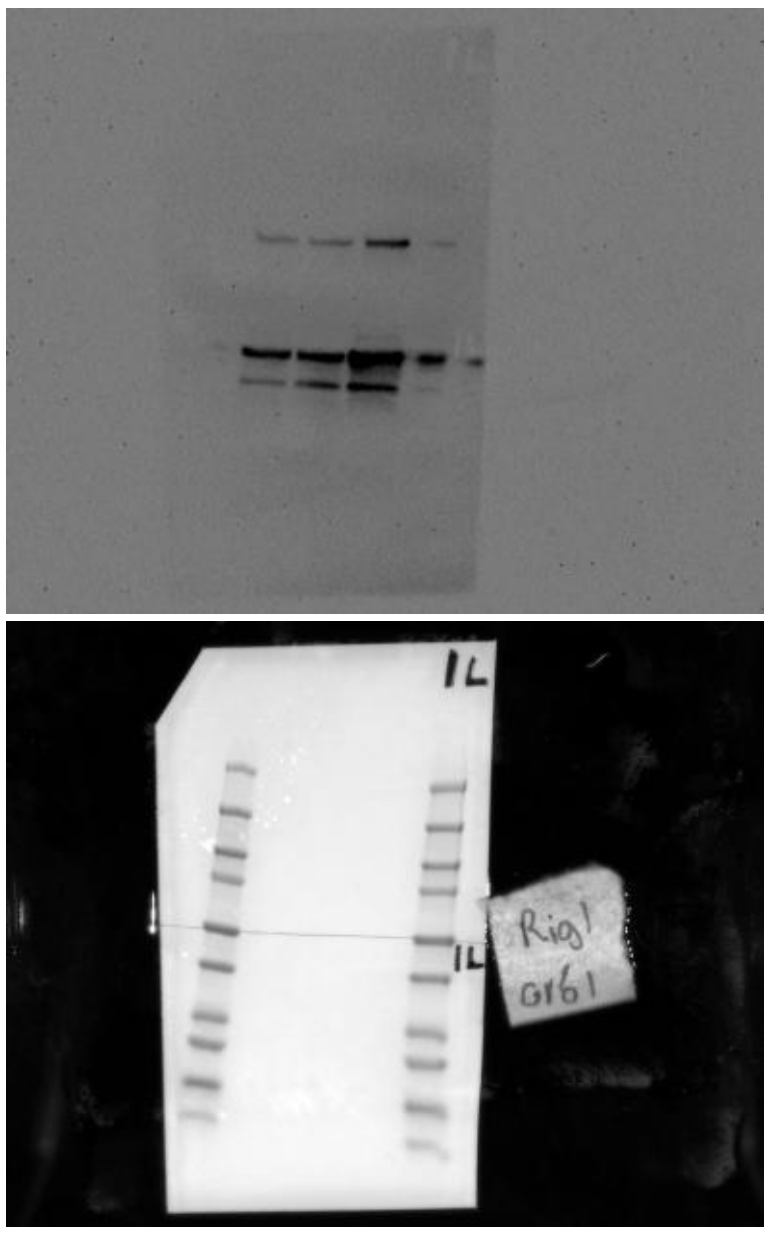

Top = MDA5 (2F)
Bottom = OCT4 (2B)

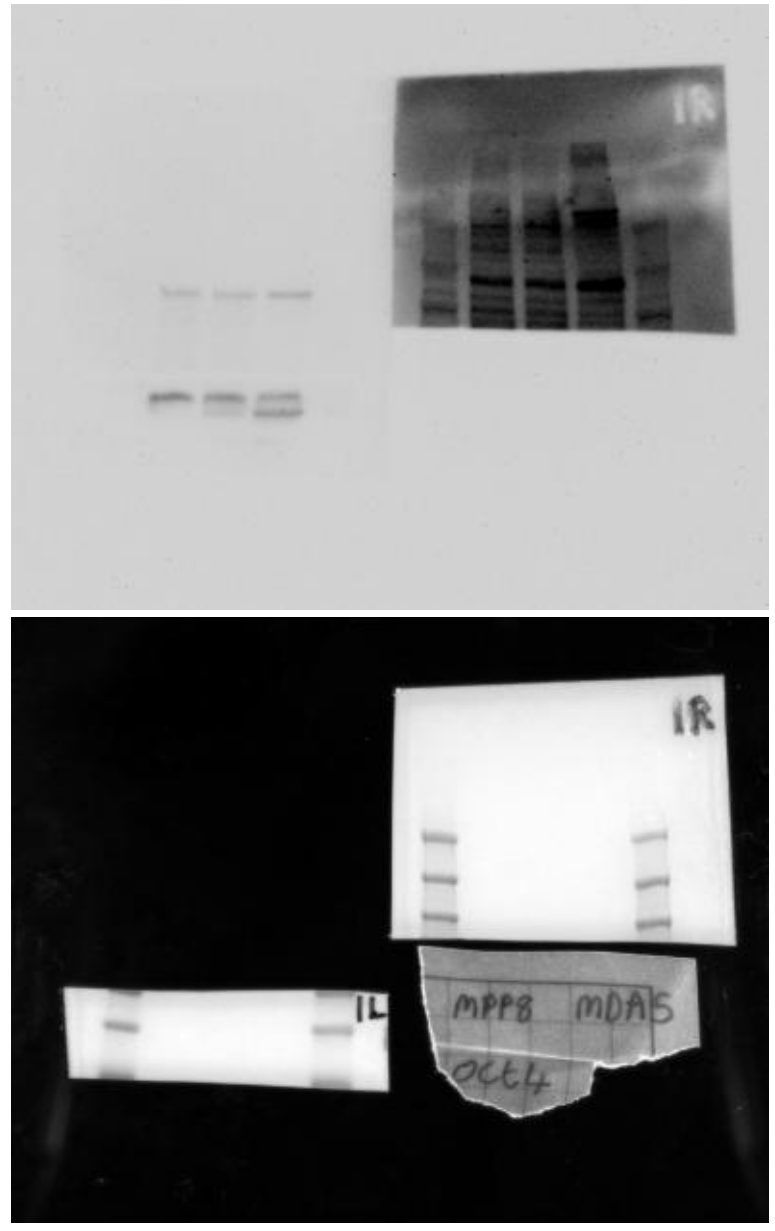

Top = MDA5 (NI)
Middle = IFIT1 (2G)
Bottom = ISG15 (2H)

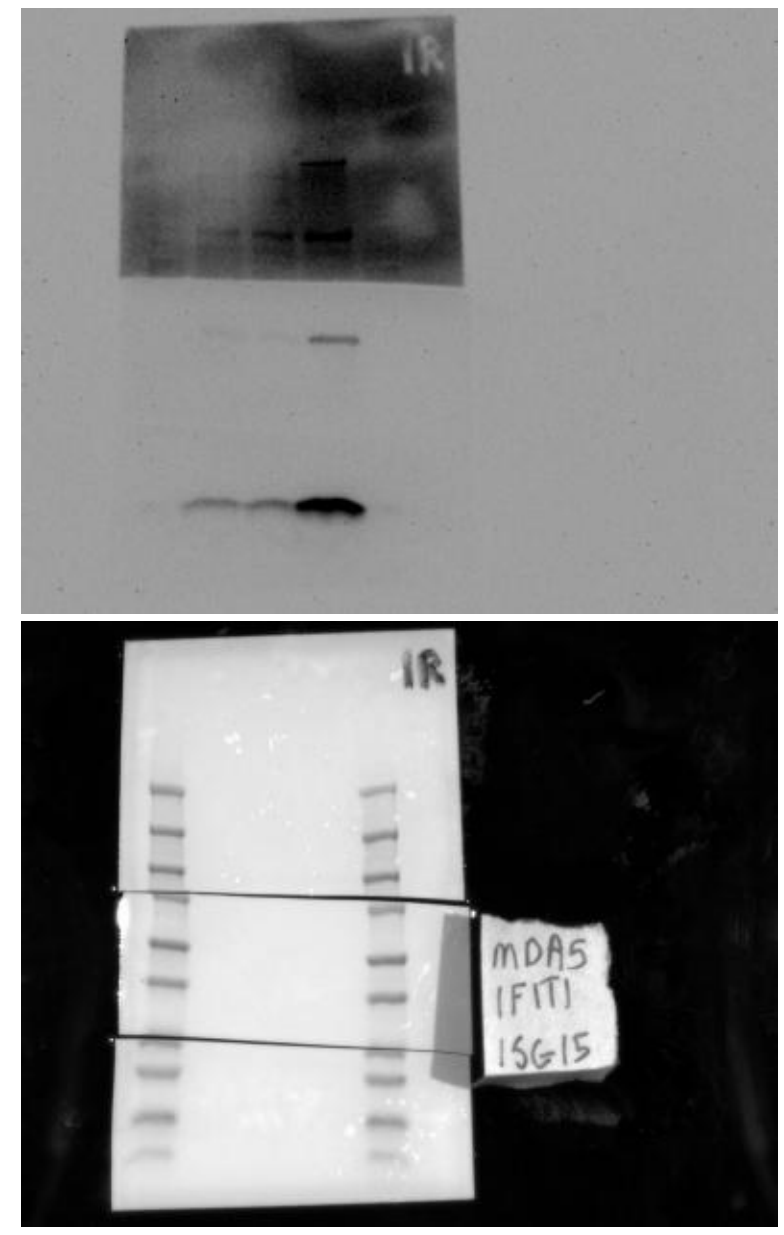

Top = RIGI (1D)
Bottom = ORF1 (NI)

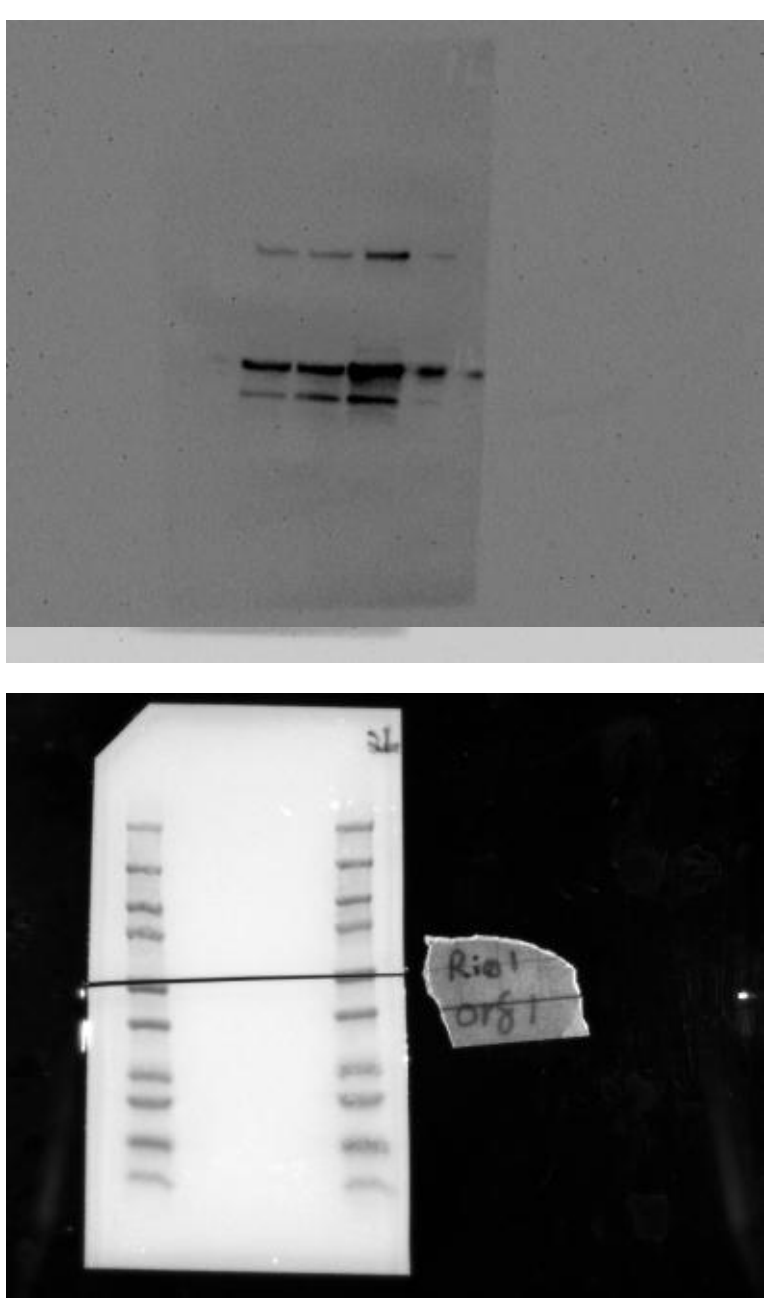

MPP8 (2A)

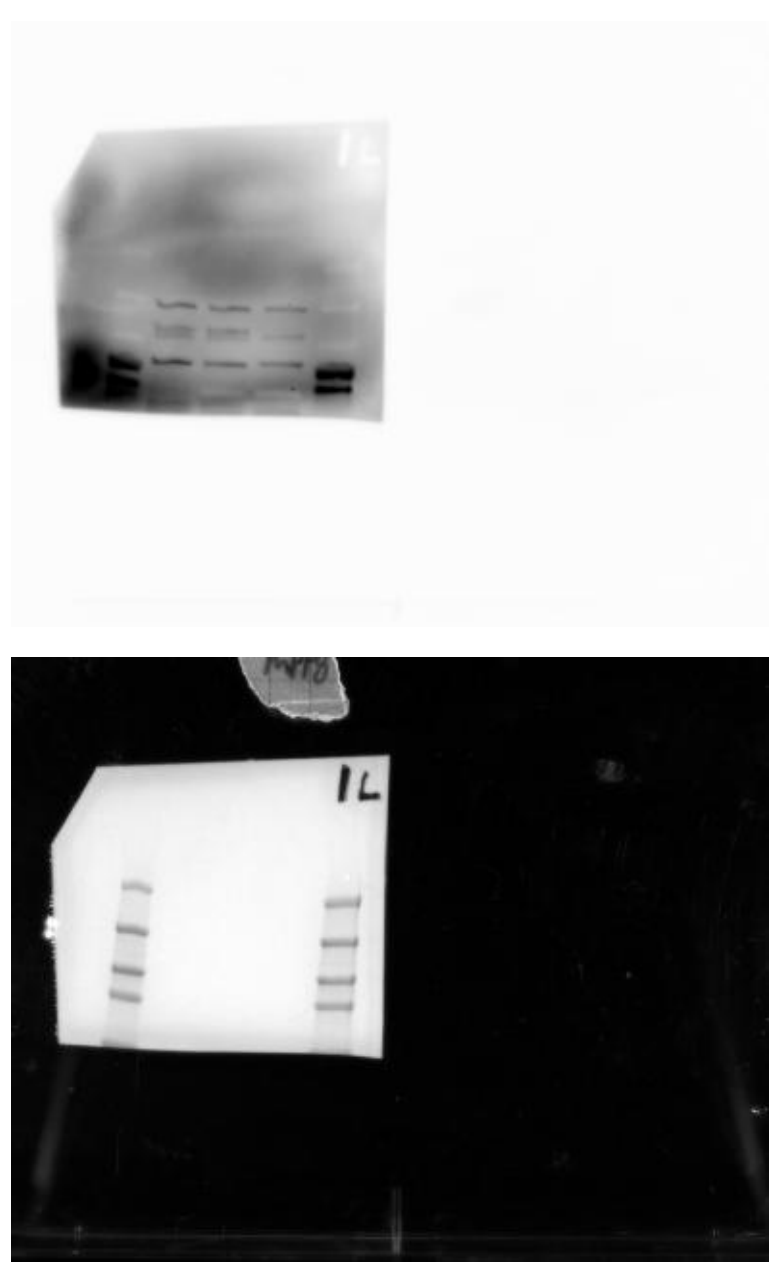

Top = MPP8 (1A)
Middle = IFIT1 (1G)
Bottom = ISG15 (1H)

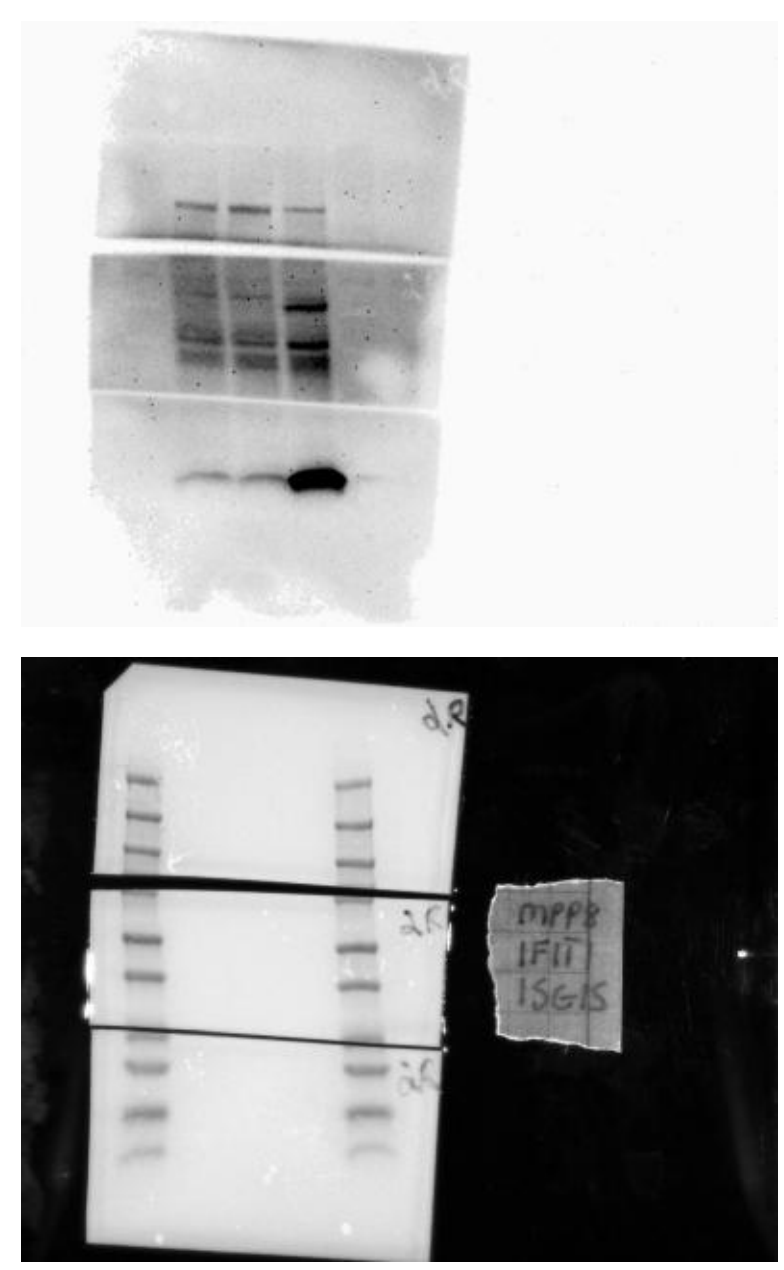

OCT4 (1B)

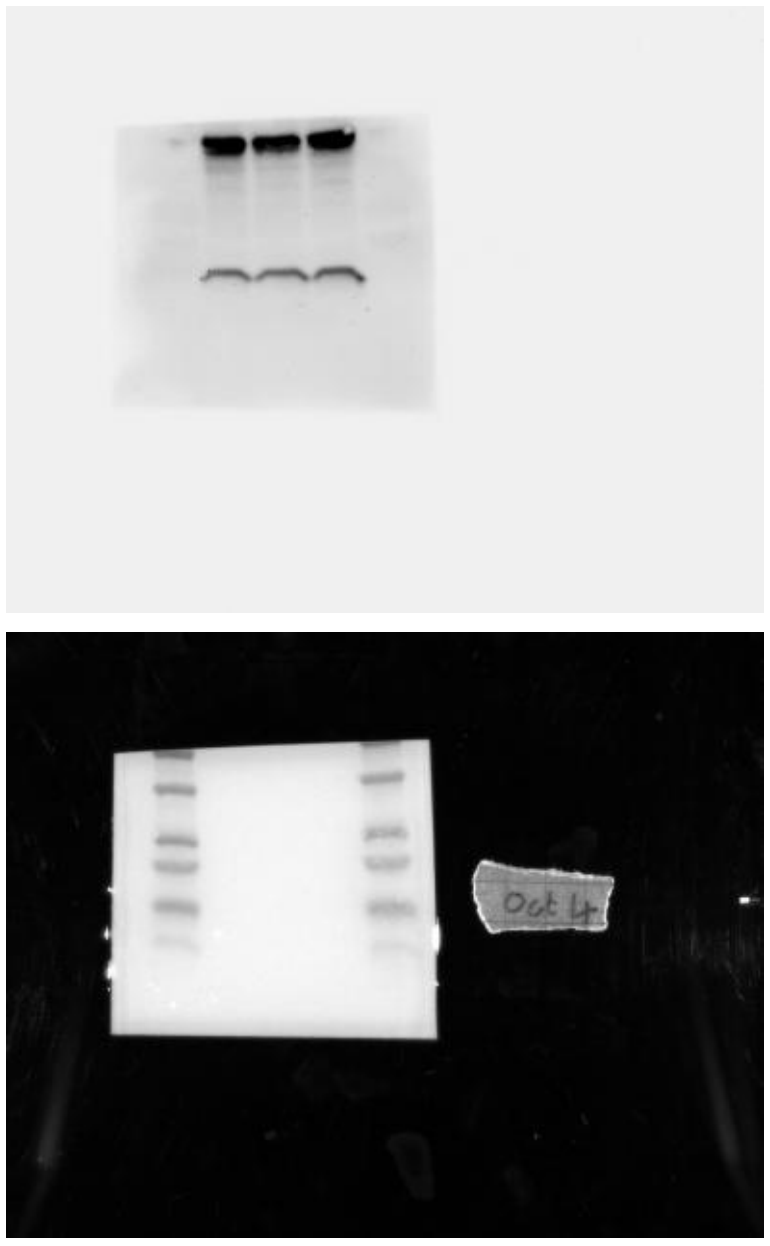

Top = PCNA* (1C)
Bottom = PCNA* (2C)

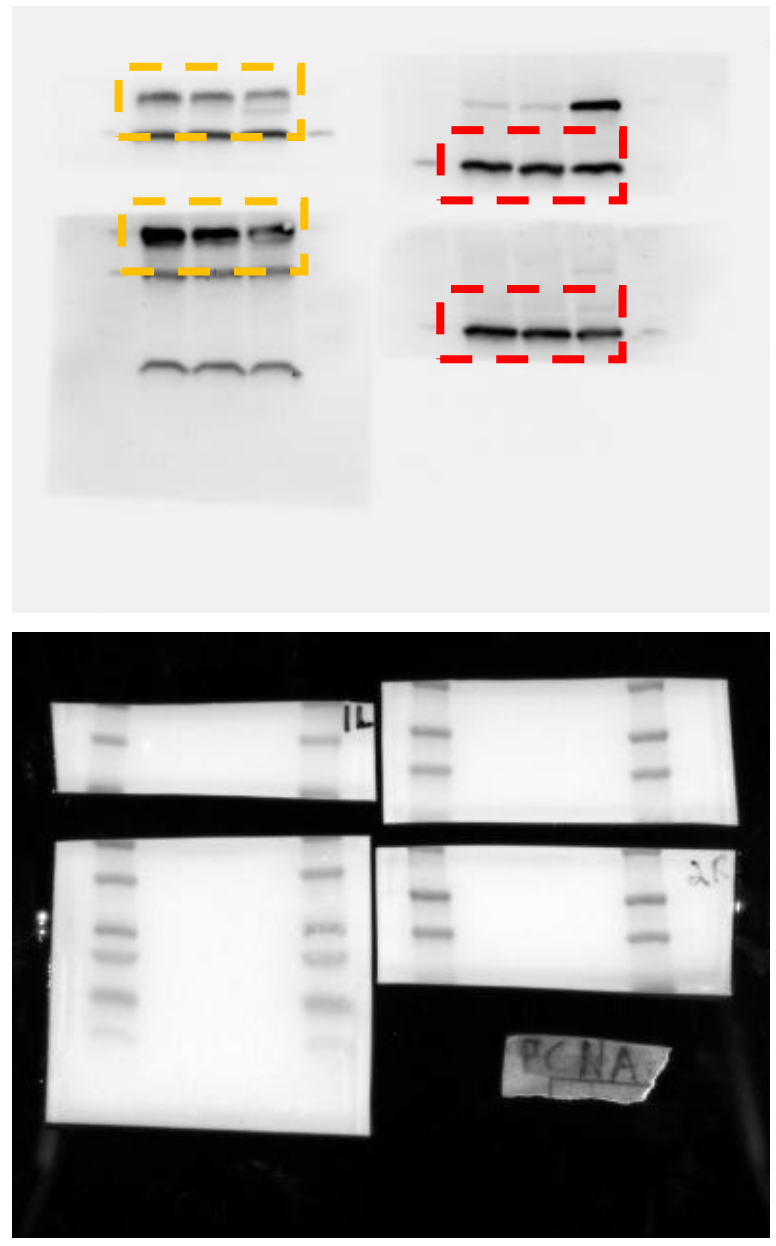

Top Right = MDA5 (1F)
Bottom Left = MPP8 (NI)
Bottom Right = MPP8 (1A)

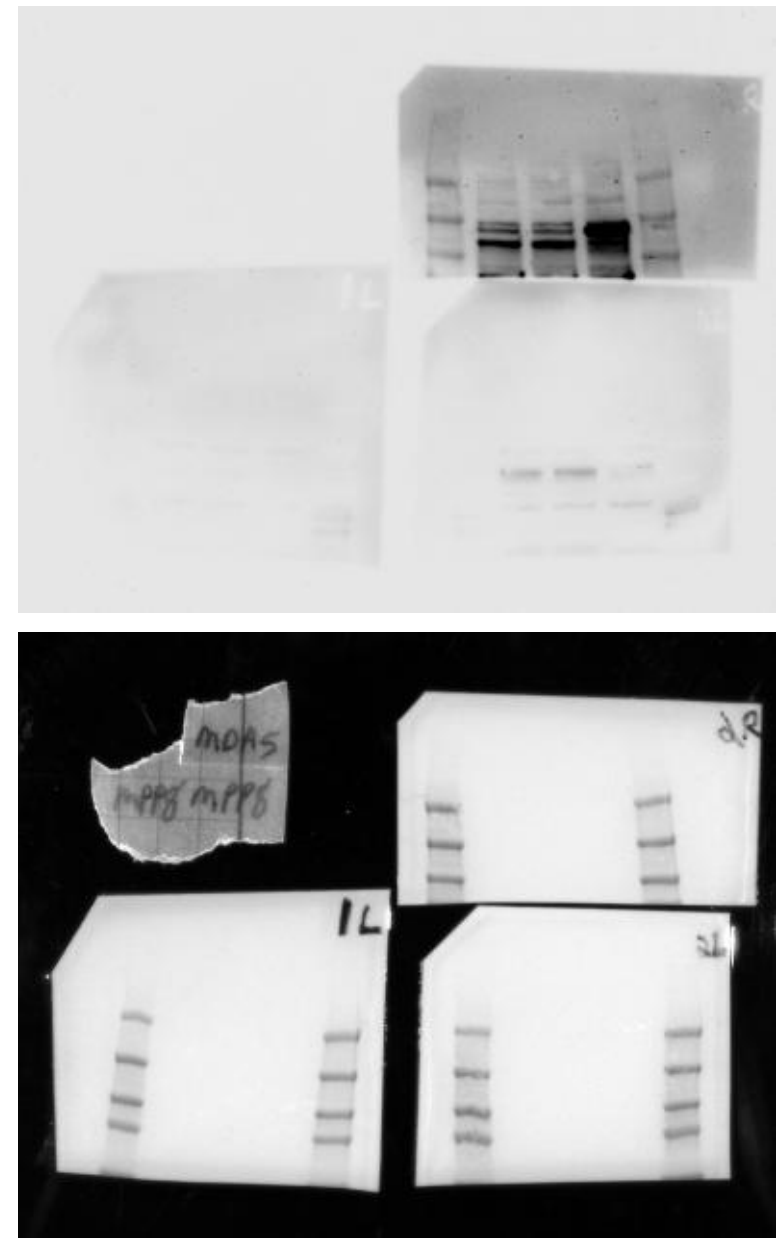

ISG15 (3H)

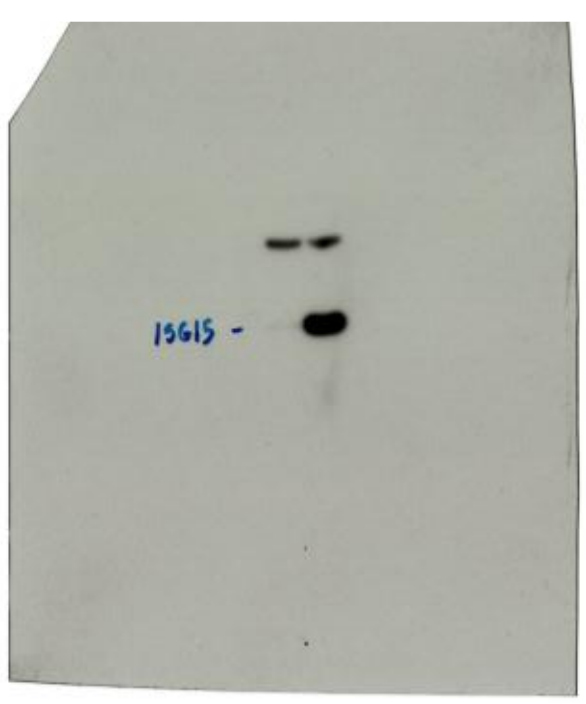

Top = MPP8 (3A)
Bottom = GAPDH (3L)

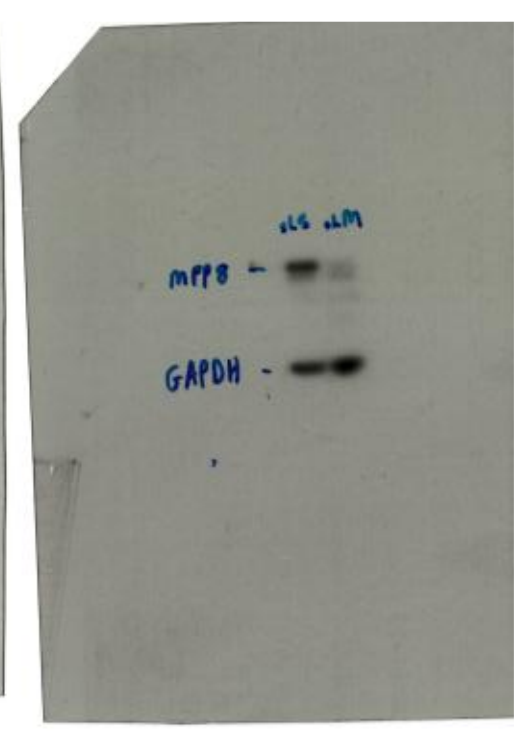

Top = ORF1 (2E)
Bottom = ORF1 (1E)

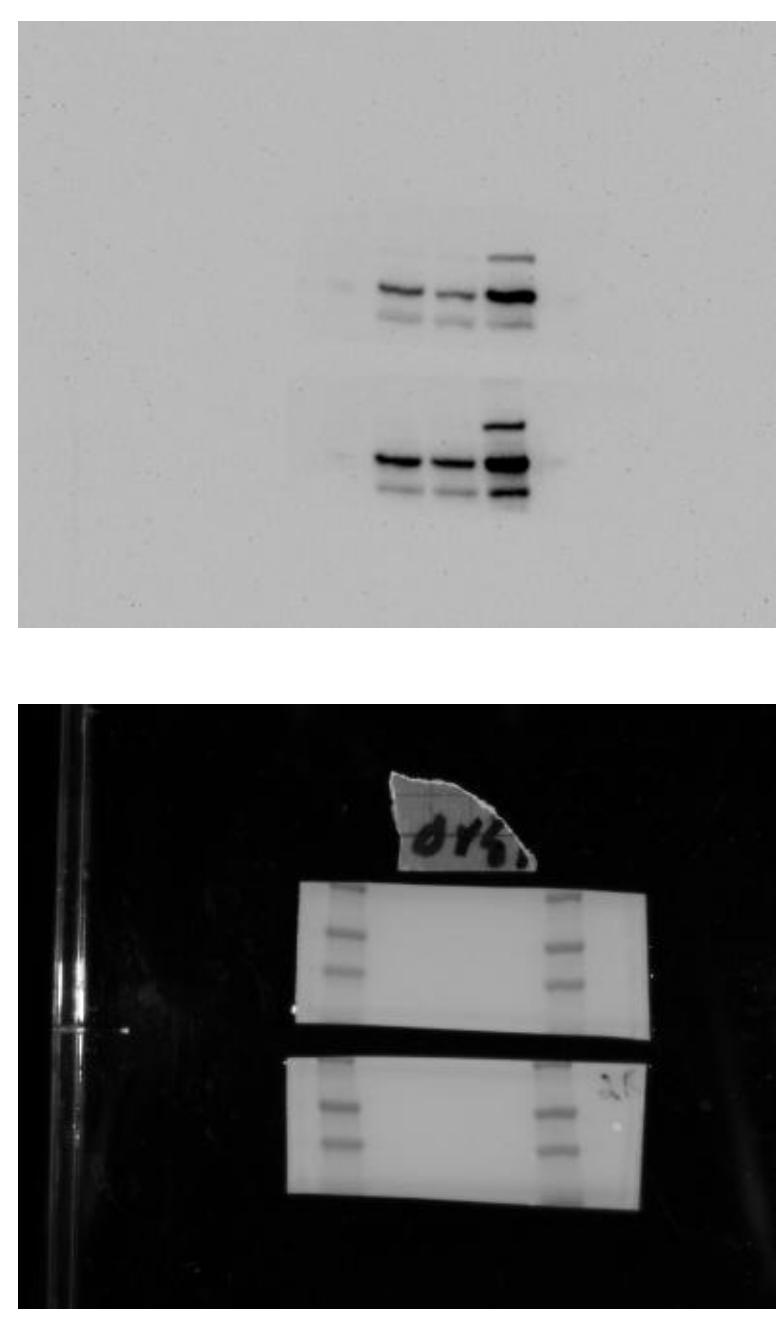

IFIT1 (3G)

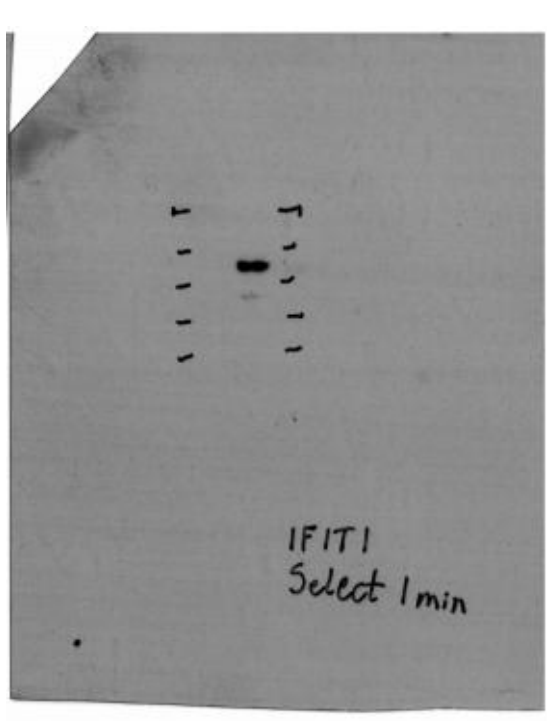

L1 ORF1 (3E)

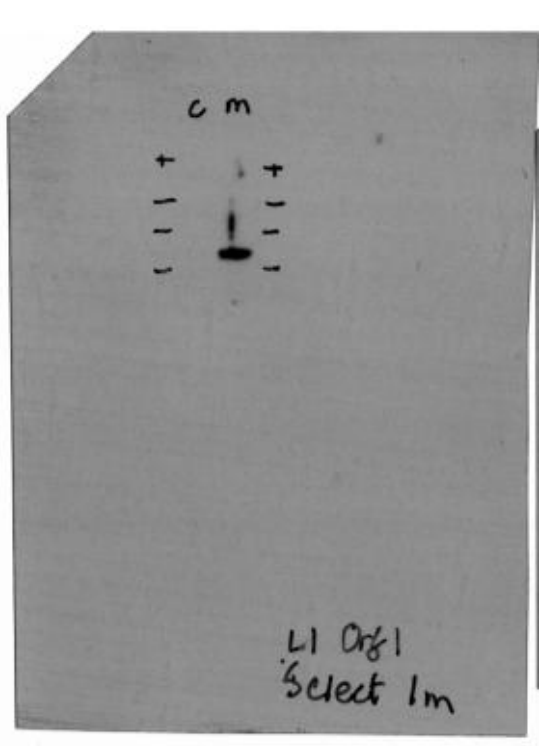

H3 (3M)

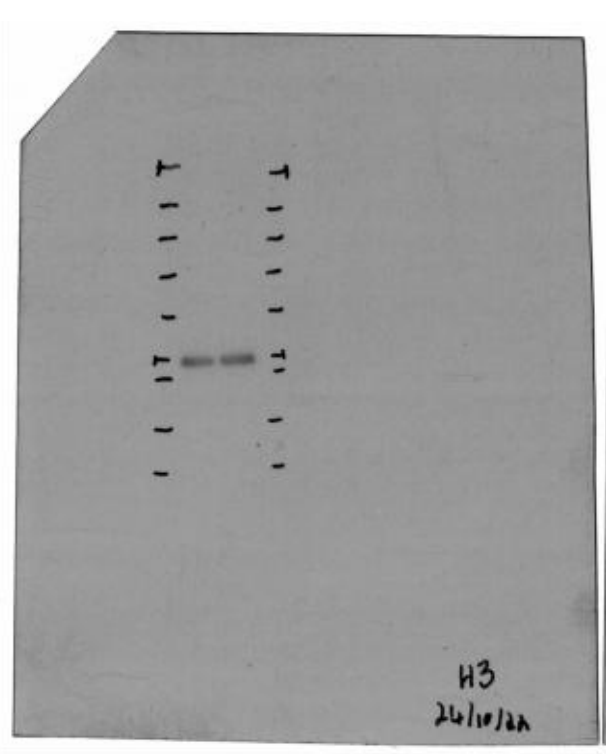

p-IRF (NI)

Top = OCT4 (3B)
Bottom = ISG15 (3I) (over OCT4)

Tubulin* (over p-IRF) (3J)

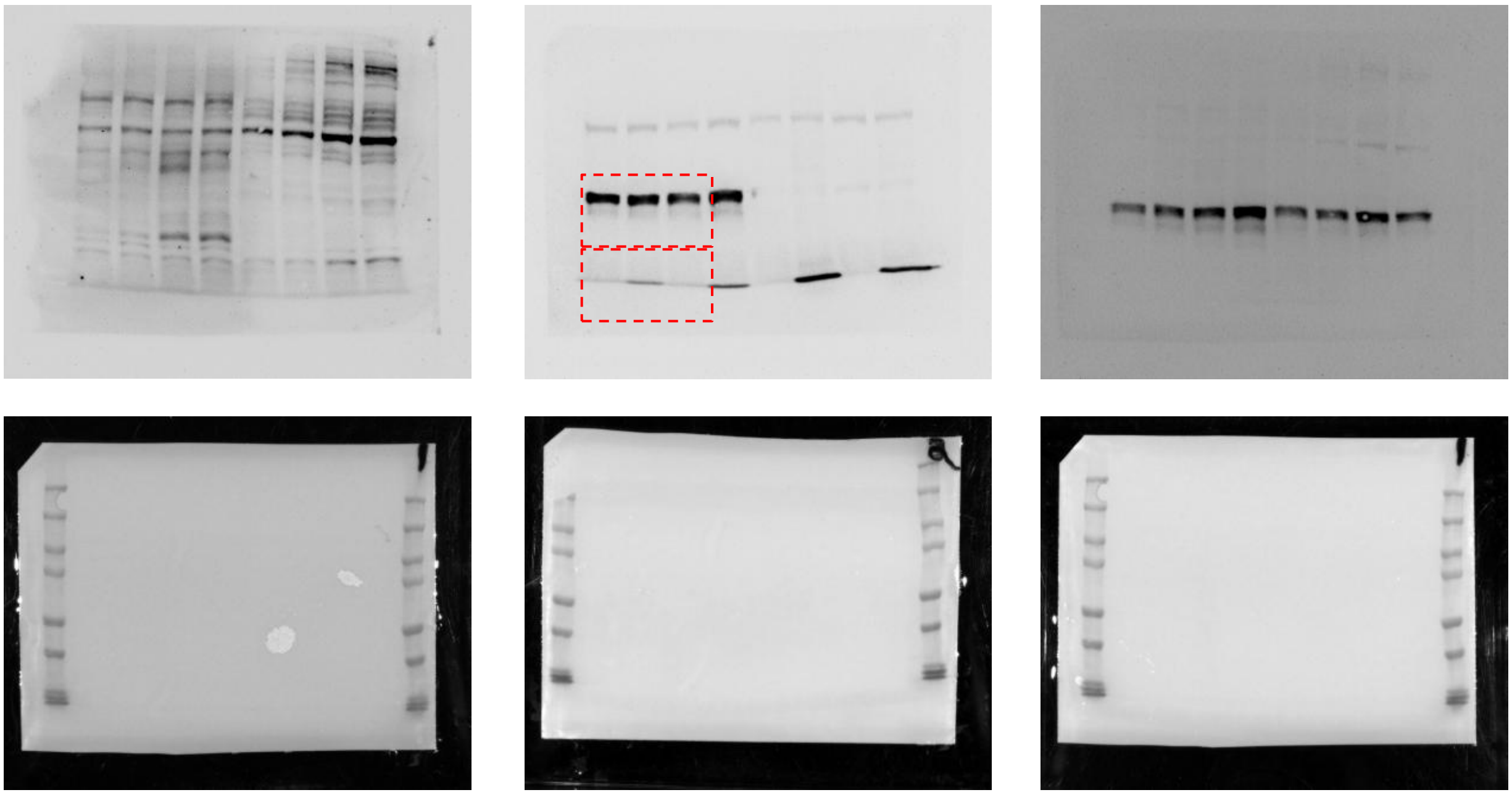

These blots were performed twice per replicate. Both blots were loaded with the same quantity of lysate.

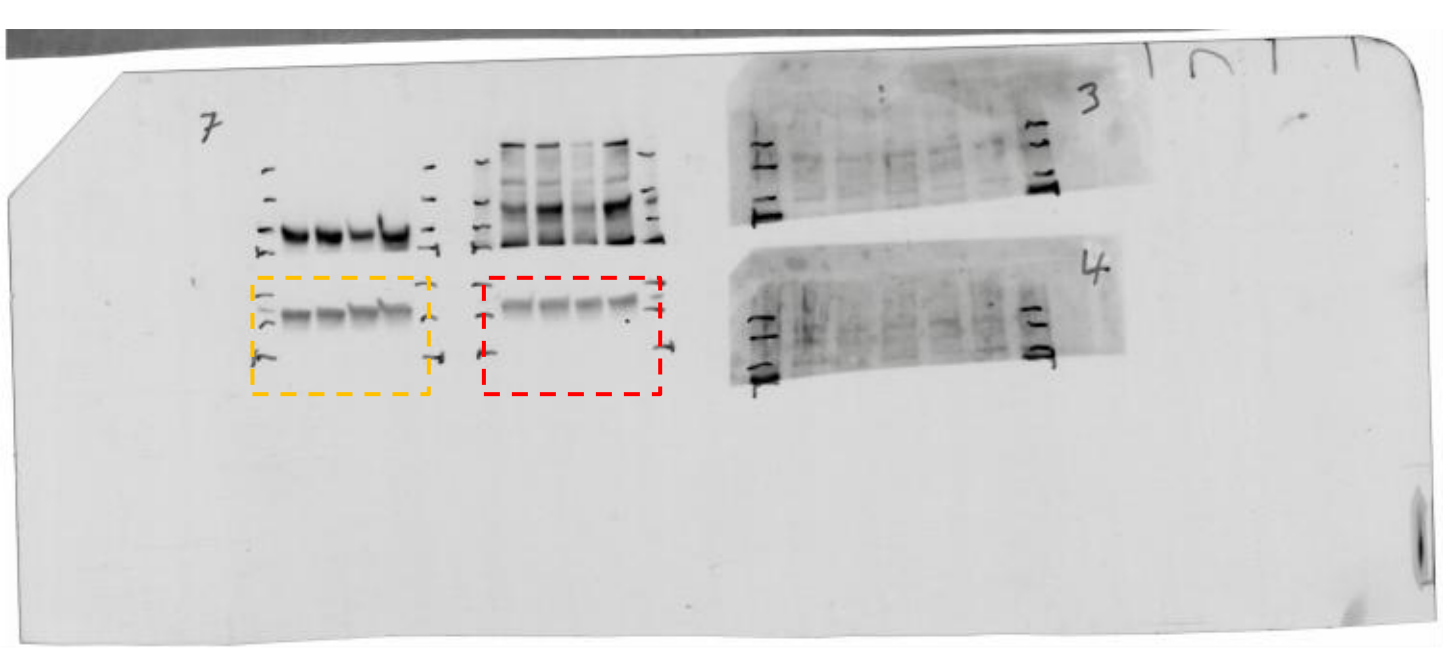

Left = Tubulin* (NI)
Right = Tubulin* (3K)

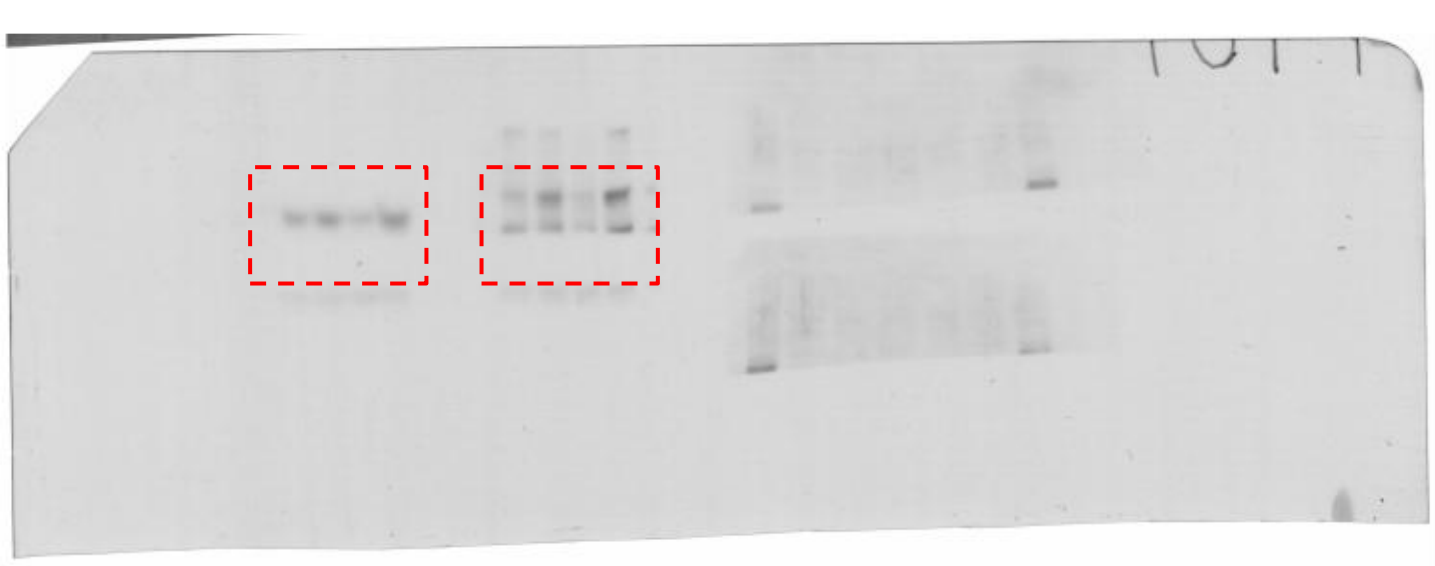

Left = RIGI (3D)
Right = MDA5 (3F)

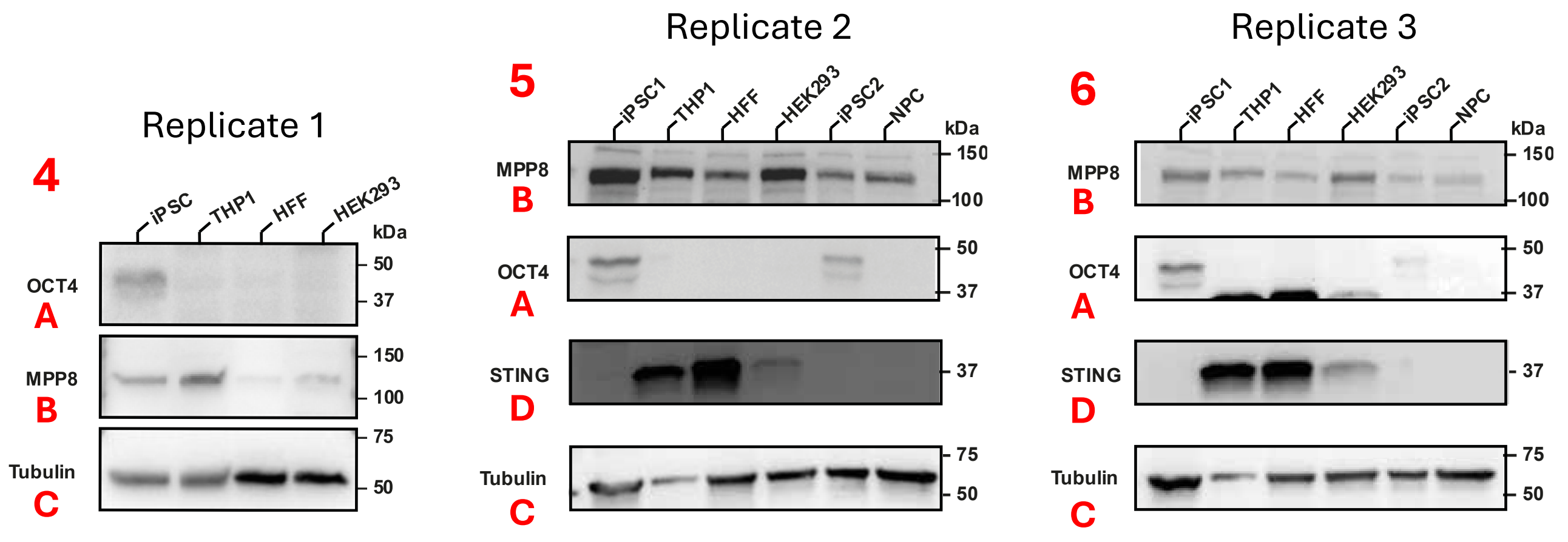

Top = MPP8 (5B)
Bottom = OCT4 (5A)

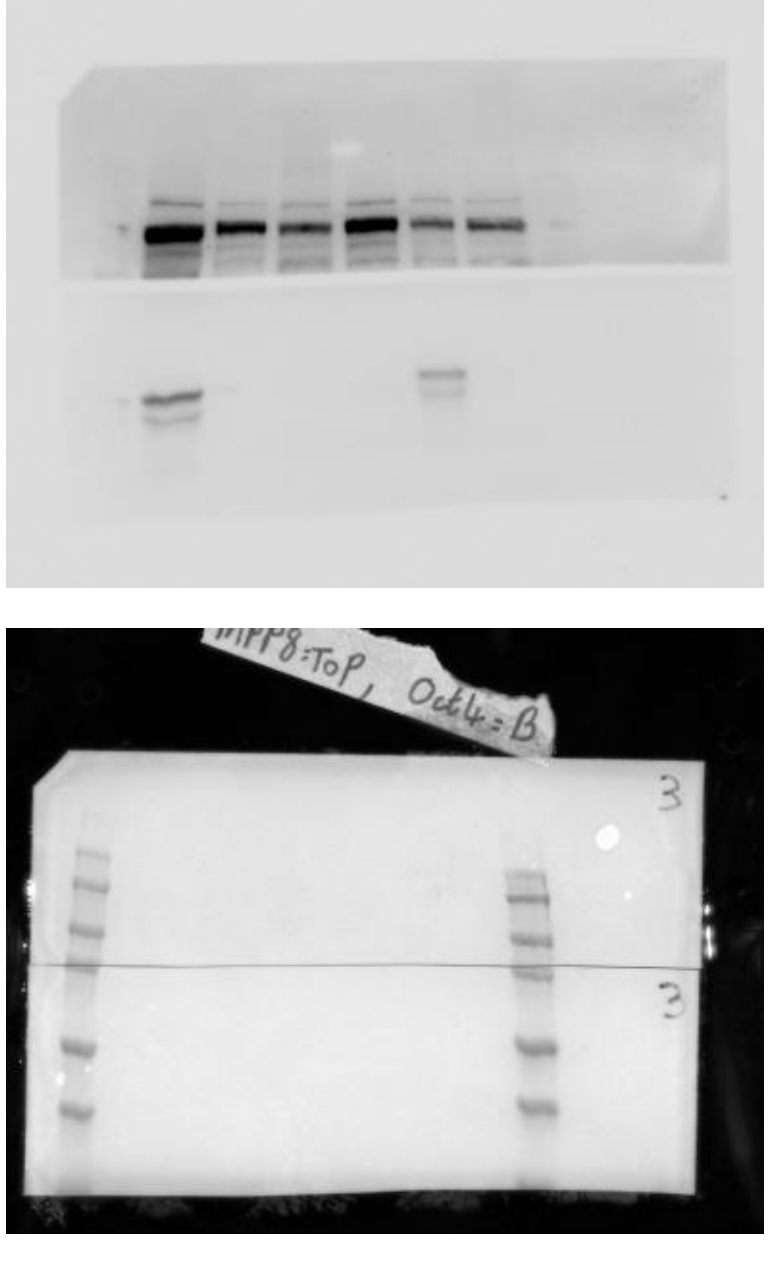

Top = MPP8 (6B)
Bottom = STING (6E)

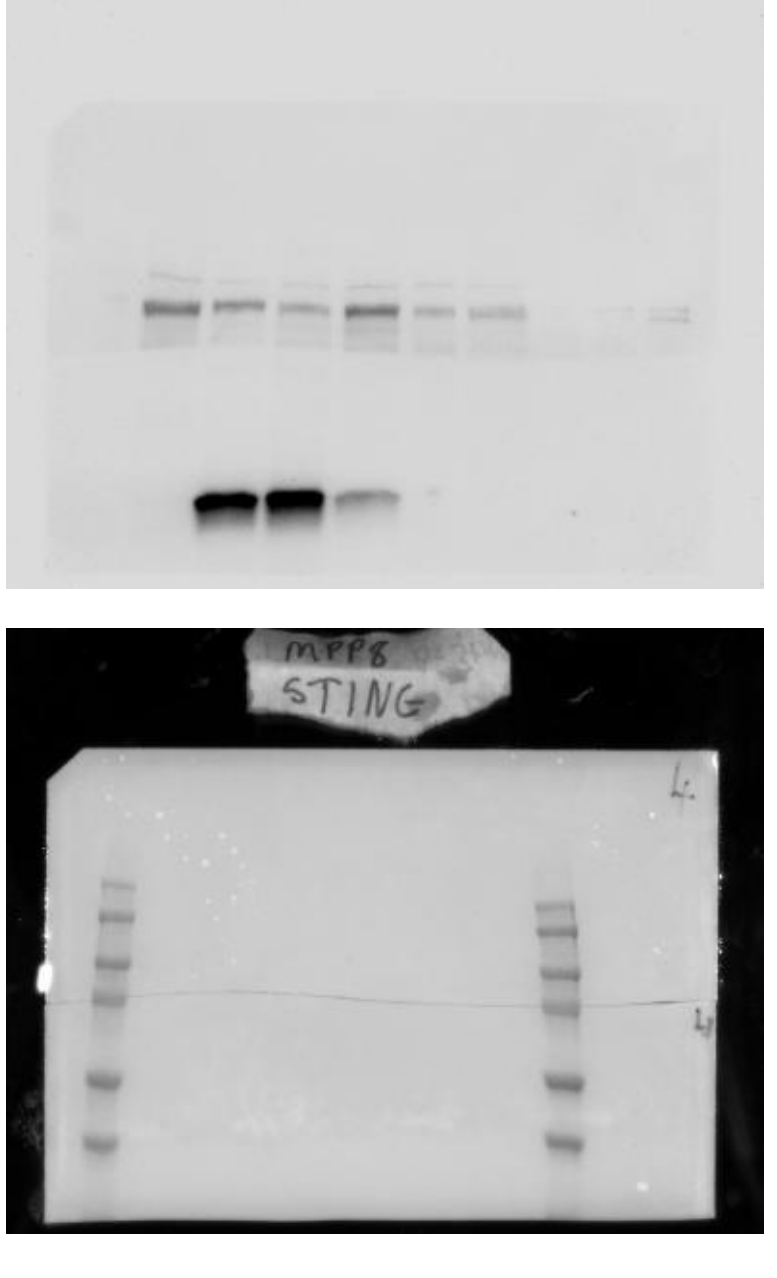

Top = STING (over OCT4) (NI)
Bottom = OCT4 (6A) (over STING)

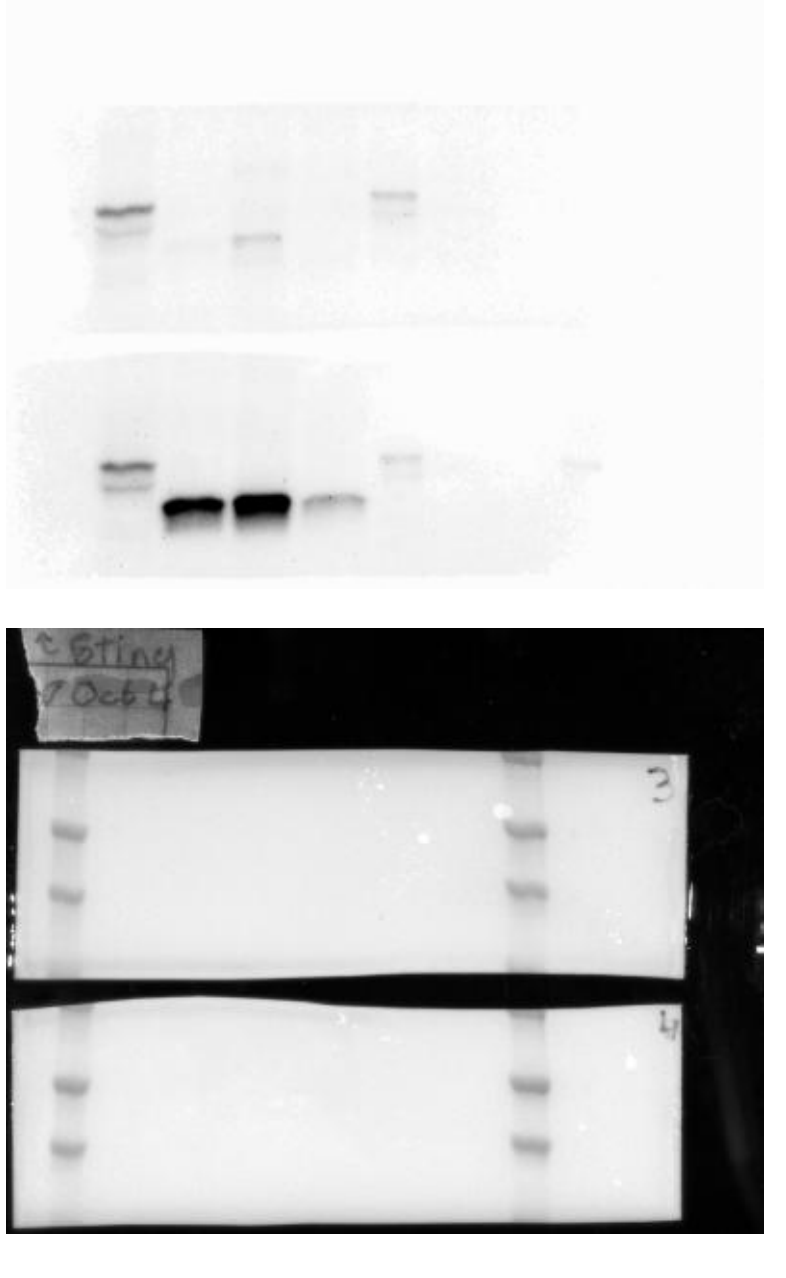

STING (5D)
(over OCT4, repeated)

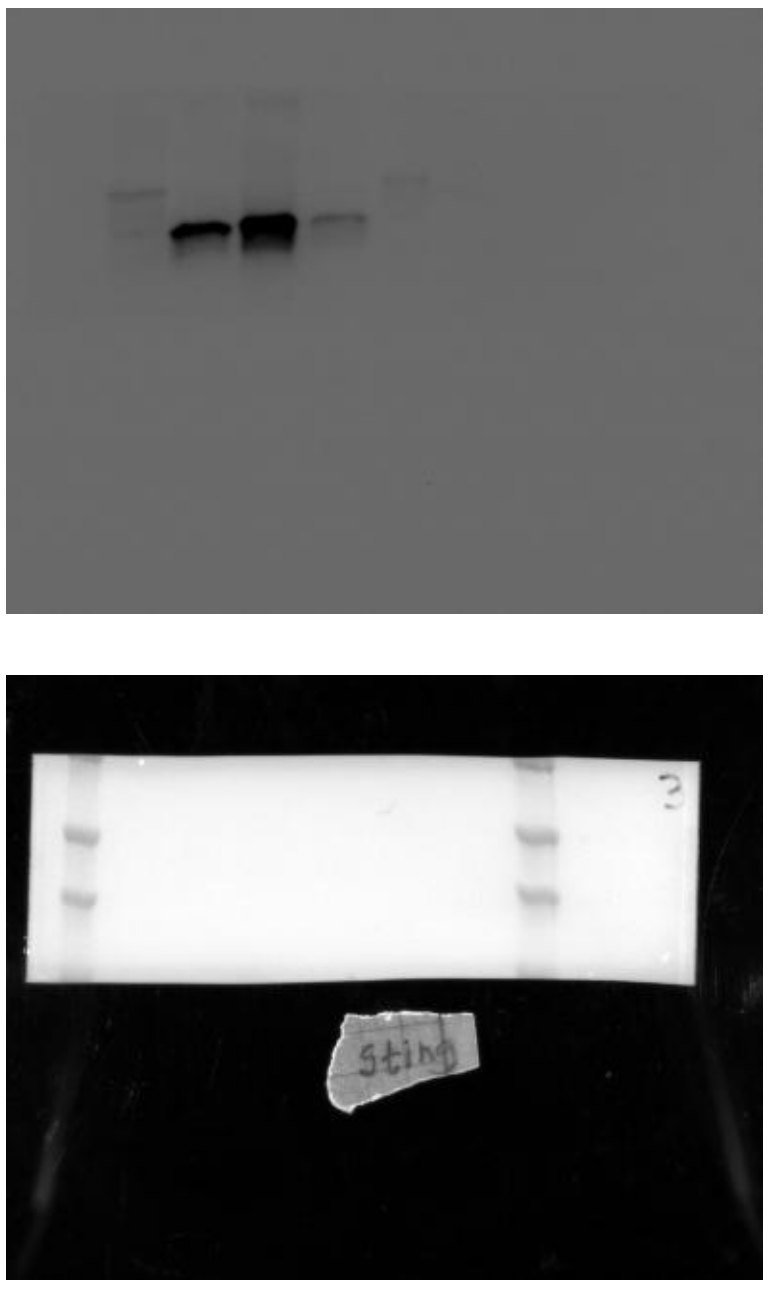

Top = PCNA (over STING) (NI)
Bottom = PCNA (over STING) (NI)

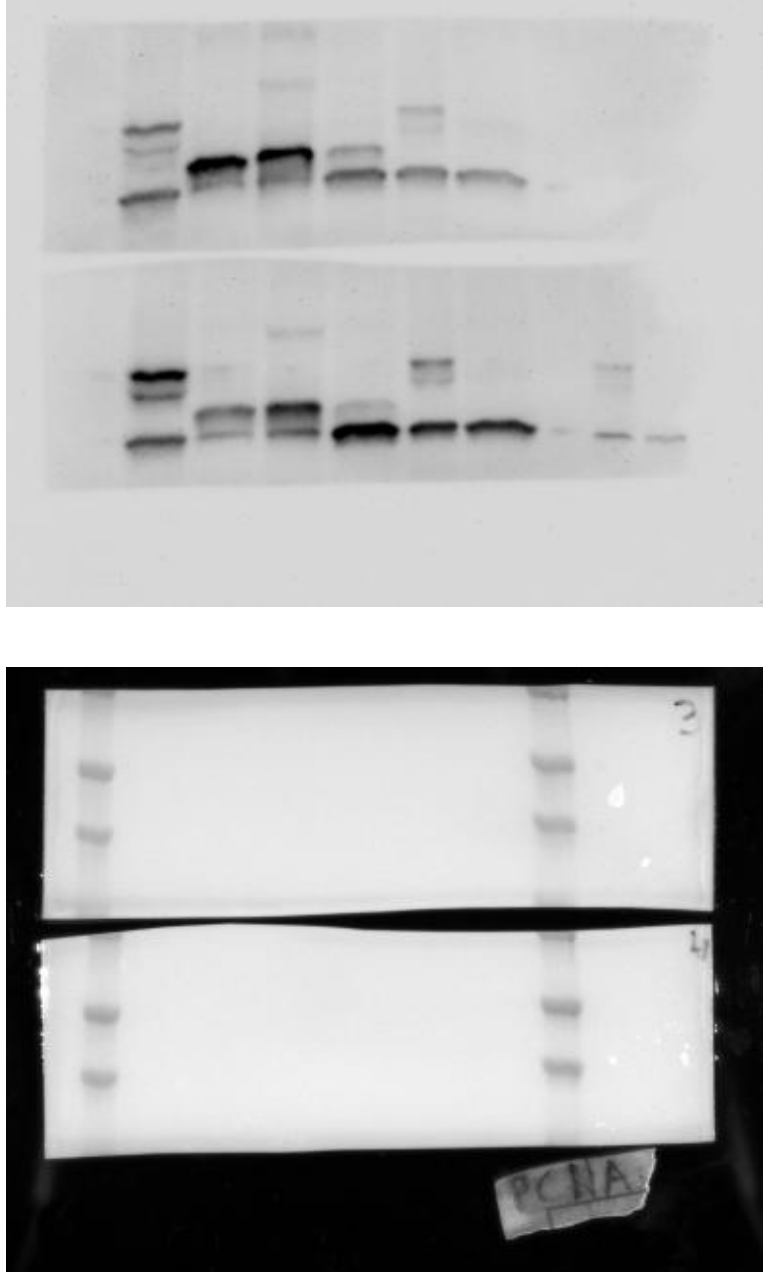

Top = Tubulin 5C
Bottom = Tubulin 6C

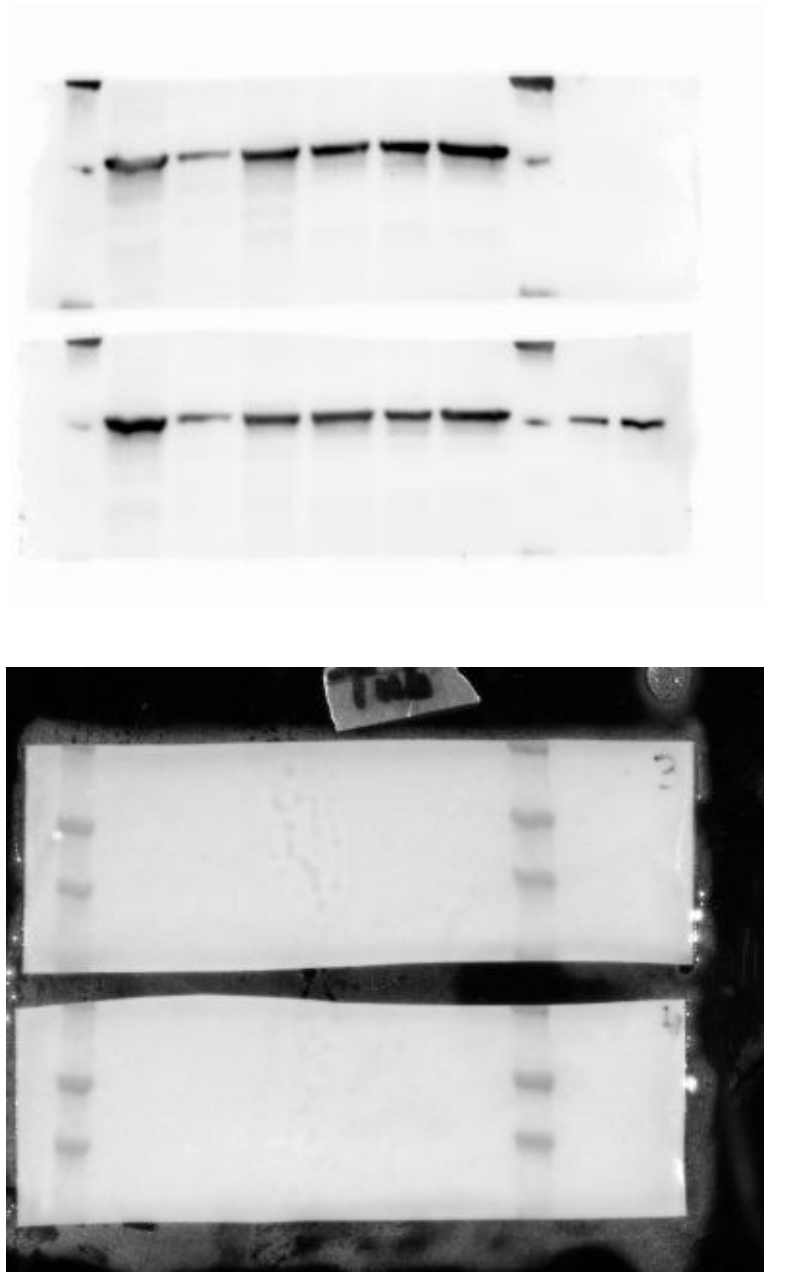

Left = ARL5B (NI)
Right = IRF3 (NI)

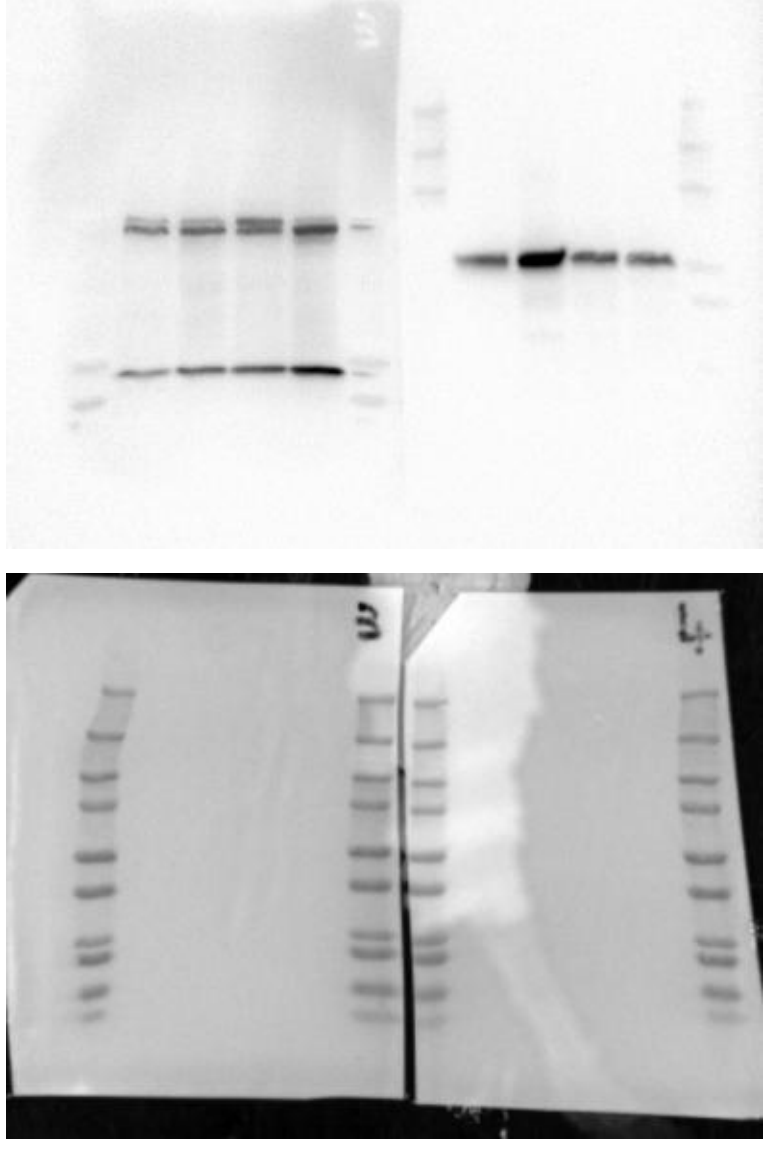

Left = OCT4 (over ARL5B) (4A)
Right = MPP8 (over IRF3) (4B)

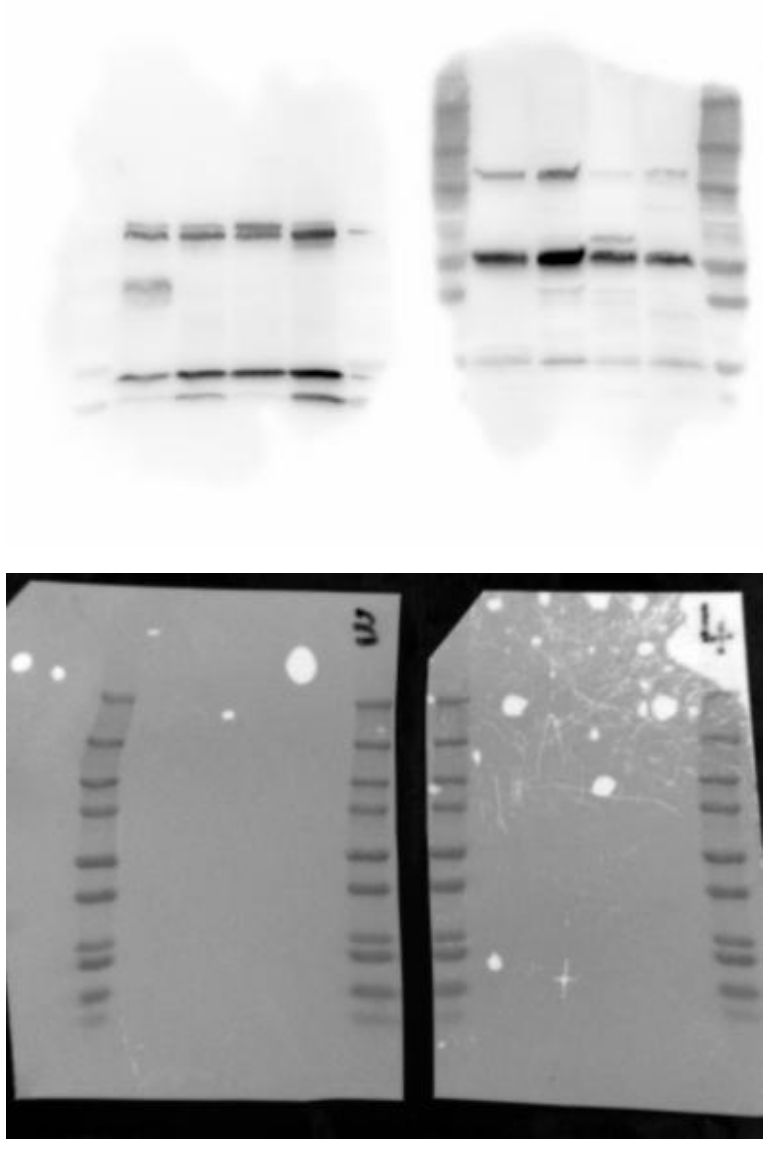

Left = Tubulin* (NI)
Right = Tubulin* (4C)

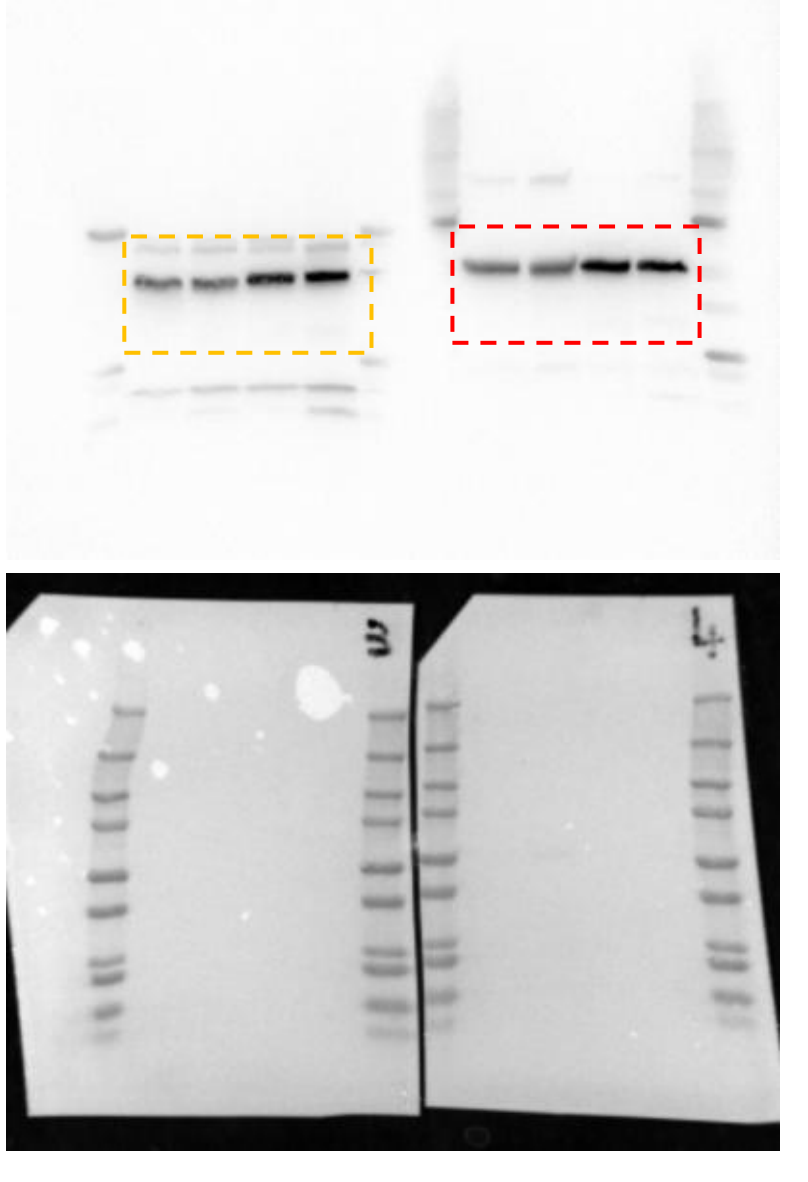

Supplement: Supplementary file 4 [file 41467_2026_74147_MOESM5_ESM.pdf]
